# Maternal High Fat Diet and Diabetes Disrupts Transcriptomic Pathways That Regulate Cardiac Metabolism and Cell Fate in Newborn Rat Hearts

**DOI:** 10.3389/fendo.2020.570846

**Published:** 2020-09-17

**Authors:** Claudia C. Preston, Tricia D. Larsen, Julie A. Eclov, Eli J. Louwagie, Tyler C. T. Gandy, Randolph S. Faustino, Michelle L. Baack

**Affiliations:** ^1^Genetics and Genomics Group, Sanford Research, Sioux Falls, SD, United States; ^2^Environmental Influences on Health and Disease Group, Sanford Research, Sioux Falls, SD, United States; ^3^Department of Pediatrics, Sanford School of Medicine of the University of South Dakota, Sioux Falls, SD, United States

**Keywords:** PI3K/Akt pathway, mitochondrial biogenesis, cardiovascular disease, maternal diabetes, high fat diet, functional genomics

## Abstract

**Background:** Children born to diabetic or obese mothers have a higher risk of heart disease at birth and later in life. Using chromatin immunoprecipitation sequencing, we previously demonstrated that late-gestation diabetes, maternal high fat (HF) diet, and the combination causes distinct fuel-mediated epigenetic reprogramming of rat cardiac tissue during fetal cardiogenesis. The objective of the present study was to investigate the overall transcriptional signature of newborn offspring exposed to maternal diabetes and maternal H diet.

**Methods:** Microarray gene expression profiling of hearts from diabetes exposed, HF diet exposed, and combination exposed newborn rats was compared to controls. Functional annotation, pathway and network analysis of differentially expressed genes were performed in combination exposed and control newborn rat hearts. Further downstream metabolic assessments included measurement of total and phosphorylated AKT2 and GSK3β, as well as quantification of glycolytic capacity by extracellular flux analysis and glycogen staining.

**Results:** Transcriptional analysis identified significant fuel-mediated changes in offspring cardiac gene expression. Specifically, functional pathways analysis identified two key signaling cascades that were functionally prioritized in combination exposed offspring hearts: (1) downregulation of fibroblast growth factor (FGF) activated PI3K/AKT pathway and (2) upregulation of peroxisome proliferator-activated receptor gamma coactivator alpha (PGC1α) mitochondrial biogenesis signaling. Functional metabolic and histochemical assays supported these transcriptome changes, corroborating diabetes- and diet-induced cardiac transcriptome remodeling and cardiac metabolism in offspring.

**Conclusion:** This study provides the first data accounting for the compounding effects of maternal hyperglycemia and hyperlipidemia on the developmental cardiac transcriptome, and elucidates nuanced and novel features of maternal diabetes and diet on regulation of heart health.

## Introduction

Cardiovascular disease (CVD) is the leading cause of death in the United States and by 2030 is projected to affect 40.5% of the US population (1). It is critical to identify high-risk populations and implement targeted prevention in order to decrease this growing burden of disease. The pathogenesis of CVD is influenced over time by both hereditary and environmental factors. Mounting evidence shows that these processes may even begin before birth (2, 3). Specifically, exposure to excess circulating maternal fuels during critical windows of fetal development increases the lifetime risk of CVD (4–7). Worldwide, there are 21.3 million live births annually to women with hyperglycemia during pregnancy (8). Additionally, 35% of women are obese (9), a co-morbidity that exacerbates this growing problem. Indeed, obese women develop gestational diabetes mellitus (GDM) at 4 times higher odds than non-obese women (10). For these reasons, finding effective, targeted prevention for this growing and readily identifiable population would significantly lower the burden of heart disease over time.

While it is increasingly recognized that infants born to diabetic or obese mothers have a higher incidence of heart disease at birth and later in life, prevention is hindered because the underlying mechanisms remain unknown. Importantly, infants exposed to diabetic pregnancy have a higher incidence of cardiac hypertrophy, diastolic dysfunction and impaired myocardial performance that begins *in utero* and cardiac pathology is similar regardless of diabetes type (pregestational or gestational diabetes) (11). To improve overall outcomes of diabetic pregnancy, the current standard of care is routine screening and efforts to optimize maternal blood sugar levels before and during pregnancy (12). While improved glycemic control, especially alongside enhanced and earlier screening for gestational diabetes has certainly decreased perinatal morbidities (13–16), infants continue to have a higher risk of heart disease even when born to mothers with good glycemic control (11, 17–20). Additionally, gestational diabetes predisposes infants to macrosomia and programmed cardiometabolic disease as adults, even if their mother was treated during pregnancy (21–28). This suggests additional under-recognized, targetable risk factors including lipids. Both maternal diabetes and high fat (HF) diet increase circulating lipids above the normal physiologic hyperlipidemia of pregnancy (29). We developed a rat model to determine the individual and compounding effects of maternal diabetes and HF diet on cardiac outcomes in offspring. We found that a triad of maternal hyperglycemia, hyperlipidemia, and fetal hyperinsulinemia led to progressively worsening mitochondrial dysfunction, impaired cellular bioenergetics, and poorer cardiac function in newborn offspring hearts (30, 31).

We hypothesized that exposure to excess circulating fuels disrupts the *in utero* gene-environment interaction to program heart disease in the developing fetus, specifically through metabolic and mitochondrial mediated mechanisms. Our previous data using a well-characterized rat model and chromatin immunoprecipitation sequencing (ChIP-Seq) showed that maternal HF diet, especially alongside late-gestation hyperglycemia, caused distinct fuel-mediated epigenetic programming of cardiac metabolism during fetal cardiogenesis (32). The present study used a cardiac systems biology approach that uncovered specific mechanisms underlying cardiometabolic pathology that may serve as potential targets for intervention.

## Materials and Methods

All experimental methods were carried out in agreement with applicable international, national, and institutional guidelines for the care and use of animals (Animal Welfare Act and National Institutes of Health policies) and were approved by the Sanford Research Institutional Animal Care and Use Committee. Sprague Dawley rats (Harlan Laboratories, Indianapolis, IN) were used in all experiments and housed in Sanford Research's Animal Resource Center, a climate-controlled, light-dark cycled facility.

### Animal Model Characteristics

**Figure 1:**
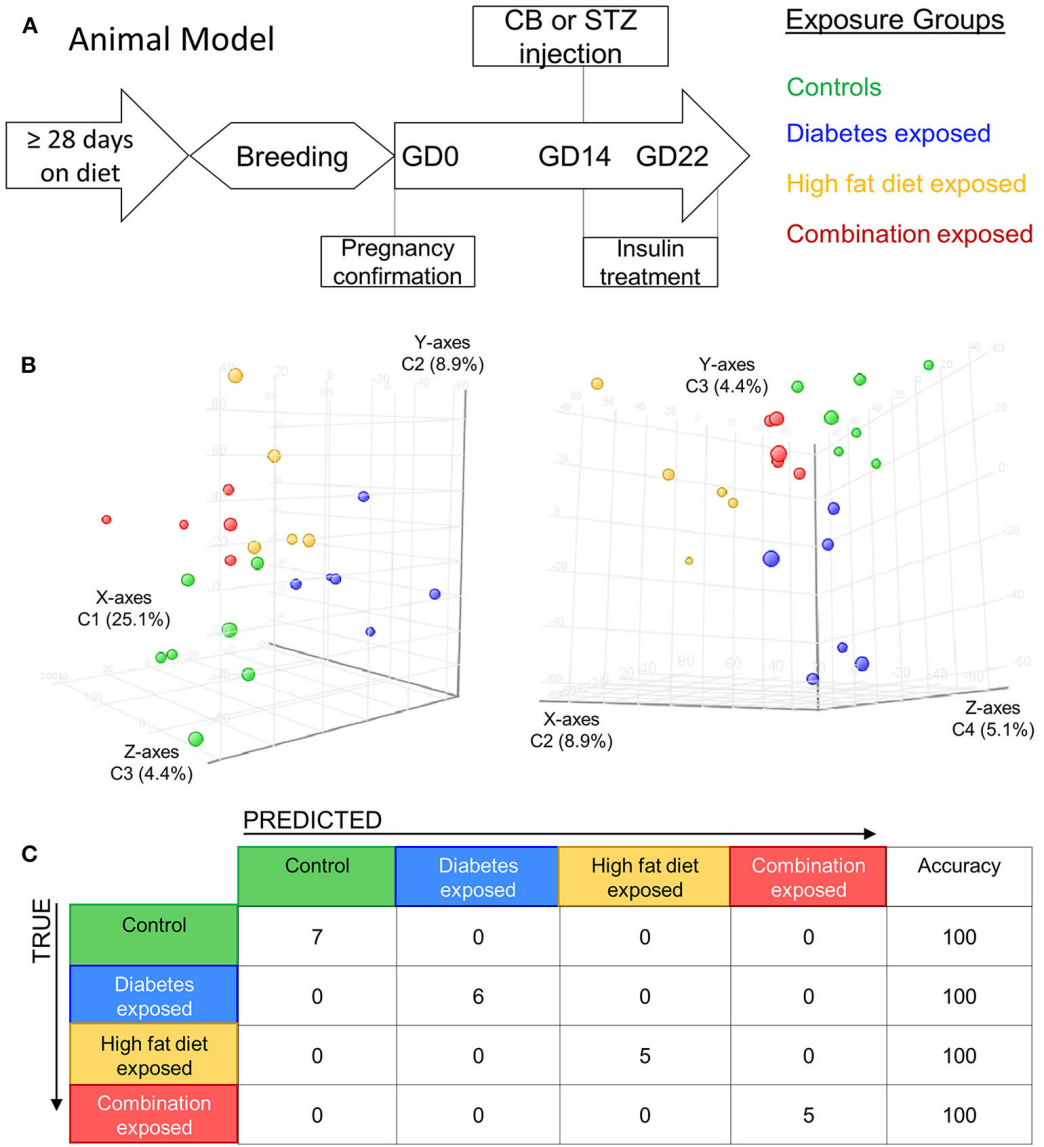
Maternal diabetes and maternal high fat diet impart distinct cardiac transcriptome signatures in newborn rat offspring. **(A)** Schematic showing experimental model of exposed groups. Female rats had at least 28 days of either control or high-fat diet prior to breeding. Female diet continued throughout pregnancy. At gestational day (GD) 14 a single injection of citrate buffer (CB) or streptozotocin (STZ) was delivered to a subset of females with high-fat or control diet. At GD22 newborns were delivered and hearts were extracted for study from four exposed groups: controls (green), diabetes exposed (blue), high fat diet exposed (yellow), and combination exposed (orange). **(B)** Principal components analysis (PCA) plots depicting distinct transcriptome signature among all the exposed groups. Data is visualized here in two plots with different components plotted in each: components 1–3 are plotted on the left (C1 = 25.1%, C2 = 8.9%, and C3 = 4.4%); and components 3–4 are used in PCA plot on right (C2 = 8.9%, C3 = 4.4% and C4 = 5.1%). **(C)** Class prediction model used individual sample expression signatures to demonstrate with 100% accuracy the identification of each sample to their respective exposure group.

Methods and model characteristics of the four animal groups used in this study have been detailed previously (30, 33). Briefly, young adult female rats received either control or HF diet (Teklad, Harlan Laboratories, Madison, WI) for at least 28 days before mating and throughout pregnancy. Gestational day zero (GD0) was determined by a positive vaginal swab for spermatozoa. On GD14, after confirmation of pregnancy through ultrasound, dams received either citrate buffer (0.09 M) or 65 mg/kg of intraperitoneal streptozotocin (Sigma Life Sciences, St. Louis, MO) to induce diabetes in the last third of pregnancy. The model consistently exposes the developing fetus to a triad of maternal hyperglycemia, diet-induced hyperlipidemia, or the combination (glucolipotoxicity) which incites directly proportional levels of fetal hyperinsulinemia, respectively. The timing of diabetes induction in the last 1/3^rd^ of pregnancy is intentional to exclude confounding from hyperglycemia induced disruption in ovulation, placentation, and organogenesis. Additionally, timing corresponds with peak placental lipid accumulation, fetal pancreatic endocrine function, and more closely translates to pregnancies affected by gestational diabetes in the last trimester. Hyperglycemia was partially controlled with twice daily insulin treatments using regular insulin in the morning and insulin-glargine in the evening to keep non-fasting, whole blood glucose levels in a targeted non-fasting, pre-treatment range of 200–400 mg/dl. This target range was selected to assure an ample hyperglycemic exposure to diabetes-exposed pups, but also avoid significant ketoacidosis or dehydration. Dams that received streptozotocin but did not manifest a fasting blood glucose level ≥ 200 mg/dl were excluded from the study. While in our model all pregnant dams developed physiologic hyperlipidemia of pregnancy, diabetes increased serum triglycerides ~2 fold, diet ~3 fold, and the combination ~5 fold higher than controls leading to corresponding hyperinsulinemia in offspring (30, 32–34). Thus, our model of late gestation diabetes plus HF diet translates to poorly controlled gestational diabetes or Type 2 diabetes in the developing offspring with exposure to glucolipotoxicity and fetal hyperinsulinemia. Delivery (~ GD22) yielded postnatal day one (P1) offspring from four distinct groups: exposed to maternal diabetes alone, exposed to maternal HF diet alone, exposed to the combination of both maternal diabetes and HF diet, and control group (Figure 1A). We used all four groups for gene expression analysis with further experiments streamlined to males from control and combination exposed groups.

### Total RNA Isolation and Quantification

Hearts were extracted from control, diabetes exposed, HF diet exposed, and combination exposed P1 offspring. Immediately after harvesting, samples were snap frozen in liquid nitrogen, and stored at −80°C until RNA extraction. Each experimental group consisted of a pool of male and female rat hearts. Total RNA was extracted from the whole heart with TRIzol and purified using an affinity resin column (Qiagen RNeasy Mini kit, Germantown, MD) according to manufacturer's protocol. Total RNA concentration was performed using spectrophotometric analysis measurement (abs-emission A260/A280) by NanoDrop 2000 UV-Vis Spectrophotometer (Thermo Fisher Scientific Inc. Waltham, MA). RNA sample integrity was assessed by electropherogram analysis on an Agilent 2100 Bioanalyzer (Agilent Technologies, Santa Clara, CA) and only samples with RNA integrity number (RIN) scores > 8 were used for microarray labeling and hybridization.

### Microarray Hybridization and Data Analysis

Microarray hybridization was performed by the Analytical Genomics Core Facility (Sanford Burnham Medical Discovery Institute, Lake Nona, Orlando, FL) using GeneChip Rat Gene 1.0 ST arrays (Affymetrix, Santa Clara, CA) according to manufacturer's protocol. Briefly, total isolated RNA (100 ng) from each sample was converted to cDNA utilizing SuperScript III First Strand Synthesis Supermix (Invitrogen, Life Technologies Corporation, Carlsbad, CA). Labeled complimentary RNA (cRNA), synthesized and amplified from the double-stranded cDNA template, was fragmented and hybridized onto GeneChip arrays. As a measure of quality control of the fragmented biotin-labeled cRNA, a prior hybridization of a test-3 array was performed and analyzed. GeneChip 3000 scanner (Affymetrix, Santa Clara, CA) was used to scan and quantitatively analyze images of hybridized GeneChip arrays. Intensity values for each probe cell in the arrays were calculated by GeneChip software.

Data normalization and analysis was performed using GeneSpring GX 14.01 (Agilent Technologies, Palo Alto, CA). Probe cell intensities were used to calculate an average intensity for each set of probe pairs representing a gene. Quality control (QC) filtering was performed on the normalized intensity values and entities were clustered into four conditions: diabetes, HF diet, combination exposed and controls. Gene expression profiles for each condition were visualized as volcano plots to identify genes significantly upregulated or downregulated in each group.

### Functional Annotation Analysis

Statistically significant gene expression profiles from each comparison were separated into upregulated and downregulated lists for functional annotation, Kyoto Encyclopedia of Genes and Genomes (KEGG), and Reactome pathway enrichment analysis using the Database for Annotation, Visualization and Integrated Discovery (DAVID) Bioinformatics Resources v6.8 (https://david.ncifcrf.gov/, last access on 7/24/2020). To determine over representation or enrichment, the DAVID algorithm employs a modified Fisher's exact test that is incorporated into a score that reports relative priority. Entrez Gene identifiers of differentially expressed genes (structured into three lists: upregulated, downregulated and total genes changing) were submitted to DAVID for functional annotation analysis. RaGene-1_0-st-v1 array gene set was used as background and a high classification stringency was selected to maintain robust groups. Scores were reported for KEGG and Reactome pathways when applicable. Further functional pathway analysis of the upregulated, downregulated and total differentially expressed genes list was done through Reactome pathways database analysis tool (Reactome v69; https://reactome.org/, last access on 07/24/2020).

### Networks Analysis and Gene Targets Prioritization

Ingenuity pathway analysis (IPA; Qiagen, Germantown, MD) was performed to map functional gene networks defined by the quality-filtered transcriptome. Highest priority network scores were determined and all the gene relationships, i.e., functional interactions among genes, were exported from IPA for use in Cytoscape v3.7.1 (https://cytoscape.org/) for further network analysis. Prioritization of gene targets was achieved through graph theory analysis tools within Cytoscape. Molecule Activity Predictor Analysis module in IPA was used to predict activation or inhibition of non-focused neighboring molecules, defined by IPA as molecules not included in the uploaded list of genes/molecules, within the functional network. This prediction analysis is based on the expression of the focused molecules, also known as statistically significant genes, within the network and predicts either upstream and/or downstream activities.

### Determination of Mitochondrial-Associated Genes

MitoCarta 2.0 database (Broad Institute, Cambridge, MA) was used to determine the mitochondrial-associated genes in our list of statistically significant genes. MitoCarta 2.0 is an online repository of 1,158 mammalian (human and mouse) genes encoding proteins where their mitochondrial localization has been validated by various methods. We cross-referenced data from MitoCarta with data from the current study to identify mitochondrial-related genes in our gene expression dataset. The recently updated mouse MitoCarta 2.0 database used in the present analysis can be found at the following website: (https://www.broadinstitute.org/files/shared/metabolism/mitocarta/mouse.mitocarta.2.0.html).

### Quantitative RT PCR

RNA was extracted from newborn (P1) rat hearts using the RNeasy Fibrous Tissue Mini kit (Qiagen, Germantown, MD) following manufacturer's protocol. RNA integrity was assessed by electropherograms using 2100 BioAnalyzer (Agilent Technologies, Santa Clara, CA) and demonstrated RIN scores of 9.2-10 (average = 9.8). RNA concentration from two groups, control and combination exposed, was measured by Epoch spectrophotometer (BioTek, Winooski, VT). Complementary DNA (cDNA) was synthesized using iScript cDNA Synthesis Kit and T100 Thermal Cycler (Bio-Rad, Hercules, California). Quantitative PCR (qPCR) was performed by TaqMan Gene Expression Assays approach with ABsolute Blue QPCR Mix (Thermo Fisher Scientific, Waltham, MA) using an ABI7500 qPCR system (Thermo Fisher Scientific, Waltham, MA). Beta-2-microglobulin (*B2m*) was used as the reference gene. Cardiac expression relative to *B2m* was compared between the control and combination exposed groups (*n* = 6 males/group). *B2m*, mitochondrial ribosomal protein L19 (*Mrpl19*), mitochondrial ribosomal protein S27 (*Mrps27*), peroxisome proliferator-activated receptor gamma coactivator 1 alpha (*Ppargc1a*) and fibroblast growth factor receptor 2 (*Fgfr2*) probe/primer sets were obtained from Thermo Fisher Scientific (Waltham, MA), and death associated protein 3 (*Dap3*) probe/primer set was obtained from Integrated DNA Technologies (Coralville, IA).

### Western Blot Analysis

Newborn (P1) rat hearts from control and combination exposed males were homogenized and sonicated in RIPA buffer (50 mM Tris (pH 7.5), 150 mM NaCl, 1% Triton X, 0.5% deoxycholate, 0.1% sodium dodecyl sulfate) with cOmplete protease inhibitor cocktail (Roche, Indianapolis, IN) and phosphatase inhibitor cocktail (Sigma-Aldrich, St. Louis, MO). Protein concentrations were measured using the DC Protein Assay kit (Bio-Rad, Hercules, CA) and Cytation 3 Spectrophotometer (BioTek, Winooski, VT). Protein (20 μg) was prepared using Laemmli buffer and reducing agent then subjected to electrophoresis on 4–15% Criterion TGX Gels using Tris/Glycine/SDS buffer (Bio-Rad). MagicMark XP Western Protein Standard (Thermo Fisher Scientific, Waltham, MA) was used to identify band size. Gels were transferred to PVDF membranes using Trans-Blot Turbo Transfer System (Bio-Rad). Membranes were dried, rehydrated in methanol, washed in TBS, blocked in TBS containing 10% Clear Milk Blocking Buffer (Thermo Fisher Scientific) and then incubated overnight at 4°C with primary antibody. After washing in TBS-T, membranes were blocked again and incubated with secondary antibody for 1 h, using goat anti-rabbit IgG-HRP for reference proteins (Southern Biotech, Birmingham, AL) or donkey anti-rabbit IgG IRDye 680RD (LI-COR, Lincoln, NE) for proteins of interest. HRP exposed bands were visualized using Luminata Forte HRP Chemiluminescence Substrate (Thermo Fisher Scientific). Images were captured using a ChemiDoc MP Imaging System (Bio-Rad) and densitometric analysis was done using ImageJ. Optical density (OD) measurements from tested proteins were normalized to primary reference protein β-ACTIN. Voltage-dependent anion channel or porin (VDAC) and translocase of outer mitochondrial membrane 20 (TOMM20), both outer mitochondrial membrane proteins, were used as secondary references.

### Enzymatic Assays

Insulin and c-peptide levels were measured on 25 μl of newborn (P1) serum using the MILLIPLEX Map Rat Metabolic panel (MilliporeSigma, Burlington, MA) as previously done (30, 33) and Total Protein kinase B isoform 2 (AKT2) and Ser473-phosphorylated AKT2 (regulatory site for insulin signaling) were measured using the MILLIPLEX Map Phospho/Total AKT2 2-plex Magnetic Bead Panel (MilliporeSigma, Burlington, MA) according to manufacturer's protocol as described (33). Briefly, 15 μg of aforementioned newborn (P1) rat heart protein was incubated overnight with antibody coated beads. The beads were then washed and incubated with Detection Antibody for 1 h. Streptavidin-PE was then used as a reporter molecule. The plates were read and analyzed using Luminex 200 Milliplex Analyzer (MilliporeSigma, Burlington, MA).

Total glycogen synthase kinase 3 beta (GSK3β) and Ser9 phosphorylated GSK3β were measured from aforementioned newborn (P1) rat heart protein using GSK3β (Total/Phospho) Multispecies InstantOne ELISA Kit (Thermo Fisher Scientific, Waltham, MA) according to manufacturer's protocol. In short, 30 μg of protein lysate was added to the ELISA plate and incubated with Antibody Cocktail for 1 h. The wells were then washed with Wash Buffer and exposed to Detection Reagent. After 15 min, Stop Solution was added and the plate was read at 450 nm using Cytation 3 Spectrophotometer (BioTek, Winooski, VT).

### Extracellular Flux (XF) Analyses

A glycolytic stress test (GST) was used to compare basal and maximal glycolysis and reserve capacity of primary isolated neonatal (P1) rat cardiomyocytes (NRCM) from control and combination exposed offspring on Seahorse XF24 analyzer (Agilent Technologies, Palo Alto, CA). Methods and experimental validation were similar to that previously described (30). Briefly, newborn hearts were harvested in ice-cold HBSS. Atria were removed and ventricles were minced then digested with 2 mg/mL DNase I and 0.15% Trypsin. Digestion was stopped with bovine serum (BS) and cells were pelleted and transferred to 10:1 DMEM-1 (DMEM with 10% BS and 1% penicillin/streptomycin) with DNase I. Cells were incubated on an uncoated plastic dish for 1 h at 37°C in 5% CO_2_ to remove rapidly adhering fibroblasts. Live NRCM were then counted and plated at a seeding density of 40,000 cells/well to 0.1% gelatin coated V7-PS 24-well microplates in DMEM-2 (DMEM-1 with 100 μM bromodeoxyuridine) and incubated overnight (12 h) at 37°C with humidified 5% CO_2_. The following morning, media was changed to XF base media (Agilent Technologies), incubated without CO_2_ for 1 h, and then oxygen consumption rates (OCR) and extracellular acidification rates (ECAR) were measured at baseline and following injections to yield final well concentrations. Conditions were as follows:

- Port A: 10 mM D-(+)-glucose (Sigma G8644)
- Port B: 2 μM rotenone (Sigma R8875) + 4 μM antimycin A (Sigma A8674), respiratory complex inhibitors used to drive anaerobic glycolysis
- Port C: 200 μM monensin (Sigma M5273) + 0.25 μM carbonyl cyanide-4-(trifluoromethoxy) phenylhydrazone (FCCP, Sigma C2920) which uncouples aerobic respiration to ensure attainment of maximal anaerobic glycolysis

Outcome measures included: baseline glycolysis, glycolysis following glucose injection, and maximal glycolysis (defined as peak ECAR following rotenone/antimycin) because FCCP/monensin did not usually increase ECAR further. The proton production rate (PPR) was calculated as originally described by Mookerjee et al. to discern acidification from anaerobic glycolysis vs. mitochondrial respiration (30, 35).

### Histopathology

Formalin fixed and paraffin embedded P1 rat hearts (*n* = 13) were sectioned and stained with periodic acid-Schiff (PAS) stain to qualitatively compare glycogen stores. In brief, biventricular cross-sections were deparaffinized and rehydrated, stained with 0.5% periodic acid for 5 min then Schiff solution for 15 min and washed in running water 10 for minutes. Sections were then counterstained with hematoxylin, dehydrated, and mounted with a coverslip. Sections were digitally imaged with the Aperio VERSA 8 automated slide scanner and qualitatively analyzed using the Aperio Image Scope Software (Leica Biosystems Imaging, Buffalo Grove, IL).

### Mitochondrial Copy Number

Total DNA was extracted from P1 whole hearts (*n* = 6–7 males/group) via DNeasy Blood and Tissue Kit (Qiagen, Germantown, MD) following manufacturer's instructions. DNA integrity and concentrations were determined using Epoch spectrophotometer (BioTek, Winooski, VT). Relative mitochondrial DNA copy number was determined using qPCR with primers designed for mitochondrial control region (*D-loop*; Integrated DNA Technologies, Coralville, IA) and cytochrome-c oxidase I (*Mt-co1*; Thermo Fisher Scientific, Waltham, MA) as previously described (30, 36). All qPCR reactions were run in triplicate in ABsolute Blue QPCR Mix (Thermo Fisher Scientific) on a Stratagene Mx3000P thermocycler (Agilent Technologies, Santa Clara, CA). Gene-specific standard curves were calculated using rat mitochondrial DNA and MxPro software (Agilent Technologies, Santa Clara, CA) and used to calculate relative mitochondrial DNA copy number.

### Statistical Analysis

Statistical analysis of the microarray gene expression was performed using the unpaired unequal variance *t*-test (Welch test). The hierarchical clustering for groups and entities was performed using Euclidean distance metric and Ward's linkage algorithm. Statistical significance was set at 1.25 fold change (FC, > 1.25 and < −1.25) and *p* < 0.05. The microarray datasets generated and analyzed in this study are available in the NCBI Gene Expression Omnibus (GEO) database under accession number GSE150649. Results from PCR, protein expression, mitochondrial copy number and extracellular flux analyses were analyzed using student's *T*-test or Mann-Whitney to detect significant differences between P1 male controls vs. combination exposed offspring. These analyses were done using Prism software (GraphPad Software, La Jolla, CA) and statistical significance was set at *p* < 0.05.

## Results

### Maternal Diabetes and High-Fat Diet Alter the Transcriptome of the Newborn Offspring Heart

**Figure 2:**
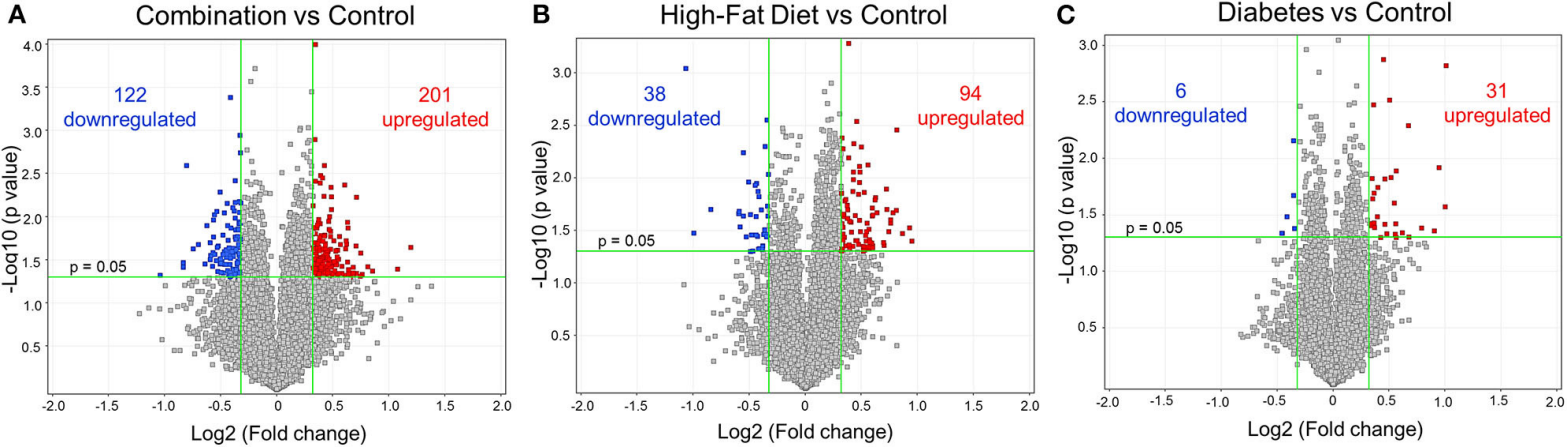
Differential gene expression of diabetes and high-fat (HF) diet exposed groups. Depicted are the differentially expressed genes (DEG) from all exposed groups visualized through volcano plots. In general, the different exposed groups compared to controls depicted different levels of DEGs. **(A)** Diabetes exposed group revealed the smallest changes with 6 downregulated and 31 upregulated DEGs when compared to controls. **(B)** HF diet group depicted 38 and 94 down and upregulated, respectively. **(C)** Combination of both diabetes and HF diet exposed the largest number of DEGs (323), with 122 downregulated and 201 upregulated. Significance threshold was set at > 1.25 and < −1.25 fold change (FC, green vertical lines) and *p* < 0.05 (green horizontal line).

Newborn rat hearts were examined by microarray analysis to determine transcriptome remodeling prompted by *in utero* exposures. Four groups were defined: controls (*n* = 7), diabetes exposed (*n* = 6), HF diet exposed (*n* = 5) and combination exposed (*n* = 5; Figure 1A). Principal component analysis and class prediction modeling (Figures 1B,C) confirmed reproducibility and consistency of transcriptional profiles. To characterize differentially expressed genes (DEG) in this study, exposure groups were compared in a pairwise fashion to controls as well as to other exposure groups (Figure 2, Figure S1, Tables S1–S6). Of the controls vs. exposure pairs analyzed, the greatest number of significant transcriptomic changes was detected for the combination vs. control comparison which included 323 DEGs, with 122 upregulated and 201 downregulated (Figure 2A). HF diet (Figure 2B) or diabetes (Figure 2C) alone vs. controls had fewer DEGs (132 and 37 DEGs, respectively) prompting our focus on categories of significant functional enrichment in the combination exposed group. Given that there are only 37 DEGs following diabetes exposure alone compared to 323 DEGs following combination exposure (diabetes and HF diet) highlights the importance of dietary fat intake during diabetic pregnancy. The impact of diet on diabetic pregnancy was further highlighted by analysis of non-control pair comparisons (Figure S1) which demonstrates 245 variable DEGs in the combination vs. diabetes exposure alone. To put this in perspective, the diabetes vs. HF diet comparison had only 60 DEGs. Moving forward, we prioritized the combination vs. control comparison for bioinformatic enrichment analysis as the synergistic effects of diabetes and HF diet on transcriptome remodeling, shown by the large number of significantly changed genes, is highly amenable to translational application.

### Cardiometabolic Pathways Are Prioritized in Expression Profiling of Offspring Hearts Exposed to Maternal High-Fat Diet and Diabetes

**Figure 3:**
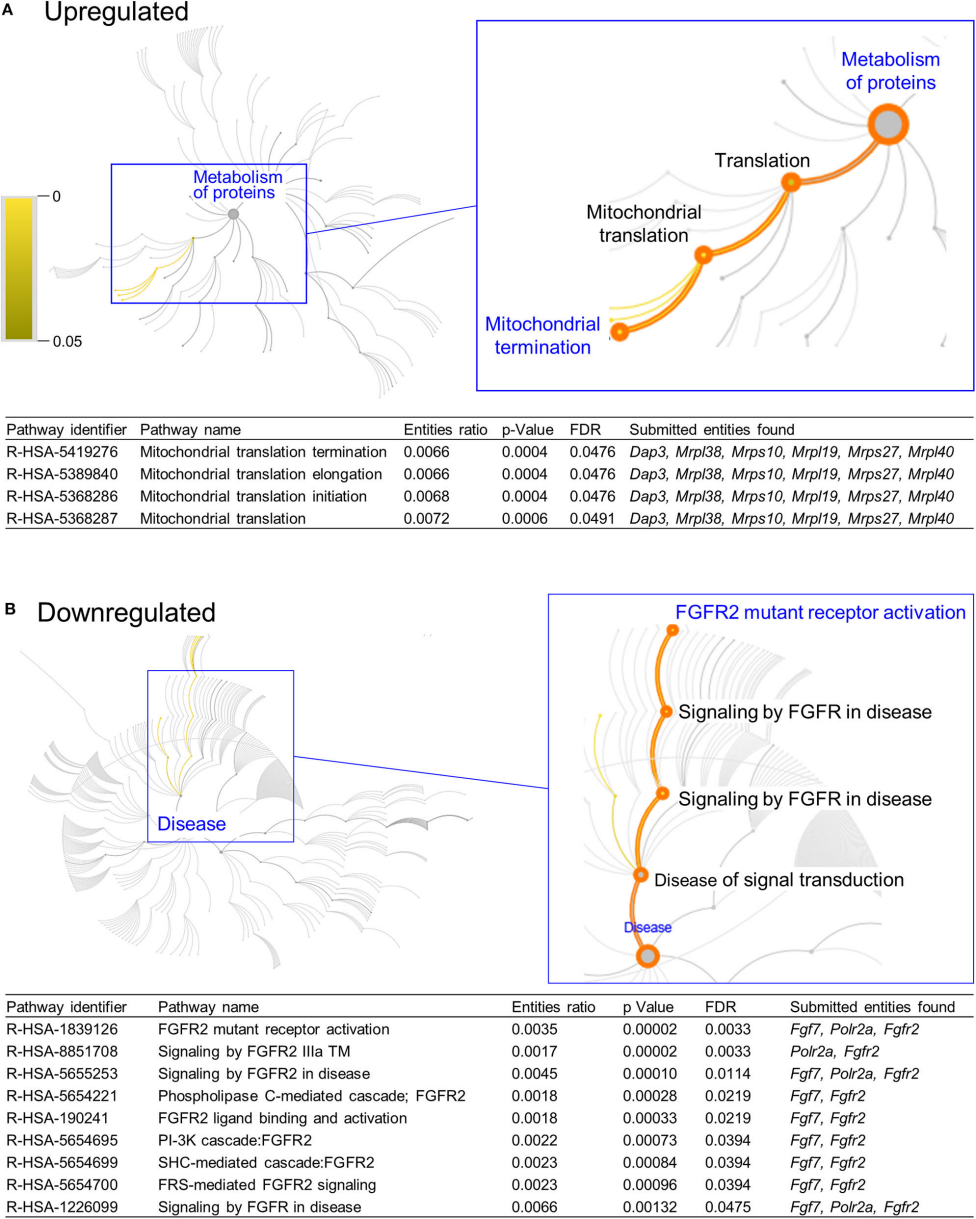
Functional pathways prioritized in differentially expressed genes from combination exposed group. Functional annotation and pathways analysis was done using Reactome pathway database in down and upregulated differentially expressed genes (DEGs) of combination exposed group when compared to control. **(A)** Analysis of the upregulated DEGs elicited four mitochondrial translation-related pathways, associated with *Dap3, Mrpl38, Mrps10, Mrpl19, Mrps27, Mrpl40*. **(B)** Downregulated list revealed nine functional pathways directly associated with fibroblast growth factor receptor 2 (*Fgfr2*).

DAVID functional annotation analysis of the upregulated gene list from the combination vs. control comparison identified “Mitochondrial translation” (elongation and termination), “Ribosomal protein,” and “Ribonucleoprotein” as functional category terms within the most prioritized cluster (Annotation Cluster 1, enrichment score: 2.371; Table 1). “Zinc finger, Cys2-His2 (C2H2)” was the prioritized functional term in the downregulated DEGs (Cluster 1, enrichment score: 0.982; Table 1). When we analyzed through DAVID the total up and downregulated genes (323 DEGs) we identified the same functional category terms (“Mitochondrial translation,” “Ribosomal protein,” and “Ribonucleoprotein”) within the first cluster (enrichment score: 1.718; Table S7). Further functional pathway enrichment analysis of up and downregulated gene lists using the Reactome Pathway Database prioritized “Metabolism of proteins” within the upregulated list of genes from the combination exposed vs. control comparison, with identification of “Mitochondrial translation,” “Mitochondrial translation termination,” “Mitochondrial translation elongation,” and “Mitochondrial translation initiation” terms (Figure 3A). Genes associated with this prioritized functional class included the following mitoribosomal genes: mitochondrial ribosomal protein L38 (*Mrpl38*), mitochondrial ribosomal protein S10 (*Mrps10*), *Mrpl19, Mrps27*, mitochondrial ribosomal protein L40 (*Mrpl40*), in addition to *Dap3* (also known as *Mrps29*), a regulator of mitochondrial dynamism and cell fate (37–39). Moreover, analysis of the downregulated gene set prioritized “Disease of signal transduction” (Figure 3B). Functional terms identified with this pathway included “Fibroblast growth factor receptor (FGFR) signaling” associated terms, including “Phosphoinositide 3-kinase (PI3K) cascade,” with the following genes enriched: fibroblast growth factor 7 (*Fgf7*), *Fgfr2*, and RNA polymerase II subunit A (*Polr2a*; Figure 3B). When the total DEGs were analyzed it reinforced the prioritization of FGFR and PI3K signaling pathways (Table S8).

**Table 1:** Functional pathway enrichment analysis of significantly upregulated and downregulated genes in Combination exposed vs Control group.

		**Category**	**Term**	**Count**	***p*-value**	**FE**	**Bonferroni**	**Benjamini**	**FDR**
**Upregulated**									
	Annotation Cluster 1	REACTOME_PATHWAY	R-RNO-5389840: Mitochondrial translation elongation	6	<0.001*	8.89	0.05	0.05	0.54
	(2.371)	REACTOME_PATHWAY	R-RNO-5419276: Mitochondrial translation termination	6	<0.001*	8.66	0.05	0.03	0.61
		UP_KEYWORDS	Ribosomal protein	5	0.021*	4.70	0.96	0.36	22.60
		UP_KEYWORDS	Ribonucleoprotein	5	0.057	3.42	1.00	0.61	50.38
	Annotation Cluster 2	GOTERM_CC_DIRECT	GO:0016021, integral component of membrane	17	0.999	0.52	1.00	1.00	100.00
	(2.182)	UP_KEYWORDS	Transmembrane helix	19	0.999	0.53	1.00	1.00	100.00
		UP_KEYWORDS	Transmembrane	19	0.999	0.53	1.00	1.00	100.00
		UP_KEYWORDS	Membrane	24	0.999	0.56	1.00	1.00	100.00
**Downregulated**									
	Annotation Cluster 1	INTERPRO	IPR015880:Zinc finger, C2H2-like	5	0.076	3.08	1.00	1.00	64.49
	(0.982)	INTERPRO	IPR007087:Zinc finger, C2H2	5	0.093	2.86	1.00	1.00	71.40
		SMART	SM00355:ZnF_C2H2	5	0.160	2.31	1.00	1.00	83.54
	Annotation Cluster 2	UP_KEYWORDS	Membrane	30	0.195	1.18	1.00	1.00	91.64
	(0.633)	UP_KEYWORDS	Transmembrane helix	25	0.251	1.17	1.00	1.00	96.35
		UP_KEYWORDS	Transmembrane	25	0.259	1.17	1.00	0.99	96.78
	Annotation Cluster 3	INTERPRO	IPR007110:Immunoglobulin-like domain	4	0.251	2.27	1.00	1.00	97.48
	(0.435)	INTERPRO	IPR003599:Immunoglobulin subtype	3	0.378	2.26	1.00	1.00	99.77
		SMART	SM00409:IG	3	0.522	1.69	1.00	1.00	99.95
	Annotation Cluster 4	INTERPRO	IPR000719:Protein kinase, catalytic domain	4	0.279	2.14	1.00	1.00	98.47
	(0.403)	INTERPRO	IPR011009:Protein kinase-like domain	4	0.322	1.98	1.00	1.00	99.30
		INTERPRO	IPR008271:Serine/threonine-protein kinase, active site	3	0.332	2.50	1.00	1.00	99.42
		UP_KEYWORDS	Kinase	4	0.393	1.75	1.00	0.99	99.68
		INTERPRO	IPR017441:Protein kinase, ATP binding site	3	0.422	2.06	1.00	1.00	99.91
		SMART	SM00220:S_TKc	3	0.551	1.61	1.00	1.00	99.97
		GOTERM_BP_DIRECT	GO:0006468~protein phosphorylation	3	0.551	1.61	1.00	1.00	100.00
	Annotation Cluster 5	GOTERM_MF_DIRECT	GO:0003682~chromatin binding	4	0.208	2.50	1.00	1.00	94.18
	(0.273)	UP_KEYWORDS	Methylation	3	0.520	1.71	1.00	0.99	99.98
		UP_KEYWORDS	Ubl conjugation	4	0.551	1.37	1.00	0.99	99.99
		UP_KEYWORDS	DNA-binding	3	0.832	0.93	1.00	1.00	100.00
	Annotation Cluster 6	INTERPRO	IPR000276:G protein-coupled receptor, rhodopsin-like	3	0.978	0.54	1.00	1.00	100.00
	(0.002)	UP_KEYWORDS	G-protein coupled receptor	3	0.985	0.50	1.00	1.00	100.00
		INTERPRO	IPR017452:GPCR, rhodopsin-like, 7TM	3	0.985	0.50	1.00	1.00	100.00
		UP_KEYWORDS	Transducer	3	0.988	0.48	1.00	1.00	100.00

The enrichment analysis on differentially exposed genes (DEG) was performed with DAVID Functional annotation software using a high stringency. Depicted here for the upregulated DEGs are two distinctive annotation clusters (enrichment score shown in parenthesis) and six clusters for the downregulated list, with their respective functional terms, number of genes associated with each term (count), statistical significance (*p < 0.05), fold enrichment (FE) and false discovery rate (FDR).

### Gene Regulatory Network Disruption in Developing Offspring Identifies a Functional Hub Regulating Metabolism and Cell Fate

**Figure 4:**
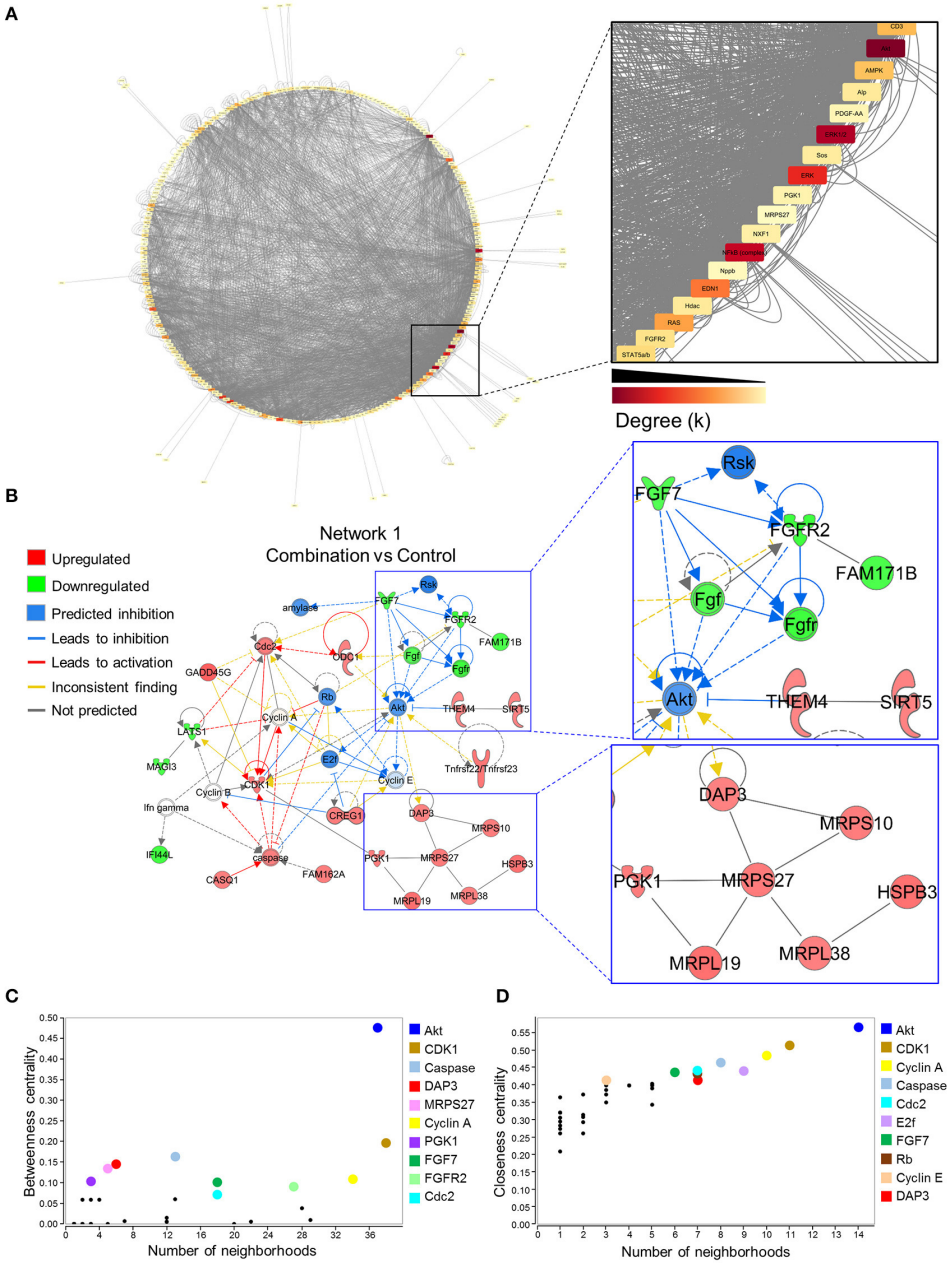
*Akt* is a critical hub within the network of transcriptome remodeling that characterizes hearts of combination exposed offspring. Interactions of merged functional networks, constructed in IPA from statistically significant genes in combination exposed group, were uploaded into Cytoscape for network analysis. **(A)** Circular network layout depicting node interactions and colored by degree. Magnification of region with highest edge density (dark gray) showing *Akt* as the network node with the highest degree, followed by *Erk1/2, Erk, NfkB* (complex), *Edn1, Ras, Ampk, Cd3, Alp, Fgfr2, Stat5a/b, Hdac, Pdgf-AA, Sos, Pgk1, Nxf1, Nppb* and *Mrps27* (highest to lowest degree, respectively). **(B)** IPA Molecule Predictor Analysis of the highest priority network (Network 1, Score 39, top functions: cell cycle, protein synthesis, hair and skin development/function) predicted inhibition (blue) of *Akt* in the combination exposed newborn heart by net expression of differentially expressed genes. Right: Magnification of *Fgfr2* (downregulated in green) and *Mrps27* (upregulated in red) hubs and immediate subnetwork neighborhood. **(C,D)** Graph theory analysis of Network 1 (Combination vs. control) revealed *Akt* as a network hub with the highest betweenness and closeness centrality metrics, indicative of its importance to overall network structure and communication. Among the top 10 nodes prioritized include: *Cdk1, Caspase, Dap3, Mrps27, Cyclin A, Pgk1, Fgf7, Fgfr2, Cdc2, E2f* and *Rb*. IPA, Ingenuity Pathways Analysis; Degree (*k*) = neighborhood connectivity distribution.

To investigate gene networks underlying transcriptome remodeling of the combination exposed offspring hearts, IPA was used to map known molecular interactions among all DEGs identified in the present study. All subnetworks that capture gene interactions within the present dataset were integrated into an inclusive, overall network displayed in a circular layout (Figure 4A). Increased edge density (gray lines) in the lower right quadrant identifies hubs, or highly connected nodes within this network. Focusing on the most prioritized subnetwork with an enrichment score of 39 revealed over representation of “Cell Cycle,” “Protein Synthesis,” and “Hair and Skin Development/Function” categories (Figure 4B). Predictive network modeling applied to this subnetwork identified potential Serine/Threonine-protein kinase B (*Akt*) inhibition given the net synergistic gene expression changes in the present dataset (Figure 4B). In addition, this subnetwork integrated up and downregulated genes that represented mitoribosomal signaling and FGF cascades (Figure 4B). Topology analysis of this prioritized subnetwork identified *Akt* as a functional hub with the highest betweenness centrality score (Figure 4C). To identify network hubs regulating informational flow, *Akt*, Cyclin dependent kinase 1 (*Cdk1*), and Cyclin A were identified as molecules with the highest closeness centrality measures in the present network (Figure 4D). Together, these data identified *Akt* as a strong candidate pathway involved in reprogramming of diabetes and HF diet exposed cardiac tissue.

### Expression Dysregulation of Mitochondrial Specific Genes

**Figure 5:**
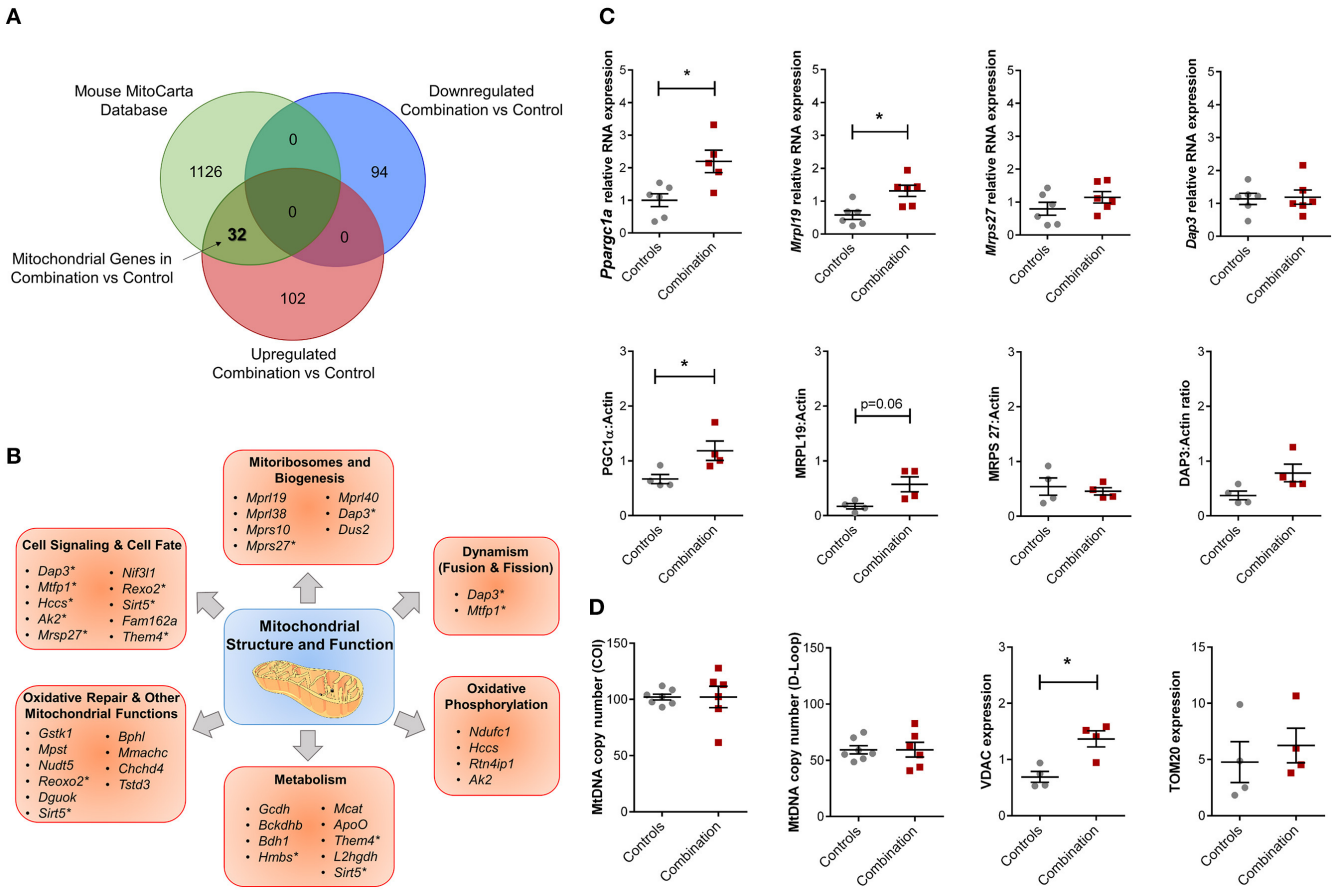
Combination exposed offspring hearts enriches discrete classes of mitochondrial genes. Genes that were duplicated or non-annotated were excluded, resulting in 228 differentially expressed genes (DEG) with 94 down and 134 upregulated in combination exposed hearts when compared to controls. **(A)** The 228 DEGs were plotted in a Venn diagram against 1,158 mitochondrial associated genes (MitoCarta Database, Mouse) and depicted 32 upregulated mitochondrial genes (Table S9) with none downregulated in the combination exposed offspring hearts. **(B)** Mitochondrial functions associated with the 32 upregulated mitochondrial genes were: cell signaling and cell fate, mitochondrial biogenesis, dynamism, oxidative phosphorylation, metabolism, oxidative repair and other mitochondrial functions. *Genes with multiple functions; shown in more than one category. **(C)** RNA expression of *Ppargc1a* and *Mrpl19* were significantly upregulated with fold change (FC) of 2.24 and 2.28, respectively (*p* < 0.05) in combination exposed newborn hearts (red squares) compared to controls (gray circles; *n* = 6/group). We showed no significant change in RNA expression of *Mrps27* (FC = 1.09, *p* = 0.6) or *Dap3* (FC = 1.10, *p* = 0.75). Consistent with the RNA expression, protein expression of PGC1α was significantly increased in newborn hearts exposed to diabetes and HF diet when compared to controls (*n* = 4/group, *p* < 0.05). We observed a non-significant upregulation trend for MRPL19 and DAP3 protein expressions with no change in MRPS27. **(D)** Relative mtDNA copy number was not different by mtDNA expression of either cytochrome-c oxidase I (*COI*) or mitochondrial control region (*D-loop*). However, combination exposed hearts had higher protein expression of Voltage dependent anion channel 1 (VDAC) and a trend toward higher Translocase of outer mitochondrial membrane 20 (TOM20).

The overall bioinformatic enrichment data alongside previously published changes in bioenergetics and metabolic functions (30) prompted a focused query on genes regulating mitochondrial function. Intersection of prioritized up and downregulated transcriptional profiles in our study with the independently curated MitoCarta 2.0 database (40) that includes 1,158 mitochondrial associated genes, revealed 32 upregulated mitochondrial genes in the combination vs. control comparison with no observation of common downregulated genes (Figure 5A; Table S9). These 32 genes could be further sub-classified into multiple categories of mitochondrial structure and function, including “Cell Signaling and Cell Fate,” “Mitoribosomes and Biogenesis,” “Dynamism,” “Oxidative Phosphorylation,” “Metabolism,” “Oxidative Repair,” and other mitochondrial functions (Figure 5B). Candidate genes for validation included *Ppargc1a* (encoded protein also known as PGC1α, a master regulator of mitochondrial biogenesis), as well as genes that encode mitoribosomal proteins *Mrpl19, Mrps27* and *Dap3* (Figure 5C). Of these, validation by qPCR confirmed that *Ppargc1a* and *Mrpl19* mRNA expression were significantly higher in the combination exposed P1 males when compared to controls (FC 2.24 and 2.28, respectively, *p* < 0.05, *n* = 6/group; Figure 5C, top row). *Mrps27* and *Dap3* mRNA expression was not different between the two groups (Figure 5C, top row). Immunoblotting to assess relative expression of protein products for each of these genes confirmed higher expression of PGC1α (*n* = 4/group, *p* < 0.05), non-significant trends toward higher expression for MRPL19 and DAP3, and no observed change for MRPS27 (Figure 5C, bottom row). In line with this, we previously reported a higher mitochondrial DNA copy number in HF diet and combination exposed offspring hearts (30). In this study, we did not detect a higher mitochondrial DNA copy number by PCR (Figure 5D). However, combination exposed hearts had higher expression of outer mitochondrial membrane proteins TOMM20 or VDAC with significant increases observed in the latter compared to controls.

### Downregulated FGFR2/PI3K/AKT Activation Underlies Metabolic Switching From Glycolysis to Gluconeogenesis in the Combination Exposed Offspring Hearts

**Figure 6:**
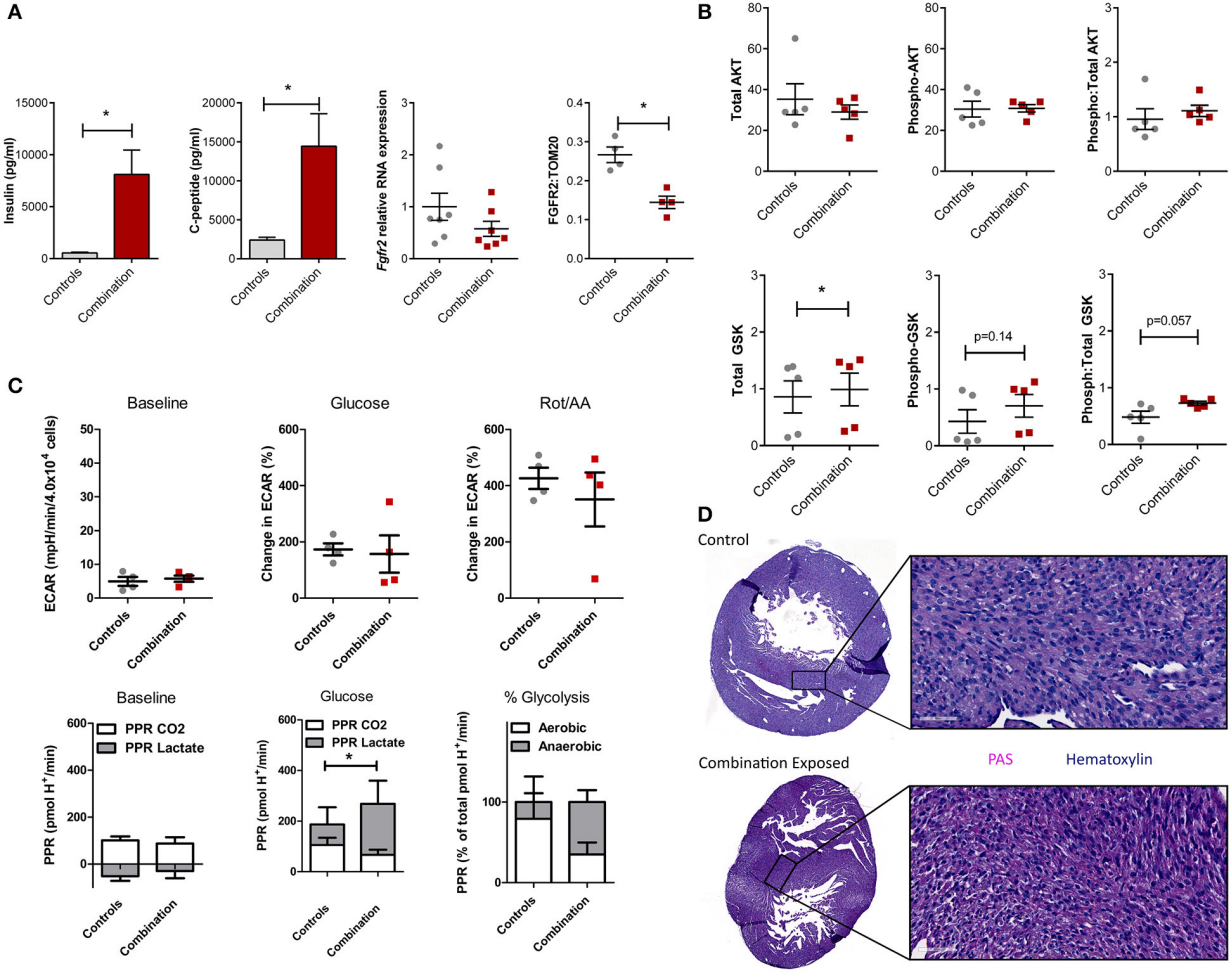
Evidence of insulin resistance in diabetes and high fat diet exposed offspring hearts. Chronic exposure to insulin and other growth hormones can cause insulin resistance through downregulation of growth factor receptors and impaired downstream activation of the PI3K/AKT pathway which shifts metabolism from glycolysis to gluconeogenesis/glycogen accumulation. **(A)** Combination exposed newborn offspring (*n* = 10/group) had significantly higher circulating insulin and c-peptide levels. Consistent with transcriptome analyses, combination exposed newborn male hearts, had a trend toward lower RNA expression relative to *B2m* (*p* = 0.13, *n* = 7/group) and lower protein expression (*p* < 0.05, n = 4/group) of FGFR2. **(B)** Total and phosphorylated AKT (*n* = 5/group) was not different, but GSK3β was higher (*n* = 5/group) and there was a trend toward more phosphorylated (active) and ratio of phosphorylated:total GSK3β (*p* = 0.14 and *p* = 0.057, respectively) in combination exposed, male hearts. **(C)** Primary isolated newborn rat cardiomyocytes (NRCM) from combination exposed male offspring had no significant difference in baseline extracellular acidification rate (ECAR), glucose or rotenone/antimycin (Rot/AA) stimulated glycolysis (glycolytic capacity) by XF analyses (top row). The proton production rate (PPR) was calculated to estimate aerobic (PPR from CO_2_) and anaerobic (PPR from lactate) glycolysis. At baseline, there was no difference, but aerobic glycolysis was significantly lower in combination exposed NRCM following glucose injection. Combination exposed NRCM had only 34% aerobic glycolysis vs. 79% aerobic glycolysis following glucose in controls. This suggests maternal diabetes and high fat diet exposure impairs aerobic glycolytic capacity. *p < 0.05, *n* = NRCM pooled from 3 to 4 pups/litter, 4 litters/group. **(D)** Periodic Acid Schiff (PAS) staining demonstrates more glycogen deposition in combination exposed hearts, which suggests a chronic *in utero* switch from glucose utilization to storage occurred during development.

We previously demonstrated that combination exposed offspring (P1) are exposed to high levels of circulating maternal glucose and lipids which incite insulin and C-peptide overproduction in the offspring (30–34). This triad of maternal hyperglycemia and hyperlipidemia and fetal hyperinsulinemia causes insulin resistance with attendant downregulation of growth hormone receptors and less PI3K/AKT activation to drive a glycolysis-to-gluconeogenesis switch (33, 41–43). Informed by the observed disruption in the gene regulatory network in the present study, circulating insulin, c-peptide, and the cardiac *Fgfr2* gene and FGFR2 protein expression were assessed. Insulin and c-peptide levels were significantly higher in combination exposed offspring compared to controls (539 ± 77 vs. 8,103 ± 2,337 pg/mL; *p* = 0.005 and 2,391 ± 367 vs. 14,449 ± 4,175 pg/mL, respectively, *p* < 0.01; *n* = 10/group, Figure 6A). *Fgfr2* mRNA levels showed a modest decreasing trend in expression (FC−1.93, *p* = 0.16), with significantly lower FGFR2 protein expression in combination exposed hearts when compared to controls (*p* < 0.05; Figure 6A). Examination of total as well as activated (phosphorylated) AKT expression showed no significant differences between control and combination exposed hearts (Figure 6B), however, AKT phosphorylation changes rapidly in response to fuels, thus levels would be expected to normalize after birth as glucose and insulin levels rapidly decline. GSK3β, is regulated by AKT and when activated by phosphorylation, facilitates gluconeogenesis in multiple tissues including the heart (44, 45). In the present study, total GSK3β was moderately increased in combination exposed offspring hearts compared to controls (0.86 ± 0.28 vs. 0.99 ± 0.29, *p* < 0.05; Figure 6B, bottom row) with a trend toward higher activation as demonstrated by phosphorylated (0.43 ± 0.21 vs. 0.70 ± 0.20, *p* = 0.14) and a ratio of phosphorylated:total GSK3β (0.48 ± 0.11 vs. 0.73 ± 0.03, *p* = 0.057) (Figure 6B, bottom row). Again, although significance was not reached, cell signaling through phosphorylation is rapid and so downstream metabolism and glycogen storage within the heart is a more accurate measure of longstanding activation due to *in utero* exposures.

In our previous studies, primary isolated NRCM from diabetes exposed offspring had impaired respiratory and glycolytic capacity (30). The present study had a smaller male cohort in which cardiomyocytes from combination exposed rat offspring exhibited a similar glycolytic capacity as controls (Figure 6C, top row). However, PPR calculations showed combination exposed NRCM have impaired aerobic glycolytic capacity and metabolize glucose through anaerobic glycolysis instead. Additionally, PPR due to CO_2_ production in combination exposed cardiomyocytes did not rise with glycolytic stimulation, suggesting impaired glucose uptake which is the rate limiting step of glycolysis (Figure 6C, bottom row). Moreover, qualitative histological examination by PAS staining revealed increased glycogen deposition in combination exposed offspring hearts (Figure 6D). Taken together, combination exposed P1 male hearts provide evidence of insulin resistance with a metabolic predilection to switch from glycolysis to gluconeogenesis.

## Discussion

**Figure 7:**
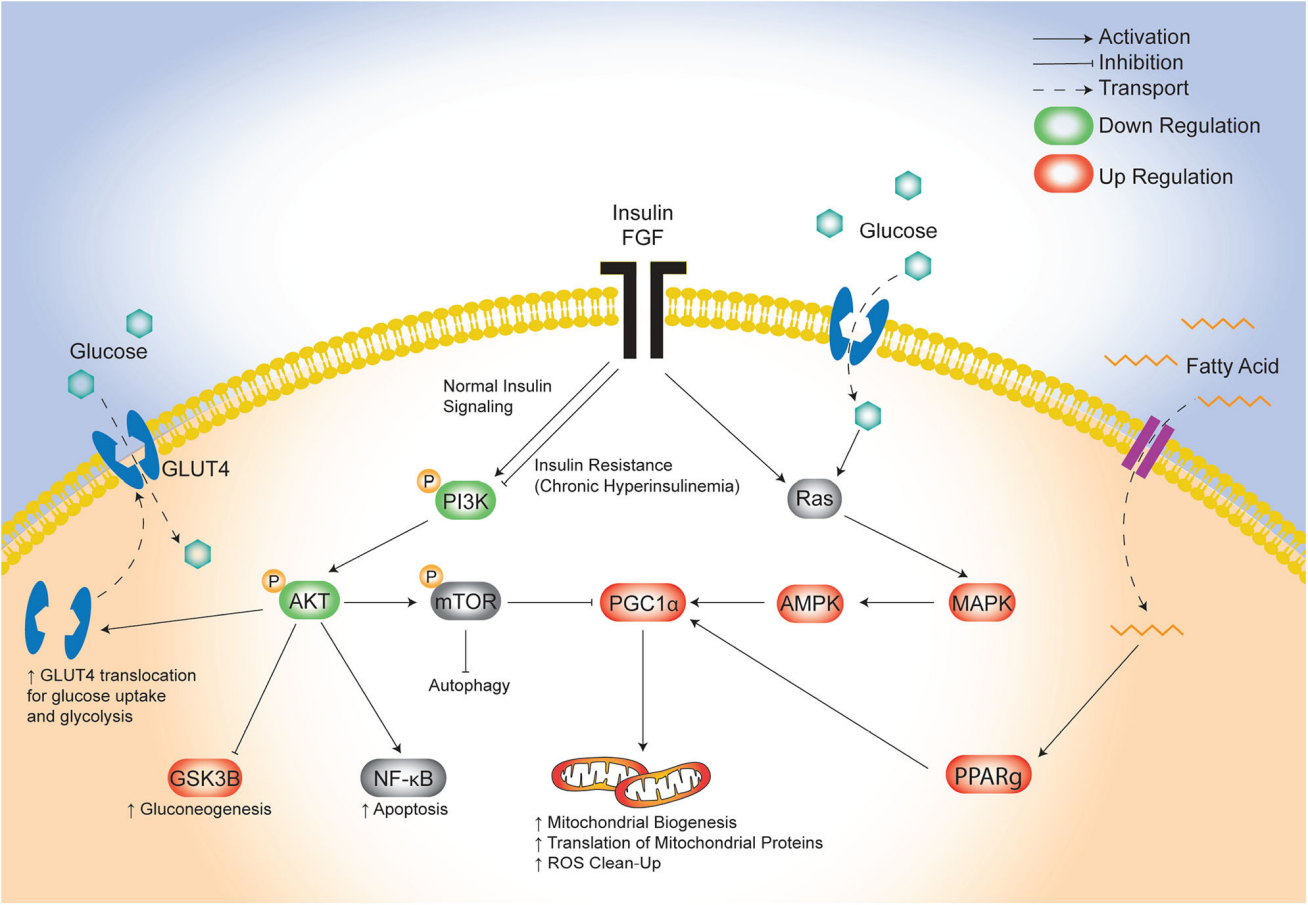
Proposed model of maternal combination exposed diet effects on cardiac network signaling in offspring. This illustrative schematic depicts the metabolic pathways in which the triad of maternal hyperglycemia, hyperlipidemia, and fetal hyperinsulinemia leads to an increased mitochondrial biogenesis, autophagy, and a shift from glycolysis to gluconeogenesis. This occurs via a downregulated PI3K/AKT pathway and increased PGC1α expression amongst uninhibited MAPK activity. AKT, protein kinase B; AMPK, 5′ adenosine monophosphate-activated protein kinase; FGF, fibroblast growth factor; GLUT4, glucose transporter type 4; GSK3β, glycogen synthase kinase 3 beta; MAPK, mitogen-activated protein kinase; mTOR, mammalian target of rapamycin; NF-kB, nuclear factor kappa-light-chain-enhancer of activated B cells; PGC1α, PPARG coactivator 1 alpha; PPARg, peroxisome proliferator activated receptor gamma; Ras, proto-oncogene protein p21.

The lifetime risk of CVD remains close to 50% for men and 38% for women making it the leading cause of morbidity and mortality in developed countries (46). There is an urgent need to identify targetable triggers of CVD in high-risk populations so that the earliest risk-assessment and personalized interventions can be implemented to decrease this growing burden of disease over time. Infants born to diabetic mothers (IDM) are a high-risk population with 36% having pathologic ventricular hypertrophy at birth (47) and as young adults having ~30% greater risk of early onset CVD (21). Developmental consequences arise following exposure to excess circulating fuels during critical windows of fetal development (48), specifically for diabetic pregnancy this includes maternal hyperglycemia, hyperlipidemia and fetal hyperinsulinemia. Similarly, this triad also causes diabetic cardiomyopathy in adults which has overlapping clinical manifestations with cardiomyopathy found in IDM (11, 49–52). Unfortunately, even when maternal hyperglycemia is well-controlled during pregnancy, the risk of heart disease in IDM persists (17, 18, 20, 53); this implicates additional targetable triggers including lipids. We previously used a rat model to show that a maternal HF diet compounded the effects of late-gestation diabetes by exposing the offspring not only to hyperglycemia, but also to hyperlipidemia which exacerbated fetal insulin production, placental lipotoxicity, cardiac dysfunction, and perinatal mortality (30, 34). The present study used cardiac-specific transcriptomic profiling to identify potential underlying pathogenic mechanisms. The key finding was identification of distinct, yet overlapping disruptions in glucose-, insulin-, and lipid-modulated networks that influence cardiac development and disease (Figure 7). Specifically, the combination of maternal diabetes and HF diet caused down regulation of a well-known glucose and insulin signaling pathway, FGRF2/PI3K/AKT, as well as a concomitant upregulation of fatty acid regulated PGC1α (54). Both pathways serve as central hubs that sense nutrient supply to influence cellular metabolism as well as growth, development and aging (42, 43, 55, 56).

Cellular metabolism is of critical importance for the heart which must efficiently shift from one metabolic pathway to another in order to maintain efficient contractily under variable energetic demands (aerobic, anaerobic, resting, exercise, starvation, etc). A small decrease in metabolic efficiency can have profound impacts on cardiac function leading to hypertrophy, stiffness, diastolic and systolic dysfunction (57, 58). Transcriptomic profiling suggests combination exposed offspring hearts have relative insulin resistance with downregulation of growth factor receptors, likely due to *in utero* fetal hyperinsulinemia which occurs in response to maternal hyperglycemia. Insulin and other growth factors normally activate PI3K to generate 3,4,5 phosphatidylinositol (PIP3) to recruit AKT to the plasma membrane for activation by phosphorylation on the threonine 308 residue (T308) and serine 473 (S473) by phospoinositide-dependent kinase 1 (PDK1) (59). In the present study, combination exposed P1 offspring had higher insulin and c-peptide levels with concomitant down regulation of FGFR2, but we did not find lower total or S473 phosphorylation of AKT. We speculate that after birth AKT may have been dephosphorylated in the length of time between offspring removal from the *in utero* diabetic environment and cardiac tissue harvest. Indeed, offspring's glucose and insulin levels fall rapidly after birth and kinetic studies show peak AKT phosphorylation and activity occurs 15 min after insulin receptor activation, and dephosphorylation occurs within 30 min (59) and hearts were typically harvested around 8–12 h of age. Additionally, AKT activation can be modulated by phosphorylation at unmeasured sites (59). So, while we did not find direct evidence of impaired PI3K/AKT quantity or phosphorylation, we found residual downstream metabolic impairment as evidence this occurred *in utero*. In line with this, we previously demonstrated that isolated NRCM from combination exposed offspring had lower glycolytic capacity (30). A limitation of this study is that we did not replicate the same severity of glycolytic impairment, but found impaired aerobic glycolysis, higher GSK3β, and more cardiac glycogen deposition, consistent with a chronic shift from glycolysis to gluconeogenesis. This switch carries significant bioenergetic consequences as the newborn heart relies primarily on aerobic glycolysis for ATP production (60, 61). Our findings demonstrate that diabetic pregnancy, especially combined with hyperlipidemia precipitates cardiac “insulin resistance” in the developing fetal heart. The transitory nature of the pathway may explain why IDM have diastolic and systolic dysfunction that begins *in utero* around the time fetal pancreatic endocrine function begins, but then improves after birth (7, 62–64). Authors propose that when the fetus is no longer exposed to the triad of hyperglycemia, hyperlipidemia, and hyperinsulinemia PI3K/AKT activation is no longer suppressed. This postnatal correction, alongside the physiologic transition from glycolysis to fatty acid oxidation during cardiac maturation (60), could explain why cardiomyopathy in IDM improves after birth. However, this does not explain how infants born to diabetic or obese mothers develop early onset CVD in adulthood (6, 21, 27, 65, 66). We propose that this is related to either lasting effects of disturbed cardiogenesis or advanced aging from the up-regulated PPAR pathway.

Both of the pathways we uncovered by transcriptomic profiling are well-known modulators of stem cell fate, which could influence cardiogenesis and aging as it relates to long-term cardiovascular health (56, 67). The current study adds additional evidence to our previous work, that maternal diabetes and HF diet affect cardiac health through mitochondrial mediated influences on cell fate and aging processes (30, 31, 68, 69). Our lab has shown that primary isolated cardiomyocytes from combination exposed offspring have poorly charged, adynamic, fragmented mitochondria which can produce ROS and incite mitochondrial mediated cell death (30, 31). Developing cardiomyocytes normally have highly dynamic mitochondria; after birth they undergo rapid mitochondrial biogenesis and ultrastructural differentiation into distinct sub-populations to meet changing metabolic demands (60, 70–72). Mitochondrial biogenesis requires complex coordination of nuclear and mitochondrial transcription, translation and protein assembly that is regulated by transcriptional regulators including PGC1α (73). PGC1α is a positive regulator of mitochondrial biogenesis, oxidative phosphorylation, gluconeogenesis, and ROS-detoxification (73), and is regulated at both transcriptional and post-translational levels through similar yet competing PI3K/AKT and MAPK pathways (Figure 7) (74). Oxygen, nutrients, and ROS in the heart signal for the insulin-sensitive PI3K/AKT pathway to reduce PGC1α activity; conversely, they signal for MAPK and glucose-responsive AMPK phosphorylation to increase PGC1α transcriptional activity (74). In this study, newborn offspring exposed to maternal hyperglycemia and hyperlipidemia and fetal hyperinsulinemia demonstrated down-regulated PI3K/AKT and increased PGC1α expression. Together, these transciptomic changes would be expected to increase translation (but not transcription) of mitochondrial proteins, mitochondrial biogenesis, and gluconeogenesis while decreasing glycolysis (43). This shift in metabolic machinery is the resut of coordinated increases in expression of nuclear and mitochondrial encoded genes needed for mitochondrial biogenesis.

It is critical to point out that while the vast majority of proteins regulating mitochondrial function are encoded by the nuclear genome and transported to the mitochondria, respiratory complex assembly occurs within the mitochondria guided by mitochondrial DNA (mtDNA) transcription and translation (75). Located within mitochondrial nucleoids, mtDNA coding for the respiratory complex components are translated into proteins by mitochondrial ribosomes or mitoribosomes under the influence of P32 (complement component 1, q subcomponent binding protein or C1qBP; also called gC1qR or HABP1) (75). All 82 proteins that make up the mitoribosome are encoded by the nuclear genome and transported into mitochondria for assembly (76). In this study, cardiac transcriptome analyses identified upregulation of P32 mitoribosomal proteins in combination exposed offspring. Specifically upregulated were *Mrpl19, Mrpl38, Mrps10, Mrps27*, and *Dap3*, mitoribosomal proteins influenced by P32, an essential RNA-binding factor in mitochondrial translation indispensable for oxidative phosphorylation and embryonic development (77). Additionally, *Mrpl40*, a mitoribosomal protein associated with P32, was upregulated in the combination exposed cardiac transcriptome. MRPL40 is located adjacent to the ribosomal polypeptide exit site and is important for synthesis of mitochondrially encoded proteins and their subsequent assembly into oxidative phosphorylation complexes (78).

DAP3 and P32 play additional roles in mitochondrial function and cell fate (37–39, 77). We previously detailed a sex-specific role for DAP3 in impaired dynamism and mitochondrial quality control in combination exposed male offspring (31). As a chaperone for mitochondrial protein synthesis, P32 does not affect the amount of mtDNA or mRNA but rather mitochondrial protein homeostasis, respiration (77), and cell survival (79). While we have previously demonstrated a higher mtDNA copy number and oxidative stress in diet-exposed offspring, the aforementioned anticipated post-transcriptional changes may explain why combination exposed hearts in this study did not have a significantly higher mtDNA copy number but expressed more VDAC, a mitochondrial outer membrane protein that is often used as a surrogate marker of mitochondrial quantity.

Alternatively, expression of genes influencing mitochondrial biogenesis may be upregulated as a compensatory mechanism to increase turnover of dysfunctional mitochondria found in combination exposed NRCM (30, 31). Transcriptomic evidence from this study demonstrates a compensatory shift to increase oxidative phosphorylation including up regulation of *Ndufc1, Hccs*, and *Rtn4ip1* which encode proteins necessary for orchestrated assembly of respiratory complexes or their cofactors. Specifically, NDUFC1 is a subunit in mitochondrial Complex I and HCCS is critical for cytochrome c biogenesis which functions in electron transport from Complex III to Complex IV (80, 81). HCCS is also is a key regulator of cell fate and cardiomyocyte differentiation. Adenylate kinase 2 (*Adk2*) was also upregulated in combination exposed offspring. Its encoded protein, AK2, is part of the adenylate kinase shuttling pathway, a central metabolic hub which enables the transfer of phosphoryl groups to interconvert 2ADP → ATP + AMP. This reaction is not only critical for ATP generation but is a sensitive rheostat of cellular energy that regulates the balance of energy storage and utilization through multiple downstream AMP signaling pathways. Specifically, AK2 is highly expressed in cardiac tissue and localized to the mitochondrial intermembrane space where it facilitates conversion of AMP to ADP shuttling. AK2 has an extremely high affinity for AMP which assures that AMP reaching cardiac mitochondria is converted to ADP and channeled into oxidative phosphorylation. During cardiometabolic stress, mitochondrial AK2 activity is increased in response to increased energy demand and the necessity to maintain the cellular adenine nucleotide pool; moreover higher AK2 is often associated with cardiac hypertrophy (82). Whether or not AK2 is higher in combination than control offspring hearts because mitochondrial number is higher, probably in response to impaired bioenergetics, is unknown.

In addition to these network changes, individual DEGs were detected when comparing diabetes or HF diet to controls. In the “Diabetes” vs. “Control” analysis, all genes that changed by 1.5 fold or more were upregulated. Specifically, we identified higher expression of alpha hemoglobin stabilizing protein (*Ahsp*) and Kell metallo-endopeptidase (*Kel)* which encode proteins typically highly expressed in red blood cells. Neonatal polycythemia is a common complication associated with maternal diabetes, and it is possible that higher expression of *Ahsp* and *Kel* are due to a higher number of residual red cells in the myocardial vasculature within our samples. However, *Ahsp* and *Kel* are found in other tissue such as endothelial cells, including coronary arteries where they reportedly regulate vasoconstriction through the oxygen sensing eNOS and endothelin-3 activation, respectively (83, 84). It is plausible these expression differences convey commonly reported vascular manifestations in offspring of diabetic mothers (33, 85). Another upregulated gene is *Car1* which encodes carbonic anhydrase I (CA-1). While best known for their role in proximal renal tubules, carbonic anhydrases are ubiquitous proteins that regulate pH balance and dependent cellular processes by catalyzing the reversible conversion of carbon dioxide to bicarbonate. In the heart, CA-1 is expressed in both endothelial cells and myocardium and upregulation activates the Na^+^/H^+^ exchange and calcium influx; when over activated CA-1 causes cardiac hypertrophy (86). Moreover, adults with diabetic cardiomyopathy have overexpression of myocardial CA-1, lower capillary density, and worse pathological remodeling after ischemia-reperfusion injury than non-diabetics (87). *In vitro* studies confirm that hyperglycemia increases expression of CA-1 in endothelial cells to disrupt vasculogenesis (87). *Frzb* encodes frizzled-related protein, a modulator of the Wnt/β-catenin signaling pathway which plays a central role in cardiac development as well as cardiac remodeling and aging (88). *Frzb* has been identified as a core gene biomarker in clinical reports of adults with diabetic cardiomyopathy (89). *Fbln5* encodes the protein fibulin-5, a secreted extracellular matrix protein that plays a role in vascular remodeling through promotion of endothelial cell adhesion and upregulation impairs endothelial cell proliferation (90). Others have reported upregulation of *Fbln5* in patients with diabetes, hypertriglyceridemia and a strong family history of CVD (91). In our study, *Fbln5* was upregulated in both diabetes and HF diet exposed offspring hearts. Beyond pathway analyses, these findings identify *Car1, Frzb*, and *Fbln5* as potential candidates in the pathogenesis of neonatal cardiomyopathy and programming of adult CVD in offspring born to diabetic mothers (30).

When comparing HF diet exposed offspring to control samples, Rho family GTPase 1 (*Rnd1*), TNF receptor superfamily member 12A (*Tnfrsf12a*), heparin-binding EGF-like growth factor (*Hbegf* ) and a group of microRNAs, were discretely upregulated in addition to the aforementioned *Fbln5*. MicroRNAs are non-coding RNA, 19-24 nucleotides in length, that are known to regulate almost all biological processes, including glucose and lipid metabolism, apoptosis, signal transduction, and inflammatory responses (92). Specifically, *mir323, mir539, mir377*, and *mir382* all demonstrate significant enrichment in samples taken from the HF diet groups. In the context of cardiac disease, *mir323* has been associated with cardiac exosomal fractions that bind to matrix metalloprotease 9 (MMP9) and downregulate its expression to mitigate MMP9 induced extracellular matrix remodeling (93). In rat models of myocardial infarction, *mir539* expression increases significantly and binds to and inhibits the expression of MEK leading to impaired proliferation and apoptosis of cardiomyocytes (94). *Mir377* also influences myocardial regeneration and angiogenesis by targeting many genes involved in inflammation, oxidative stress, and angiogenesis; converse to *mir539*, it mitigates myocardial injury following ischemia-reperfusion injury by inducing angiogenesis and stem cell proliferation in ischemic myocardium (92, 95). While no cardiac specific associations have been reported for *mir382*, it has been observed to function as a PI3K/Akt pathway inhibitor (96) adding more relevance to network studies.

Other upregulated genes in HF diet exposed offspring hearts included *Fbln5*, as previously noted; *Hbe1, Rnd1, Tnfrsf12a*, and *Hbegf* . *Rnd1* has been identified as a biomechanical stress-sensitive activator of cardiomyocyte growth and hypertrophy in rodent models and overexpression reactivates neonatal rat ventricular myocyte proliferation (97). *Tnfrsf12a* also known as TWEAKR/FN14 is expressed at low basal levels in the heart (98) and sustained expression of the receptor results in adverse cardiac fibrotic remodeling and heart failure as a result of sensitized inflammatory response (99). Indeed, cardiac hypertrophy due to increased fibroblast proliferation downstream of increased TWEAKR activity is supported by JAK2/STAT3 mediated hypertrophy in atrial myocytes (100). *Hbegf* encodes heparin-binding extracellular growth factor protein which increase AKT activity, cardiac fibroblast proliferation and secretion of Type 1 collagen to influence cardiac remodeling, stiffness and contractility (101) which could have an additive effect with overexpressed TWEAKR discussed above.

Significantly downregulated genes in the HF diet group included the general class of regenerating islet-derived protein 3 beta or *Reg3b*, as well as its paralog, *Reg3g*, leucine rich repeat containing 14B (*Lrrc14b*) and DNA damage inducible transcript 4 like (*Ddit4l*). *Reg3b* serves as a cardiomyocyte-derived chemokine for macrophages and is upregulated after myocardial infarction to influence cardiac remodeling (102, 103). *Pik3ip1* is a suppressor of the AKT pathway and was downregulated in maternal high-fat diet exposed offspring compared to controls. Perhaps this, alongside upregulation of *Hbegf* , partially negates the PI3K/AKT suppression associated with diabetes exposure to explain variable phenotypes between the 4 groups and lack of additive effect in the combination exposed group. *Lrrc14b* is normally expressed at low levels in non-germline tissues and has been identified as a paralog of *Lrrc14*, a leucine rich-repeat containing protein that has recently been described as a Toll-like receptor inhibitor (104). Previous work by Pierre et al. indicates that HF diet causes pathological remodeling via Toll-like receptor activation and using TLR4 knockouts, the authors demonstrated that TLR4 deficiency protected against diet-induced ER stress (105). In addition, when considered in the context of cardiomyopathy, ER stress has been associated with cardiometabolic perturbations and cardiomyopathy (106, 107). *Ddit4l* is an autophagy mediator in the heart through mTOR pathways and the balance regulates both physiologic and pathological cardiac hypertrophy (108).

Identification here of the complex metabolic underpinnings of impaired offspring cardiac development subsequent to the combination of maternal diabetes and HF diet is a clear and significant advancement in understanding cardiopathology in the context of DOHaD. Significantly, the findings of the present study establish a precedent to examine longitudinal changes, as “transcriptome snapshots” capture single time points and miss nuances of gene expression dynamics. Network prioritization of FGF and PI3/AKT signaling cascades, as well as their convergence on PGC1α revealed the importance of this cluster in facilitating cardiometabolic derangement (Figure 7). Ultimately, the present findings may also provide a translational advantage by considering maternal hyperlipidemia and the FGF-PI3K/AKT- PGC1α cluster as a targetable hub for prevention and treatment of developmentally programmed heart disease.

## Data Availability Statement

The datasets presented in this study can be found in online repositories. The names of the repository/repositories and accession number(s) can be found below: https://www.ncbi.nlm.nih.gov/geo/, GSE150649.

## Ethics Statement

The animal study was reviewed and approved by Sanford Research Institutional Animal Care and Use Committee.

## Author Contributions

MB and RF are the principal investigators responsible for the work as a whole. MB conceived and funded the study, completed animal work, and RNA extraction. TL did PCR, protein, serum, and tissue analyses. JE isolated NRCM and completed extracellular flux analyses and EL assisted in data interpretation. CP did bioinformatics analysis on transcriptomic data. RF and CP did functional annotation and network analyses. CP drafted the manuscript and figures with creative support from TG. All authors assisted in the project as a whole including manuscript overview.

## Conflict of Interest

The authors declare that the research was conducted in the absence of any commercial or financial relationships that could be construed as a potential conflict of interest.

## Abbreviations

CVD: Cardiovascular disease
GDM: Gestational diabetes mellitus
HF: High fat
ChIP-SEq: Chromatin immunoprecipitation sequencing
GD: Gestational day
P1: Postnatal day one
RIN: RNA integrity number
QC: Quality control
KEGG: Kyoto Encyclopedia of Genes and Genomes
DAVID, Database for Annotation: Visualization and Integrated Discovery
IPA: Ingenuity pathway analysis
cDNA: Complementary DNA
qPCR: Quantitative PCR
OD: Optical density
GST: Glycolytic stress test
NRCM: Neonatal (P1) rat cardiomyocytes
OCR: Oxygen consumption rates
ECAR: Extracellular acidification rates
FCCP: Cyanide-4-(trifluoromethoxy)phenylhydrazone
PPR: Proton production rate
PAS: Periodic acid-Schiff
GEO: Gene expression omnibus
DEG: Differentially expressed gene
IDM: Infants of diabetic mothers.
