# Supplementary material for: Maternal High Fat Diet and Diabetes Disrupts Transcriptomic Pathways That Regulate Cardiac Metabolism and Cell Fate in Newborn Rat Hearts
Source: Front Endocrinol (Lausanne). 2020 Sep 17;11:570846. doi: 10.3389/fendo.2020.570846 (PMC7527411; doi:10.3389/fendo.2020.570846)
Supplement: Supplementary file 1 [file Data_Sheet_1.PDF]

Supplementary Material.

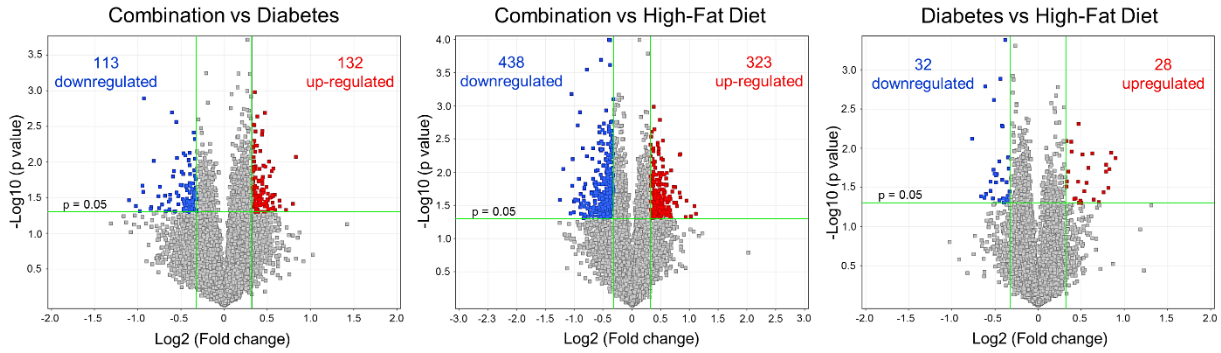

Figure S1. Depicted are the differentially expressed genes (DEG) in the combination exposed group when compared to diabetes (left plot, 113 downregulated and 132 upregulated) and to HF diet groups (middle plot, 438 downregulated and 323 upregulated). Furthermore, when comparing the diabetes exposed group with the HF diet group, 60 DEGs (right plot, 32 downregulated and 28 upregulated) were revealed to be above our set statistical threshold ($p < 0.05$, fold change > 1.25 and < -1.25).

Table S1.
Differentially expressed genes in Combination exposed vs Control group.

	Gene Symbol	Gene Description	Fold Change	Log Fold Change	p Value	Affymetrix ID	Entrez Gene ID
Downregulated	*Mpz*	myelin protein zero	-1.79	-0.84	0.0385	10765508	24564
	RT1-Db2	RT1 class II, locus Db2	-1.62	-0.70	0.0210	10831551	24981
	Drd4	dopamine receptor D4	-1.54	-0.63	0.0127	10712265	25432
	Abca1	ATP binding cassette subfamily A member 1	-1.49	-0.57	0.0372	10876769	313210
	Slco2b1	solute carrier organic anion transporter family, member 2b1	-1.47	-0.56	0.0095	10723902	140860
	Fbxl20	F-box and leucine-rich repeat protein 20	-1.47	-0.55	0.0274	10746854	64039
	Rnf213	ring finger protein 213	-1.45	-0.54	0.0087	10739990	303735
	Slfn5	schlafen family member 5	-1.45	-0.53	0.0225	10736784	303377
	Aplnr	apelin receptor	-1.44	-0.53	0.0430	10837424	83518
	Pde2a	phosphodiesterase 2A	-1.44	-0.52	0.0216	10709200	81743
	Dtx4	deltex E3 ubiquitin ligase 4	-1.42	-0.51	0.0052	10729009	293774
	Vangl1	VANGL planar cell polarity protein 1	-1.42	-0.51	0.0310	10825510	690366
	Smarcd1	SWI/SNF matrix associated, actin dependent regulator of chromatin, subfamily d membe	-1.41	-0.50	0.0442	10899174	363002
	Rgs22	regulator of G-protein signaling 22	-1.40	-0.48	0.0274	10903292	500853
	Kmt5b	lysine methyltransferase 5B, Suv420h1	-1.39	-0.48	0.0445	10712694	361688
	Fgfr2	fibroblast growth factor receptor 2	-1.39	-0.47	0.0089	10726172	25022
	Ntn1	netrin 1	-1.38	-0.47	0.0353	10743774	114523
	Atxn7l1	ataxin 7-like 1	-1.37	-0.45	0.0268	10884189	362726
	Clvs1	clavesin 1	-1.37	-0.45	0.0296	10867587	366311
	Ifi44l	interferon-induced protein 44-like	-1.37	-0.45	0.0354	10834014	310968
	RGD1564899	similar to chromosome 10 open reading frame 71	-1.37	-0.45	0.0241	10790352	498579
	Eya4	EYA transcriptional coactivator and phosphatase 4	-1.36	-0.44	0.0070	10702295	292172
	Lifr	leukemia inhibitory factor receptor alpha	-1.36	-0.44	0.0421	10813445	81680
	Gpr21	G protein-coupled receptor 21	-1.36	-0.44	0.0127	10835940	689917
	Eqtn	equatorin	-1.35	-0.44	0.0186	10878068	500502
	Gabrb2	gamma-aminobutyric acid type A receptor beta 2 subunit	-1.35	-0.43	0.0354	10732824	25451
	Mylk3	myosin light chain kinase 3	-1.35	-0.43	0.0470	10806461	291926
	Atp8a1	ATPase phospholipid transporting 8A1	-1.34	-0.42	0.0288	10772582	289615
	Znf740	zinc finger protein 740	-1.34	-0.42	0.0440	10899487	685834
	Sesn3	sestrin 3	-1.34	-0.42	0.0491	10908037	315427
	Slc44a2	solute carrier family 44 member 2	-1.34	-0.42	0.0357	10908391	363024
	Fam115c	family with sequence similarity 115, member C	-1.33	-0.42	0.0303	10862301	680422
	Cpne5	copine 5	-1.33	-0.41	0.0004	10832034	309650
	Wdr6	WD repeat domain 6	-1.33	-0.41	0.0480	10920313	301007
	Pcdh17	protocadherin 17	-1.32	-0.41	0.0498	10781745	306055
	Rbbp6	RB binding protein 6, ubiquitin ligase	-1.32	-0.40	0.0236	10710674	308968
	Fam227a	family with sequence similarity 227, member A	-1.32	-0.40	0.0252	10897648	300074

Continuation Table S1.

Gene Symbol	Gene Description	Fold Change	Log Fold Change	p Value	Affymetrix ID	Entrez Gene ID
Gatsl2	GATS protein-like 2	-1.32	-0.40	0.0404	10761287	304410
Clec12b	C-type lectin domain family 12, member B	-1.31	-0.39	0.0250	10866005	502900
Fam212b	family with sequence similarity 212, member B	-1.31	-0.39	0.0078	10818155	310764
Nkain2	Na+/K+ transporting ATPase interacting 2	-1.31	-0.39	0.0238	10702330	103690197
Baz2b	bromodomain adjacent to zinc finger domain, 2B	-1.31	-0.39	0.0421	10845470	317627
Adnp	activity-dependent neuroprotector homeobox	-1.31	-0.39	0.0231	10851991	64622
Tnrc6c	trinucleotide repeat containing 6C	-1.31	-0.39	0.0287	10739904	303774
Serping1	serpin family G member 1	-1.31	-0.39	0.0495	10846854	295703
Rc3h1	ring finger and CCCH-type domains 1	-1.30	-0.38	0.0254	10765017	680586
Esr1	estrogen receptor 1	-1.30	-0.38	0.0086	10702695	24890
Polr2a	RNA polymerase II subunit A	-1.30	-0.38	0.0485	10744182	363633
Ep300	E1A binding protein p300	-1.30	-0.37	0.0463	10897919	170915
Magi3	membrane associated guanylate kinase, WW and PDZ domain containing 3	-1.30	-0.37	0.0068	10825609	245903
Lcor	ligand dependent nuclear receptor corepressor	-1.29	-0.37	0.0038	10715344	365462
Marveld2	MARVEL domain containing 2	-1.29	-0.37	0.0170	10820965	365657
Fam171b	family with sequence similarity 171, member B	-1.29	-0.37	0.0103	10837310	499821
Mical3	microtubule associated monooxygenase, calponin and LIM domain containing 3	-1.29	-0.37	0.0493	10865229	362427
Tnxb	tenascin XB	-1.29	-0.37	0.0244	10831384	415089
Nfxl1	nuclear transcription factor, X-box binding-like 1	-1.29	-0.37	0.0141	10772472	289595
Lats1	large tumor suppressor kinase 1	-1.29	-0.37	0.0234	10701709	308265
Plscr4	phospholipid scramblase 4	-1.29	-0.36	0.0241	10912245	300900
Gramd1b	GRAM domain containing 1B	-1.29	-0.36	0.0408	10916432	300644
Rsph4a	radial spoke head 4 homolog A	-1.28	-0.36	0.0425	10830081	309767
N4bp2	NEDD4 binding protein 2	-1.28	-0.36	0.0482	10776778	305342
Notch2	notch 2	-1.28	-0.35	0.0347	10817711	29492
Papln	papilin, proteoglycan-like sulfated glycoprotein	-1.28	-0.35	0.0343	10885823	314297
Mgea5	meningioma expressed antigen 5 (hyaluronidase)	-1.28	-0.35	0.0359	10730452	154968
Mir342	microRNA 342	-1.27	-0.35	0.0168	10886771	100313980
Zbtb34	zinc finger and BTB domain containing 34	-1.27	-0.35	0.0446	10844502	689174
Megf8	multiple EGF-like-domains 8	-1.27	-0.35	0.0325	10705142	114029
Zbtb1	zinc finger and BTB domain containing 1	-1.27	-0.35	0.0138	10885396	314246
Ssh1	slingshot protein phosphatase 1	-1.27	-0.35	0.0460	10759147	304580
Myorg	myogenesis regulating glycosidase, RGD1309821	-1.27	-0.34	0.0223	10876147	57462
Zfp451	zinc finger protein 451	-1.27	-0.34	0.0142	10927233	316312
Myo10	myosin X	-1.27	-0.34	0.0484	10813894	310178
Ino80d	INO80 complex subunit D	-1.27	-0.34	0.0483	10928475	316440
LOC100911949	uncharacterized LOC100911949	-1.27	-0.34	0.0114	10775848	100911949
Mcm9	minichromosome maintenance 9 homologous recombination repair factor	-1.27	-0.34	0.0120	10833361	499437
Ndst2	N-deacetylase and N-sulfotransferase 2	-1.26	-0.34	0.0139	10779054	114002
Slc22a15	solute carrier family 22, member 15	-1.26	-0.34	0.0478	10825495	310732
Prdm10	PR/SET domain 10	-1.26	-0.34	0.0277	10908788	500964
Secisbp2l	SECIS binding protein 2-like	-1.26	-0.34	0.0226	10849436	296115

Continuation Table S1.

	Gene Symbol	Gene Description	Fold Change	Log Fold Change	p Value	Affymetrix ID	Entrez Gene ID
	Itpr3	inositol 1,4,5-trisphosphate receptor, type 3	-1.26	-0.34	0.0339	10828516	25679
	Tnik	TRAF2 and NCK interacting kinase	-1.26	-0.33	0.0089	10814628	294917
	LOC102551046	PHD finger protein 11-like	-1.26	-0.33	0.0066	10784227	102551046
	Rgma	repulsive guidance molecule family member A	-1.26	-0.33	0.0439	10707935	308739
	Safb	scaffold attachment factor B	-1.26	-0.33	0.0266	10931414	64196
	Tet3	tet methylcytosine dioxygenase 3	-1.26	-0.33	0.0302	10863523	680576
	Armcx4	armadillo repeat containing, X-linked 4	-1.26	-0.33	0.0447	10934952	100359678
	Cpeb2	cytoplasmic polyadenylation element binding protein 2	-1.26	-0.33	0.0011	10777290	360949
	Rab11fip2	RAB11 family interacting protein 2	-1.26	-0.33	0.0270	10731140	308003
	Zfp319	zinc finger protein 319	-1.26	-0.33	0.0311	10805903	291849
	Fgf7	fibroblast growth factor 7	-1.25	-0.33	0.0211	10839434	29348
	Ddi1	DNA-damage inducible 1 homolog 1	-1.25	-0.33	0.0018	10914678	367012
	Steap4	STEAP4 metalloreductase	-1.25	-0.33	0.0181	10860597	499991
	Emilin2	elastin microfibril interfacer 2	-1.25	-0.32	0.0119	10930428	316736
	Cyp2d1	cytochrome P450, family 2, subfamily d, polypeptide 1	-1.25	-0.32	0.0069	10905738	266684
Upregulated	*Bdh1*	3-hydroxybutyrate dehydrogenase, type 1	2.29	1.20	0.0227	10754931	117099
	Chrdl1	chordin-like 1	2.11	1.07	0.0405	10932773	363455
	Slc16a3	solute carrier family 16 member 3	1.75	0.81	0.0379	10740209	80878
	Ifit1bl	interferon-induced protein with tetratricopeptide repeats 1B-like	1.70	0.76	0.0463	10729791	294090
	Edn1	endothelin 1	1.68	0.75	0.0261	10797857	24323
	Abhd18	abhydrolase domain containing 18	1.67	0.74	0.0489	10815137	499602
	Gadd45g	growth arrest and DNA-damage-inducible, gamma	1.64	0.71	0.0059	10797527	291005
	Uros	uroporphyrinogen III synthase	1.63	0.71	0.0449	10726346	309070
	Stc1	stanniocalcin 1	1.63	0.70	0.0216	10781273	81801
	Mtfp1	mitochondrial fission process 1	1.61	0.68	0.0455	10778038	289745
	Sctr	secretin receptor	1.60	0.68	0.0448	10931678	81779
	Mcrip2	MAPK regulated co-repressor interacting protein 2	1.60	0.68	0.0381	10741629	685545
	Lsmem2	leucine-rich single-pass membrane protein 2	1.56	0.64	0.0457	10920084	689621
	Tstd3	thiosulfate sulfurtransferase (rhodanese)-like domain containing 3	1.56	0.64	0.0410	10875751	500420
	Ptges3l	prostaglandin E synthase 3 like	1.55	0.63	0.0135	10747550	103693432
	Myl1	myosin, light chain 1	1.55	0.63	0.0116	10928614	56781
	Thumpd3	THUMP domain containing 3	1.54	0.63	0.0334	10857667	500288
	Hapln1	hyaluronan and proteoglycan link protein 1	1.53	0.62	0.0188	10812399	29331
	Blvrb	biliverdin reductase B	1.50	0.58	0.0372	10705358	292737
	Chid1	chitinase domain containing 1	1.49	0.58	0.0452	10726860	293628
	Hikeshi	Hikeshi, heat shock protein nuclear import factor, l7Rn6	1.48	0.57	0.0208	10723601	293103
	Mrpl19	mitochondrial ribosomal protein L19	1.47	0.56	0.0168	10863420	297372
	Hspb3	heat shock protein family B (small) member 3	1.45	0.54	0.0292	10821389	78951
	Tceal7	transcription elongation factor A like 7	1.45	0.53	0.0356	10935041	680319
	Josd2	Josephin domain containing 2	1.44	0.53	0.0327	10706571	292876

Continuation Table S1.

Gene Symbol	Gene Description	Fold Change	Log Fold Change	p Value	Affymetrix ID	Entrez Gene ID
Ap1s2	adaptor-related protein complex 1, sigma 2 subunit	1.44	0.53	0.0358	10937769	302671
Tpmt	thiopurine S-methyltransferase	1.44	0.52	0.0249	10794464	690050
Rtn4ip1	reticulon 4 interacting protein 1	1.42	0.51	0.0334	10830630	309912
RGD1565459	similar to ribosomal protein L10a	1.42	0.51	0.0188	10748496	287782
Tm6sf1	transmembrane 6 superfamily member 1	1.40	0.49	0.0482	10708327	361600
Tnfrsf22	tumor necrosis factor receptor superfamily, member 22	1.40	0.49	0.0377	10727116	686008
Ckb	creatine kinase B	1.40	0.48	0.0045	10892265	24264
RGD1560513	similar to macrophage migration inhibitory factor	1.39	0.48	0.0169	10864439	500271
LOC361016	similar to RIKEN cDNA 4933406L09	1.39	0.48	0.0146	10779436	361016
Bphl	biphenyl hydrolase like	1.39	0.47	0.0383	10798100	361239
Hddc3	HD domain containing 3	1.38	0.47	0.0336	10708230	308758
Zcchc4	zinc finger CCHC-type containing 4	1.38	0.47	0.0099	10777081	360946
Nif3l1	NGG1 interacting factor 3 like 1	1.38	0.46	0.0443	10923567	301431
Atp6v1g2	ATPase H+ transporting V1 subunit G2	1.38	0.46	0.0112	10831152	368044
LOC100361898	uncharacterized LOC100361898	1.37	0.46	0.0249	10714780	100361898
Gstk1	glutathione S-transferase kappa 1	1.37	0.46	0.0358	10855008	297029
Habp2	hyaluronan binding protein 2	1.37	0.45	0.0256	10716210	292126
Chchd4	coiled-coil-helix-coiled-coil-helix domain containing 4	1.37	0.45	0.0250	10864100	312559
Fam96b	family with sequence similarity 96, member B	1.37	0.45	0.0424	10805640	680987
Pde6d	phosphodiesterase 6D	1.37	0.45	0.0425	10929600	363272
Mrps27	mitochondrial ribosomal protein S27	1.36	0.45	0.0270	10812722	361883
Pter	phosphotriesterase related	1.36	0.45	0.0408	10796440	63852
Dus2	dihydrouridine synthase 2	1.36	0.44	0.0482	10807435	291978
Ccdc159	coiled-coil domain containing 159	1.36	0.44	0.0243	10908549	300442
Nppb	natriuretic peptide B	1.35	0.43	0.0388	10873895	25105
LOC685505	similar to coiled-coil-helix-coiled-coil-helix domain containing 4	1.35	0.43	0.0212	10923617	685505
Hccs	holocytochrome c synthase	1.35	0.43	0.0449	10933279	317444
Bckdhb	branched chain keto acid dehydrogenase E1 subunit beta	1.35	0.43	0.0231	10911976	29711
Klhdc1	kelch domain containing 1	1.35	0.43	0.0421	10884934	314190
Creg1	cellular repressor of E1A-stimulated genes 1	1.35	0.43	0.0417	10765335	289185
Cdk1	cyclin-dependent kinase 1	1.35	0.43	0.0392	10829761	54237
Trip10	thyroid hormone receptor interactor 10	1.34	0.42	0.0167	10931182	116717
Fbxo44	F-box protein 44	1.34	0.42	0.0025	10881590	500587
Mrps10	mitochondrial ribosomal protein S10	1.34	0.42	0.0458	10926342	363187
Pgk1	phosphoglycerate kinase 1	1.34	0.42	0.0058	10934610	24644
Dnaja4	DnaJ heat shock protein family (Hsp40) member A4	1.33	0.42	0.0234	10910084	300721
Mmachc	methylmalonic aciduria (cobalamin deficiency) cblC type, with homocystinuria	1.33	0.41	0.0360	10878905	313520
Dguok	deoxyguanosine kinase	1.33	0.41	0.0430	10863542	297389
Bex1	brain expressed, X-linked 1	1.33	0.41	0.0191	10939437	501625
Unc119	unc-119 lipid binding chaperone	1.33	0.41	0.0335	10745074	29402
Trim7	tripartite motif-containing 7	1.33	0.41	0.0301	10733049	303089

Continuation Table S1.

Gene Symbol	Gene Description	Fold Change	Log Fold Change	p Value	Affymetrix ID	Entrez Gene ID
Mpst	mercaptopyruvate sulfurtransferase	1.33	0.41	0.0411	10897446	192172
Cdkn3	cyclin-dependent kinase inhibitor 3	1.32	0.40	0.0402	10779638	289993
Fam162a	family with sequence similarity 162, member A	1.32	0.40	0.0458	10751450	360721
Insig2	insulin induced gene 2	1.32	0.40	0.0294	10767175	288985
Casq1	calsequestrin 1	1.32	0.40	0.0403	10769959	686019
Adpgk	ADP-dependent glucokinase	1.32	0.40	0.0427	10910482	315722
Fam98a	family with sequence similarity 98, member A	1.31	0.39	0.0304	10882896	313873
Kcnmb1	potassium calcium-activated channel subfamily M regulatory beta subunit 1	1.31	0.39	0.0164	10732716	29747
Rexo2	RNA exonuclease 2	1.31	0.39	0.0443	10917095	300689
Actr3b	ARP3 actin related protein 3 homolog B	1.31	0.39	0.0339	10859919	362298
Kng1	kininogen 1	1.31	0.39	0.0478	10751988	24903
Gcdh	glutaryl-CoA dehydrogenase	1.31	0.39	0.0300	10806628	364975
Dhrs11	dehydrogenase/reductase 11	1.31	0.39	0.0368	10745757	360583
Yars	tyrosyl-tRNA synthetase	1.31	0.39	0.0397	10872291	313047
Vamp4	vesicle-associated membrane protein 4	1.31	0.39	0.0365	10765115	364033
Pdxp	pyridoxal phosphatase	1.31	0.39	0.0488	10897524	727679
Nudt5	nudix hydrolase 5	1.31	0.38	0.0490	10799539	361274
Mcat	malonyl-CoA-acyl carrier protein transacylase	1.30	0.38	0.0318	10898160	315173
Actg2	actin, gamma 2, smooth muscle, enteric	1.30	0.38	0.0317	10863549	25365
Mrpl38	mitochondrial ribosomal protein L38	1.30	0.38	0.0453	10749132	303685
Ubl4a	ubiquitin-like 4A	1.30	0.38	0.0460	10936067	293864
Stx8	syntaxin 8	1.30	0.37	0.0361	10734711	59074
Raet1l	retinoic acid early transcript 1L	1.30	0.37	0.0396	10716552	292461
RragB	Ras-related GTP binding B	1.29	0.37	0.0320	10937479	117043
LOC684557	similar to mitochondrial ribosomal protein L40	1.29	0.37	0.0222	10781424	684557
Ndufc1	NADH:ubiquinone oxidoreductase subunit C1	1.29	0.37	0.0117	10823098	689938
Dap3	death associated protein 3	1.29	0.36	0.0205	10824373	295238
Hmbs	hydroxymethylbilane synthase	1.29	0.36	0.0352	10916753	25709
Kdelr3	KDEL endoplasmic reticulum protein retention receptor 3	1.29	0.36	0.0454	10905453	315131
Ostc	oligosaccharyltransferase complex non-catalytic subunit	1.28	0.36	0.0421	10826814	362040
Sirt5	sirtuin 5	1.28	0.36	0.0465	10797827	306840
LOC500028	hypothetical protein LOC500028	1.28	0.36	0.0383	10861026	500028
Odc1	ornithine decarboxylase 1	1.28	0.36	0.0430	10883785	24609
LOC497796	hypothetical protein LOC497796	1.28	0.35	0.0495	10866116	497796
Fam227b	family with sequence similarity 227, member B	1.28	0.35	0.0135	10849472	296118
Dok5	docking protein 5	1.28	0.35	0.0312	10842500	502694
Ldha	lactate dehydrogenase A	1.28	0.35	0.0258	10707137	24533
Tecrl	trans-2,3-enoyl-CoA reductase-like	1.27	0.35	0.0303	10772147	364134
Nudt7	nudix hydrolase 7	1.27	0.35	0.0291	10808159	361413
Nudcd2	NudC domain containing 2	1.27	0.35	0.0344	10732811	287199
Mrpl40	mitochondrial ribosomal protein L40	1.27	0.35	0.0288	10755987	287962

Continuation Table S1.

Gene Symbol	Gene Description	Fold Change	Log Fold Change	p Value	Affymetrix ID	Entrez Gene ID
Tarm1	T cell-interacting, activating receptor on myeloid cells 1	1.27	0.34	0.0038	10703757	499065
Zmat5	zinc finger, matrin type 5	1.27	0.34	0.0373	10773884	501926
Ptx3	pentraxin 3	1.27	0.34	0.0156	10815785	689388
Sike1	suppressor of IKBKE 1	1.27	0.34	0.0378	10817910	362007
Dync2li1	dynein cytoplasmic 2 light intermediate chain 1	1.27	0.34	0.0347	10882627	298767
Cntnap3b	contactin associated protein-like 3B	1.27	0.34	0.0013	10793429	290952
Acy1	aminoacylase 1	1.27	0.34	0.0439	10919897	300981
Apoo	apolipoprotein O	1.26	0.34	0.0199	10934024	363474
L2hgdh	L-2-hydroxyglutarate dehydrogenase	1.26	0.33	0.0351	10890342	314196
Chn3	chimerin 3	1.26	0.33	0.0001	10805591	689153
Dgkg	diacylglycerol kinase, gamma	1.26	0.33	0.0357	10752007	25666
Ufsp2	UFM1-specific peptidase 2	1.26	0.33	0.0490	10791719	361151
Ak2	adenylate kinase 2	1.26	0.33	0.0228	10872260	24184
Spcs3	signal peptidase complex subunit 3	1.26	0.33	0.0351	10788053	680782
Slc15a4	solute carrier family 15 member 4	1.26	0.33	0.0436	10758094	246280
Mpi	mannose phosphate isomerase (mapped)	1.26	0.33	0.0305	10917770	300741
Hectd2	HECT domain E3 ubiquitin protein ligase 2	1.26	0.33	0.0426	10714973	309514
Pebp1	phosphatidylethanolamine binding protein 1	1.26	0.33	0.0473	10758896	29542
Hexb	hexosaminidase subunit beta	1.26	0.33	0.0430	10820693	294673
C6	complement C6	1.25	0.33	0.0481	10813253	24237
Them4	thioesterase superfamily member 4	1.25	0.33	0.0443	10817208	361992
RGD1559534	similar to Alpha enolase (2-phospho-D-glycerate hydro-lyase)	1.25	0.32	0.0143	10742966	287338
LOC654482	hypothetical protein LOC654482	1.25	0.32	0.0201	10914823	654482
RGD1559808	similar to 40S ribosomal protein S26	1.25	0.32	0.0393	10921270	316158
Rexo4	REX4 homolog, 3'-5' exonuclease	1.25	0.32	0.0215	10843909	311826
Myh3	myosin heavy chain 3	1.25	0.32	0.0253	10734493	24583
Tmem242	transmembrane protein 242	1.25	0.32	0.0209	10717855	292228

Table S2.
Differentially expressed genes in Combination exposed vs Diabetes exposed group.

	Gene Symbol	Gene Description	Fold Change	Log Fold Change	p Value	Affymetrix ID	Entrez Gene ID
Downregulated	*Mir32*	microRNA 32	-2.04	-1.03	0.041	10876933	100314013
	Vangl1	VANGL planar cell polarity protein 1	-1.91	-0.93	0.001	10825510	690366
	Rnf213	ring finger protein 213	-1.46	-0.55	0.009	10739990	303735
	Kif26b	kinesin family member 26B	-1.45	-0.53	0.038	10766082	305012
	Bcl2l11	BCL2 like 11	-1.42	-0.51	0.021	10839655	64547
	Pcdh17	protocadherin 17	-1.41	-0.50	0.014	10781745	306055
	Fgfr2	fibroblast growth factor receptor 2	-1.39	-0.48	0.015	10726172	25022
	Dtx4	deltex E3 ubiquitin ligase 4	-1.39	-0.47	0.007	10729009	293774
	Ptpn13	protein tyrosine phosphatase, non-receptor type 13	-1.39	-0.47	0.035	10775429	498331
	Ulk1	unc-51 like autophagy activating kinase 1	-1.38	-0.47	0.038	10759248	360827
	Plin1	perilipin 1	-1.37	-0.46	0.039	10722973	25629
	Coq8a/Adck3	Coenzyme Q8A	-1.37	-0.45	0.034	10770313	56997
	Rgma	repulsive guidance molecule family member A	-1.36	-0.44	0.022	10707935	308739
	Atp1a2	ATPase Na+/K+ transporting subunit alpha 2	-1.36	-0.44	0.022	10769998	24212
	Erbb2	erb-b2 receptor tyrosine kinase 2	-1.36	-0.44	0.021	10737962	24337
	Celsr1	cadherin, EGF LAG seven-pass G-type receptor 1	-1.35	-0.44	0.048	10905988	300128
	Mir29c	microRNA 29c	-1.35	-0.43	0.032	10766882	100314187
	Mical3	microtubule associated monooxygenase, calponin and LIM domain containing 3	-1.35	-0.43	0.035	10865229	362427
	Rbbp6	RB binding protein 6, ubiquitin ligase	-1.34	-0.42	0.047	10710674	308968
	Hoxa2	homeo box A2	-1.34	-0.42	0.039	10862547	103690123
	Ndst2	N-deacetylase and N-sulfotransferase 2	-1.33	-0.42	0.009	10779054	114002
	Gatsl2	GATS protein-like 2	-1.33	-0.41	0.035	10761287	304410
	Fbrsl1	fibrosin-like 1	-1.33	-0.41	0.048	10759358	304572
	Zfyve26	zinc finger FYVE-type containing 26	-1.33	-0.41	0.036	10890809	314265
	Rere	arginine-glutamic acid dipeptide repeats	-1.32	-0.40	0.050	10874166	116665
	Atg13	autophagy related 13	-1.32	-0.40	0.045	10847408	362164
	Slc4a4	solute carrier family 4 member 4	-1.32	-0.40	0.019	10775997	84484
	Tnrc6c	trinucleotide repeat containing 6C	-1.32	-0.40	0.011	10739904	303774
	Thrb	thyroid hormone receptor beta	-1.32	-0.40	0.049	10782454	24831
	Ahnak2	AHNAK nucleoprotein 2	-1.32	-0.40	0.007	10892330	314478
	Cpne5	copine 5	-1.32	-0.40	0.027	10832034	309650
	Dgkz	diacylglycerol kinase zeta	-1.32	-0.40	0.015	10847438	81821
	RGD1309821	similar to KIAA1161 protein	-1.32	-0.40	0.038	10876147	366360
	Srgap1	SLIT-ROBO Rho GTPase activating protein 1	-1.32	-0.40	0.021	10902765	314903
	RGD1564899	similar to chromosome 10 open reading frame 71	-1.31	-0.39	0.035	10790352	498579
	Rc3h1	ring finger and CCCH-type domains 1	-1.31	-0.39	0.035	10765017	680586
	Kdm4a	lysine demethylase 4A	-1.30	-0.38	0.044	10879110	313539
	Znf740	zinc finger protein 740	-1.30	-0.38	0.048	10899487	685834
	Mcm9	minichromosome maintenance 9 homologous recombination repair factor	-1.30	-0.38	0.024	10833361	499437
	Rbm20	RNA binding motif protein 20	-1.30	-0.37	0.049	10716118	309544
	Gpatch8	G patch domain containing 8	-1.30	-0.37	0.048	10747893	685233
	Ep300	E1A binding protein p300	-1.29	-0.37	0.036	10897919	170915
	Mlxip	MLX interacting protein	-1.29	-0.37	0.031	10761897	304479

Continuation Table S2.

	Gene Symbol	Gene Description	Fold Change	Log Fold Change	p Value	Affymetrix ID	Entrez Gene ID
	Tnik	TRAF2 and NCK interacting kinase	-1.29	-0.37	0.034	10814628	294917
	Lcor	ligand dependent nuclear receptor corepressor	-1.29	-0.36	0.020	10715344	365462
	Gpr50	G protein-coupled receptor 50	-1.28	-0.36	0.040	10935795	117097
	Myo10	myosin X	-1.28	-0.35	0.046	10813894	310178
	Tbc1d17	TBC1 domain family, member 17	-1.28	-0.35	0.024	10721486	292886
	Zcchc14	zinc finger CCHC-type containing 14	-1.28	-0.35	0.030	10811501	365018
	Dlg5	discs large MAGUK scaffold protein 5	-1.28	-0.35	0.032	10778920	305645
	Scaf8	SR-related CTD-associated factor 8	-1.28	-0.35	0.042	10702746	245926
	Fam168b	family with sequence similarity 168, member B	-1.27	-0.35	0.031	10927277	690188
	Nkain2	Na+/K+ transporting ATPase interacting 2	-1.27	-0.35	0.013	10702330	103690197
	Ino80d	INO80 complex subunit D	-1.27	-0.35	0.027	10928475	316440
	Trim8	tripartite motif-containing 8	-1.27	-0.34	0.031	10715841	688785
	Gfra1	GDNF family receptor alpha 1	-1.27	-0.34	0.015	10731075	25454
	Ntrk3	neurotrophic receptor tyrosine kinase 3	-1.27	-0.34	0.009	10722864	29613
	Ldoc1	leucine zipper, down-regulated in cancer 1	-1.26	-0.34	0.017	10935662	367956
	Asxl1	additional sex combs like 1, transcriptional regulator	-1.26	-0.33	0.041	10841037	311553
	Cnnm3	cyclin and CBS domain divalent metal cation transport mediator 3	-1.26	-0.33	0.027	10922562	301345
	Tekt1	tektin 1	-1.26	-0.33	0.022	10744700	85270
	LOC100359816	rCG21620-like	-1.26	-0.33	0.017	10761941	100359816
	Plscr4	phospholipid scramblase 4	-1.26	-0.33	0.048	10912245	300900
	Pom121	POM121 transmembrane nucleoporin	-1.25	-0.33	0.039	10757674	113975
	Itga8	integrin subunit alpha 8	-1.25	-0.32	0.050	10799684	364786
	Gramd1b	GRAM domain containing 1B	-1.25	-0.32	0.032	10916432	300644
	Mir20b	microRNA 20b	-1.25	-0.32	0.048	10939781	100314201
Upregulated	*Edn1*	endothelin 1	1.77	0.83	0.008	10797857	24323
	Tstd3	thiosulfate sulfurtransferase (rhodanese)-like domain containing 3	1.56	0.64	0.041	10875751	500420
	Yars2	tyrosyl-tRNA synthetase 2	1.50	0.59	0.046	10752592	287924
	Ptges3l	prostaglandin E synthase 3 like	1.50	0.58	0.028	10747550	103693432
	Nif3l1	NGG1 interacting factor 3 like 1	1.48	0.56	0.043	10923567	301431
	Nmu	neuromedin U	1.47	0.56	0.037	10772231	63887
	Ift43	intraflagellar transport 43	1.47	0.55	0.029	10886097	299209
	Pter	phosphotriesterase related	1.45	0.54	0.031	10796440	63852
	Hspb3	heat shock protein family B (small) member 3	1.44	0.53	0.031	10821389	78951
	Snrpd1	small nuclear ribonucleoprotein D1	1.44	0.52	0.016	10800140	291794
	Slc15a4	solute carrier family 15 member 4	1.43	0.52	0.010	10758094	246280
	Ap1s2	adaptor-related protein complex 1, sigma 2 subunit	1.42	0.51	0.045	10937769	302671
	LOC100361898	coiled-coil-helix-coiled-coil-helix domain containing 4	1.42	0.50	0.027	10714780	100361898
	Gadd45g	growth arrest and DNA-damage-inducible, gamma	1.42	0.50	0.034	10797527	291005
	Sdhaf3	succinate dehydrogenase complex assembly factor 3	1.42	0.50	0.033	10853676	362323
	Mlana	melan-A	1.41	0.50	0.041	10714738	293890
	Hccs	holocytochrome c synthase	1.41	0.49	0.034	10933279	317444
	Vps45	vacuolar protein sorting 45	1.40	0.49	0.037	10825120	64516
	Chchd4	coiled-coil-helix-coiled-coil-helix domain containing 4	1.40	0.49	0.022	10864100	312559

Continuation Table S2.

Gene Symbol	Gene Description	Fold Change	Log Fold Change	p Value	Affymetrix ID	Entrez Gene ID
Klhdc1	kelch domain containing 1	1.39	0.48	0.035	10884934	314190
Creld2	cysteine-rich with EGF-like domains 2	1.38	0.47	0.049	10898456	362978
Tmem160	transmembrane protein 160	1.38	0.47	0.041	10704482	292654
Lyrm1	LYR motif containing 1	1.38	0.47	0.039	10710333	365361
LOC685505	similar to coiled-coil-helix-coiled-coil-helix domain containing 4	1.37	0.46	0.024	10923617	685505
Armc1	armadillo repeat containing 1	1.37	0.46	0.038	10822436	294948
Cyp2e1	cytochrome P450, family 2, subfamily e, polypeptide 1	1.37	0.45	0.026	10712090	25086
Rcan1	regulator of calcineurin 1	1.37	0.45	0.011	10753214	266766
Dnaja4	DnaJ heat shock protein family (Hsp40) member A4	1.36	0.44	0.017	10910084	300721
Hist3h2bb	histone cluster 3, H2bb	1.36	0.44	0.004	10742987	691488
LOC500028	hypothetical protein LOC500028	1.35	0.44	0.024	10861026	500028
Mrpl12	mitochondrial ribosomal protein L12	1.35	0.43	0.049	10740153	303746
Cstf3	cleavage stimulation factor subunit 3	1.35	0.43	0.018	10838197	362178
Yae1d1	Yae1 domain containing 1	1.35	0.43	0.033	10795418	306994
Il33	interleukin 33	1.33	0.42	0.039	10714745	361749
Gli4	GLI family zinc finger 4	1.33	0.41	0.036	10897070	500893
RGD1559808	similar to 40S ribosomal protein S26	1.33	0.41	0.012	10921270	316158
Mrps35	mitochondrial ribosomal protein S35	1.32	0.40	0.033	10859725	297727
RGD1562381	similar to ribosomal protein S17	1.32	0.40	0.018	10890687	314248
Mrpl19	mitochondrial ribosomal protein L19	1.32	0.40	0.049	10863420	297372
Stmn4	stathmin 4	1.31	0.38	0.046	10781197	79423
Nudcd2	NudC domain containing 2	1.30	0.38	0.029	10732811	287199
Rps4y2	ribosomal protein S4, Y-linked 2	1.30	0.38	0.048	10859760	690845
LOC103693118	60S ribosomal protein L37-like	1.29	0.37	0.040	10910722	103693118
Fam98a	family with sequence similarity 98, member A	1.29	0.37	0.042	10882896	313873
Zcchc4	zinc finger CCHC-type containing 4	1.29	0.37	0.037	10777081	360946
Ndufaf4	NADH:ubiquinone oxidoreductase complex assembly factor 4	1.29	0.37	0.044	10867944	362495
LOC684557	similar to mitochondrial ribosomal protein L40	1.29	0.37	0.033	10781424	684557
Htr2a	5-hydroxytryptamine receptor 2A	1.29	0.36	0.043	10781467	29595
Mrpl40	mitochondrial ribosomal protein L40	1.29	0.36	0.030	10755987	287962
Fam96a	family with sequence similarity 96, member A	1.28	0.36	0.038	10911042	300797
LOC688672	similar to eukaryotic translation initiation factor 4A, isoform 1	1.28	0.36	0.026	10884738	688672
Ndufaf1	NADH:ubiquinone oxidoreductase complex assembly factor 1	1.28	0.36	0.045	10848596	296086
Tmem60	transmembrane protein 60	1.28	0.36	0.012	10860269	296761
Ostc	oligosaccharyltransferase complex non-catalytic subunit	1.28	0.36	0.044	10826814	362040
Them4	thioesterase superfamily member 4	1.28	0.36	0.049	10817208	361992
Rasl2-9	RAS-like, family 2, locus 9	1.28	0.35	0.049	10703905	751812
Polr1e	RNA polymerase I subunit E	1.28	0.35	0.049	10868682	313245
Sike1	suppressor of IKBKE 1	1.28	0.35	0.046	10817910	362007
Ly49si2	immunoreceptor Ly49si2	1.28	0.35	0.027	10866134	494207
Atp6v1a	ATPase H+ transporting V1 subunit A	1.28	0.35	0.029	10751161	685232
Lysmd2	LysM domain containing 2	1.28	0.35	0.001	10911707	300839
Klhl6	kelch-like family member 6	1.28	0.35	0.042	10752205	287974
LOC103690975	60S ribosomal protein L30-like	1.28	0.35	0.011	10717798	103690975

Continuation Table S2.

Gene Symbol	Gene Description	Fold Change	Log Fold Change	p Value	Affymetrix ID	Entrez Gene ID
LOC688815	similar to prohibitin	1.27	0.35	0.047	10805784	688815
Cisd2	CDGSH iron sulfur domain 2	1.27	0.35	0.038	10826914	295457
Tarm1	T cell-interacting, activating receptor on myeloid cells 1	1.27	0.35	0.003	10703757	499065
Vmp1	vacuole membrane protein 1	1.27	0.35	0.046	10745933	192129
Vamp4	vesicle-associated membrane protein 4	1.27	0.35	0.044	10765115	364033
LOC103692338	putative thymosin beta-4-like protein 6	1.27	0.35	0.005	10876291	103692338
RGD1306063	similar to HT021	1.27	0.34	0.049	10782599	289928
Ak2	adenylate kinase 2	1.27	0.34	0.024	10872260	24184
Gm8444	Uncharacterized protein; Predicted gene 8444	1.26	0.34	0.006	10932893	---
Spcs3	signal peptidase complex subunit 3	1.26	0.33	0.034	10788053	680782
Tmem242	transmembrane protein 242	1.26	0.33	0.018	10717855	292228
Tmed5	transmembrane p24 trafficking protein 5	1.26	0.33	0.047	10771004	289883
Gdf15	growth differentiation factor 15	1.26	0.33	0.019	10787517	29455
Nudt7	nudix hydrolase 7	1.26	0.33	0.034	10808159	361413
Ufsp2	UFM1-specific peptidase 2	1.26	0.33	0.046	10791719	361151
Apoo	apolipoprotein O	1.26	0.33	0.017	10934024	363474
LOC691325	similar to oocyte specific homeobox 3	1.25	0.33	0.006	10703873	691325
Mrpl47	mitochondrial ribosomal protein L47	1.25	0.33	0.032	10822726	294963
Tma7	translation machinery associated 7 homolog	1.25	0.33	0.029	10920371	679747
LOC100909892	ubiquitin-conjugating enzyme E2 N-like	1.25	0.32	0.016	10720520	100909892
Timm21	translocase of inner mitochondrial membrane 21	1.25	0.32	0.045	10805571	307210
LOC501599	similar to galectin 8	1.25	0.32	0.022	10938981	501599

Table S3.
Differentially expressed genes in Combination exposed vs High-Fat diet exposed group.

	Gene Symbol	Gene Description	Fold Change	Log Fold Change	p Value	Affymetrix ID	Entrez Gene ID
Downregulated	*Mir23b*	microRNA 23b	-1.98	-0.99	0.026	10796989	100314002
	Vangl1	VANGL planar cell polarity protein 1	-1.93	-0.95	0.002	10825510	690366
	LOC688452	hypothetical protein LOC688452	-1.86	-0.89	0.021	10703432	688452
	Mir539	microRNA 539	-1.81	-0.86	0.004	10887086	100314167
	Mir380	microRNA 380	-1.79	-0.84	0.027	10887054	100314173
	Mir382	microRNA 382	-1.73	-0.79	0.029	10887090	100314085
	RGD1566401	similar to GTL2, imprinted maternally expressed untranslated	-1.73	-0.79	0.000	10886816	500717
	Mir493	microRNA 493	-1.71	-0.78	0.035	10886826	100314081
	Ttn	titin	-1.66	-0.73	0.040	10846599	84015
	Abca1	ATP binding cassette subfamily A member 1	-1.63	-0.71	0.021	10876769	313210
	Tnrc6b	trinucleotide repeat containing 6B	-1.61	-0.69	0.036	10897852	192178
	Leng8	leukocyte receptor cluster member 8	-1.60	-0.68	0.004	10719006	361506
	Nuak1	NUAK family kinase 1	-1.56	-0.64	0.016	10894552	299694
	Fras1	Fraser extracellular matrix complex subunit 1	-1.56	-0.64	0.029	10775647	289486
	LOC100361083	hypothetical LOC100361083	-1.54	-0.63	0.016	10879660	100361083
	Chd7	chromodomain helicase DNA binding protein 7	-1.54	-0.63	0.009	10867539	312974
	Mir186	microRNA 186	-1.54	-0.62	0.022	10819903	100314043
	Kif1b	kinesin family member 1B	-1.52	-0.60	0.048	10881669	117548
	Stard9	StAR-related lipid transfer domain containing 9	-1.52	-0.60	0.035	10839060	691920
	Crispld2	cysteine-rich secretory protein LCCL domain containing 2	-1.51	-0.59	0.022	10808377	171547
	Mirlet7e	microRNA let-7e	-1.50	-0.58	0.046	10703428	100313991
	Mir154	microRNA 154	-1.50	-0.58	0.034	10887098	100314039
	Mir350	microRNA 350	-1.49	-0.58	0.004	10770195	100313985
	Kif26b	kinesin family member 26B	-1.49	-0.58	0.019	10766082	305012
	Hmbox1	homeobox containing 1	-1.49	-0.57	0.015	10784523	305968
	Per2	period circadian clock 2	-1.49	-0.57	0.007	10929842	63840
	Fgfr2	fibroblast growth factor receptor 2	-1.48	-0.56	0.002	10726172	25022
	Adprhl1	ADP-ribosylhydrolase like 1	-1.48	-0.56	0.002	10789502	290880
	Mir103a2	MicroRNA 103a-2	-1.47	-0.56	0.041	10840061	406896
	Ppp1r9a	protein phosphatase 1, regulatory subunit 9A	-1.47	-0.55	0.030	10853614	84685
	Lcat	lecithin cholesterol acyltransferase	-1.46	-0.54	0.005	10810736	24530
	LOC102555131	afadin-like	-1.45	-0.53	0.021	10718134	102555131
	Notch2	notch 2	-1.45	-0.53	0.005	10817711	29492
	Dact1	dishevelled-binding antagonist of beta-catenin 1	-1.45	-0.53	0.047	10885110	500666
	Sacs	sacsin molecular chaperone	-1.44	-0.53	0.003	10780908	305940
	Armcx4	armadillo repeat containing, X-linked 4	-1.44	-0.53	0.022	10934952	100359678
	LOC103693763	dachshund homolog 1-like	-1.44	-0.53	0.030	10785545	103693763
	Tnks	tankyrase	-1.44	-0.52	0.042	10788538	290794
	Zfp867	zinc finger protein 867	-1.43	-0.52	0.012	10743144	100125361
	Capn6	calpain 6	-1.43	-0.52	0.025	10932759	83685
	Mical3	microtubule associated monooxygenase, calponin and LIM domain containing 3	-1.43	-0.52	0.015	10865229	362427
	Fat4	FAT atypical cadherin 4	-1.43	-0.52	0.027	10815057	310341
	Srgap3	SLIT-ROBO Rho GTPase activating protein 3	-1.43	-0.52	0.033	10864590	500287
	Dock9	dedicator of cytokinesis 9	-1.43	-0.51	0.028	10785964	259237
	Cpne5	copine 5	-1.43	-0.51	0.002	10832034	309650
	Rnf213	ring finger protein 213	-1.42	-0.51	0.033	10739990	303735

Continuation Table S3.

Gene Symbol	Gene Description	Fold Change	Log Fold Change	p Value	Affymetrix ID	Entrez Gene ID
Fry	FRY microtubule binding protein	-1.42	-0.51	0.028	10759852	304244
Plxnb1	plexin B1	-1.42	-0.51	0.024	10913501	316009
Sdk1	sidekick cell adhesion molecule 1	-1.41	-0.50	0.008	10760557	304297
Chd2	chromodomain helicase DNA binding protein 2	-1.41	-0.50	0.030	10722770	308738
Syne2	spectrin repeat containing nuclear envelope protein 2	-1.41	-0.50	0.023	10885365	366669
Rfx5	regulatory factor X5	-1.41	-0.50	0.044	10817297	310659
Mgea5	meningioma expressed antigen 5 (hyaluronidase)	-1.41	-0.50	0.008	10730452	154968
Chd3	chromodomain helicase DNA binding protein 3	-1.41	-0.50	0.034	10743914	303241
Fus	fused in sarcoma RNA binding protein	-1.41	-0.50	0.015	10711251	317385
Kmt2d	lysine methyltransferase 2D	-1.41	-0.49	0.043	10906977	100362634
Smarcd1	SWI/SNF matrix associated, actin dependent regulator of chromatin, subfamily d member1	-1.41	-0.49	0.046	10899174	363002
LOC100363361	zinc finger protein 687-like	-1.40	-0.49	0.035	10824904	100363361
Adamts6	ADAM metallopeptidase with thrombospondin type 1 motif, 6	-1.40	-0.49	0.039	10812834	361886
Baz2b	bromodomain adjacent to zinc finger domain, 2B	-1.40	-0.49	0.013	10845470	317627
Apc	APC, WNT signaling pathway regulator	-1.40	-0.49	0.024	10800844	24205
Spen	spen family transcriptional repressor	-1.40	-0.49	0.002	10881109	690911
Creb5	cAMP responsive element binding protein 5	-1.40	-0.49	0.020	10855601	500131
Cacna1c	calcium voltage-gated channel subunit alpha1 C	-1.40	-0.48	0.046	10865016	24239
Pkd1	polycystic kidney disease 1	-1.40	-0.48	0.026	10732113	24650
Rbm33	RNA binding motif protein 33	-1.39	-0.48	0.012	10859868	362297
Tnik	TRAF2 and NCK interacting kinase	-1.39	-0.48	0.003	10814628	294917
Antxr1	anthrax toxin receptor 1	-1.39	-0.48	0.028	10863777	362393
Jmjd1c	jumonji domain containing 1C	-1.39	-0.47	0.037	10832829	171120
LOC102552104	MLV-related proviral Env polyprotein-like	-1.39	-0.47	0.035	10724341	102552104
Tanc2	tetratricopeptide repeat, ankyrin repeat and coiled-coil containing 2	-1.39	-0.47	0.035	10739003	303599
Chst15	carbohydrate sulfotransferase 15	-1.38	-0.47	0.039	10726269	286974
Tnrc18	trinucleotide repeat containing 18	-1.38	-0.47	0.029	10756815	304302
Celsr1	cadherin, EGF LAG seven-pass G-type receptor 1	-1.38	-0.47	0.032	10905988	300128
LOC103690166	zinc finger protein OZF-like	-1.38	-0.46	0.035	10915740	103690166
Plekhn1	pleckstrin homology domain containing N1	-1.38	-0.46	0.023	10882319	298694
Pgbd1	piggyBac transposable element derived 1	-1.38	-0.46	0.025	10795327	103694097
Arid2	AT-rich interaction domain 2	-1.38	-0.46	0.033	10898911	366980
Tnrc6c	trinucleotide repeat containing 6C	-1.38	-0.46	0.006	10739904	303774
Dsg2	desmoglein 2	-1.37	-0.46	0.048	10800410	307562
Atp8a1	ATPase phospholipid transporting 8A1	-1.37	-0.46	0.022	10772582	289615
Ifit1bl	interferon-induced protein with tetratricopeptide repeats 1B-like	-1.37	-0.46	0.038	10729793	294090
Mast4	microtubule associated serine/threonine kinase family member 4	-1.37	-0.46	0.044	10821072	100912235
Ctnnd1	catenin delta 1	-1.37	-0.46	0.033	10846794	311163
Fam212b	family with sequence similarity 212, member B	-1.37	-0.45	0.008	10818155	310764
Chd6	chromodomain helicase DNA binding protein 6	-1.37	-0.45	0.038	10851407	311607
LOC102548231	zinc finger protein 850-like	-1.37	-0.45	0.001	10706242	102548231
Mir341	microRNA 341	-1.37	-0.45	0.046	10886842	100313979
Kank1	KN motif and ankyrin repeat domains 1	-1.37	-0.45	0.005	10714505	309429
Lrrc29	leucine rich repeat containing 29	-1.36	-0.45	0.017	10810585	502201
Dgkh	diacylglycerol kinase, eta	-1.36	-0.45	0.015	10785405	361076
Tnrc6a	trinucleotide repeat containing 6a	-1.36	-0.45	0.035	10710695	308971
Pdzrn3	PDZ domain containing RING finger 3	-1.36	-0.44	0.007	10864459	312607

Continuation Table S3.

Gene Symbol	Gene Description	Fold Change	Log Fold Change	p Value	Affymetrix ID	Entrez Gene ID
Akap13	A-kinase anchoring protein 13	-1.36	-0.44	0.044	10707961	293024
Rere	arginine-glutamic acid dipeptide repeats	-1.36	-0.44	0.035	10874166	116665
Lats1	large tumor suppressor kinase 1	-1.36	-0.44	0.010	10701709	308265
RGD1562037	similar to OTTHUMP00000046255	-1.36	-0.44	0.018	10798828	498764
Ubr4	ubiquitin protein ligase E3 component n-recognin 4	-1.36	-0.44	0.037	10873419	313658
Zfp26	zinc finger protein 26	-1.36	-0.44	0.023	10915316	367033
Bcl6b	B-cell CLL/lymphoma 6B	-1.36	-0.44	0.009	10744376	360551
Sfswap	splicing factor SWAP homolog	-1.35	-0.44	0.015	10761411	304431
Slfn5	schlafen family member 5	-1.35	-0.44	0.019	10736784	303377
Alpk3	alpha-kinase 3	-1.35	-0.43	0.037	10708247	365298
Rbbp6	RB binding protein 6, ubiquitin ligase	-1.35	-0.43	0.008	10710674	308968
Tgm1	transglutaminase 1	-1.35	-0.43	0.045	10783880	60335
Ptprs	protein tyrosine phosphatase, receptor type, S	-1.35	-0.43	0.047	10930942	25529
Safb2	scaffold attachment factor B2	-1.35	-0.43	0.010	10930918	301126
Pnn	pinin, desmosome associated protein	-1.35	-0.43	0.018	10884740	368070
Asap1	ArfGAP with SH3 domain, ankyrin repeat and PH domain 1	-1.35	-0.43	0.030	10904031	314961
Stk38l	serine/threonine kinase 38 like	-1.35	-0.43	0.021	10859660	691337
Obscn	obscurin, cytoskeletal calmodulin and titin-interacting RhoGEF	-1.35	-0.43	0.042	10743013	338458
Szt2	seizure threshold 2 homolog (mouse)	-1.34	-0.43	0.029	10879223	362573
Hcfc1	host cell factor C1	-1.34	-0.42	0.041	10940271	363519
Usp49	ubiquitin specific peptidase 49	-1.34	-0.42	0.011	10926317	316211
Pnisr	PNN interacting serine and arginine rich protein	-1.34	-0.42	0.034	10867882	297942
LOC102554570	uncharacterized LOC102554570	-1.34	-0.42	0.013	10857403	102554570
Wdfy3	WD repeat and FYVE domain containing 3	-1.34	-0.42	0.034	10771287	305164
LOC100362344	mKIAA1238 protein-like	-1.34	-0.42	0.006	10867020	100362344
Pcdhga6	protocadherin gamma subfamily A, 6	-1.34	-0.42	0.041	10801254	102552796
Scaf8	SR-related CTD-associated factor 8	-1.34	-0.42	0.011	10702746	245926
Mir501	microRNA 501	-1.34	-0.42	0.012	10932578	100314166
Mapkbp1	mitogen activated protein kinase binding protein 1	-1.34	-0.42	0.021	10838927	362197
Lcor	ligand dependent nuclear receptor corepressor	-1.34	-0.42	0.004	10715344	365462
Myo9b	myosin IXb	-1.34	-0.42	0.039	10787212	25486
Cry2	cryptochrome circadian clock 2	-1.34	-0.42	0.047	10847525	170917
Olr803	olfactory receptor 803	-1.34	-0.42	0.013	10862285	405143
LOC691387	similar to HBxAg transactivated protein 2	-1.34	-0.42	0.034	10869659	691387
Unc13c	unc-13 homolog C	-1.33	-0.42	0.007	10918674	286931
Rc3h1	ring finger and CCCH-type domains 1	-1.33	-0.42	0.010	10765017	680586
Rev3l	REV3 like, DNA directed polymerase zeta catalytic subunit	-1.33	-0.42	0.047	10830408	309812
Ka11	type I keratin KA11	-1.33	-0.41	0.020	10747288	450226
Arhgap26	Rho GTPase activating protein 26	-1.33	-0.41	0.027	10801308	307459
Pgghg/Athl1	Protein-Glucosylgalactosylhydroxylysine Glucosidase	-1.33	-0.41	0.044	10712157	80162
Peak1	pseudopodium-enriched atypical kinase 1	-1.33	-0.41	0.033	10917707	315686
Ddx42	DEAD-box helicase 42	-1.33	-0.41	0.012	10739117	303607
Pcdh18	protocadherin 18	-1.33	-0.41	0.007	10823052	295027
Phldb2	pleckstrin homology-like domain, family B, member 2	-1.33	-0.41	0.047	10751018	685611
Mir199a2	microRNA 199a-2	-1.33	-0.41	0.046	10765105	100314245
Kdm4a	lysine demethylase 4A	-1.33	-0.41	0.045	10879110	313539
Rsph4a	radial spoke head 4 homolog A	-1.33	-0.41	0.002	10830081	309767

Continuation Table S3.

Gene Symbol	Gene Description	Fold Change	Log Fold Change	p Value	Affymetrix ID	Entrez Gene ID
Rrh	retinal pigment epithelium derived rhodopsin homolog	-1.33	-0.41	0.002	10826764	310869
Ssh1	slingshot protein phosphatase 1	-1.33	-0.41	0.023	10759147	304580
Rock2	Rho-associated coiled-coil containing protein kinase 2	-1.33	-0.41	0.011	10883726	25537
Dopey2	dopey family member 2	-1.32	-0.40	0.018	10750333	304077
Gpatch8	G patch domain containing 8	-1.32	-0.40	0.049	10747893	685233
Eml5	echinoderm microtubule associated protein like 5	-1.32	-0.40	0.002	10891548	444982
Etnk1	ethanolamine kinase 1	-1.32	-0.40	0.000	10859590	312828
Plekha5	pleckstrin homology domain containing A5	-1.32	-0.40	0.046	10859438	246237
Tacc1	transforming, acidic coiled-coil containing protein 1	-1.32	-0.40	0.009	10788864	306562
Wnk1	WNK lysine deficient protein kinase 1	-1.32	-0.40	0.038	10865163	116477
Rab11fip2	RAB11 family interacting protein 2	-1.32	-0.40	0.013	10731140	308003
Snrnp70	small nuclear ribonucleoprotein U1 subunit 70	-1.32	-0.40	0.030	10721802	361574
Brwd1	bromodomain and WD repeat domain containing 1	-1.32	-0.40	0.014	10753300	304061
Scd2	stearoyl-Coenzyme A desaturase 2	-1.32	-0.40	0.019	10715546	83792
Marveld2	MARVEL domain containing 2	-1.32	-0.40	0.013	10820965	365657
Cwc22	CWC22 spliceosome associated protein homolog	-1.32	-0.40	0.029	10846661	362153
Adgrl1	adhesion G protein-coupled receptor L1	-1.32	-0.40	0.040	10810236	65096
Rimklb	ribosomal modification protein rimK-like family member B	-1.32	-0.40	0.014	10865321	362428
Vash2	vasohibin 2	-1.32	-0.40	0.033	10770702	498309
Ptpn13	protein tyrosine phosphatase, non-receptor type 13	-1.31	-0.39	0.049	10775429	498331
Fam171b	family with sequence similarity 171, member B	-1.31	-0.39	0.031	10837310	499821
Btn2a2	butyrophilin, subfamily 2, member A2	-1.31	-0.39	0.020	10795233	306957
Pitpnm2	phosphatidylinositol transfer protein, membrane-associated 2	-1.31	-0.39	0.042	10758302	304474
Kmt2e	lysine methyltransferase 2E	-1.31	-0.39	0.028	10860152	311968
Rpgr	retinitis pigmentosa GTPase regulator	-1.31	-0.39	0.018	10932343	367733
Ahnak	AHNAK nucleoprotein	-1.31	-0.39	0.018	10713796	191572
LOC680039	hypothetical protein LOC680039	-1.31	-0.39	0.013	10773333	680039
Nnat	neuronatin	-1.31	-0.39	0.024	10841637	94270
Mib1	mindbomb E3 ubiquitin protein ligase 1	-1.31	-0.39	0.040	10800142	307594
Mir342	microRNA 342	-1.31	-0.39	0.029	10886771	100313980
Piezo1	piezo-type mechanosensitive ion channel component 1	-1.31	-0.39	0.039	10811596	361430
Dgkz	diacylglycerol kinase zeta	-1.31	-0.39	0.030	10847438	81821
N4bp2	NEDD4 binding protein 2	-1.31	-0.38	0.038	10776778	305342
Tenm4	teneurin transmembrane protein 4	-1.31	-0.38	0.021	10708695	308831
Phactr2	phosphatase and actin regulator 2	-1.31	-0.38	0.049	10716896	308291
Cpeb4	cytoplasmic polyadenylation element binding protein 4	-1.30	-0.38	0.022	10741814	303010
Gan	gigaxonin	-1.30	-0.38	0.000	10808235	307893
Il1rl2	interleukin 1 receptor-like 2	-1.30	-0.38	0.009	10922845	171106
Map4k4	mitogen-activated protein kinase kinase kinase kinase 4	-1.30	-0.38	0.045	10922783	301363
Sema6a	semaphorin 6A	-1.30	-0.38	0.049	10804415	361324
Trio	trio Rho guanine nucleotide exchange factor	-1.30	-0.38	0.036	10822113	310192
LOC102546902	protein sidekick-1-like	-1.30	-0.38	0.000	10760559	102546902
Eya4	EYA transcriptional coactivator and phosphatase 4	-1.30	-0.38	0.005	10702295	292172
Hnrnpd	heterogeneous nuclear ribonucleoprotein D	-1.30	-0.38	0.017	10771475	79256
Mcm9	minichromosome maintenance 9 homologous recombination repair factor	-1.30	-0.38	0.006	10833361	499437
Rps6ka5	ribosomal protein S6 kinase A5	-1.30	-0.38	0.046	10891659	314384
Phex	phosphate regulating endopeptidase homolog, X-linked	-1.30	-0.38	0.023	10933664	25512

Continuation Table S3.

Gene Symbol	Gene Description	Fold Change	Log Fold Change	p Value	Affymetrix ID	Entrez Gene ID
Piezo2	piezo-type mechanosensitive ion channel component 2	-1.30	-0.38	0.032	10804843	307380
Dicer1	dicer 1 ribonuclease III	-1.30	-0.38	0.042	10892007	299284
Ep300	E1A binding protein p300	-1.30	-0.38	0.026	10897919	170915
Adamts10	ADAM metallopeptidase with thrombospondin type 1 motif, 10	-1.30	-0.38	0.020	10894306	314655
Prkce	protein kinase C, epsilon	-1.30	-0.38	0.033	10882709	29340
Kat6a	lysine acetyltransferase 6A	-1.30	-0.38	0.044	10792402	306571
Bach2	BTB domain and CNC homolog 2	-1.30	-0.38	0.048	10868000	313125
Itpr3	inositol 1,4,5-trisphosphate receptor, type 3	-1.30	-0.38	0.010	10828516	25679
Kcna5	potassium voltage-gated channel subfamily A member 5	-1.30	-0.38	0.010	10865730	25470
LOC685590	similar to Phosphatidylinositol-4,5-bisphosphate 3-kinase catalytic subunit alpha isoform	-1.30	-0.38	0.020	10937594	685590
Cdk12	cyclin-dependent kinase 12	-1.30	-0.37	0.005	10737927	192350
Zbtb34	zinc finger and BTB domain containing 34	-1.30	-0.37	0.033	10844502	689174
Arhgap42	Rho GTPase activating protein 42	-1.30	-0.37	0.018	10914877	500943
Baz2a	bromodomain adjacent to zinc finger domain, 2A	-1.29	-0.37	0.045	10892980	304601
Pik3ca	phosphatidylinositol-4,5-bisphosphate 3-kinase, catalytic subunit alpha	-1.29	-0.37	0.025	10814726	170911
Kcnn2	potassium calcium-activated channel subfamily N member 2	-1.29	-0.37	0.036	10801520	54262
LOC100911949	uncharacterized LOC100911949	-1.29	-0.37	0.003	10775848	100911949
Tie1	tyrosine kinase with immunoglobulin-like and EGF-like domains 1	-1.29	-0.37	0.015	10879278	89806
LOC103695059	zinc finger protein 2-like	-1.29	-0.37	0.002	10726652	103695059
Safb	scaffold attachment factor B	-1.29	-0.37	0.020	10931414	64196
Zfp612	zinc finger protein 612	-1.29	-0.37	0.004	10807828	307839
Rasa2	RAS p21 protein activator 2	-1.29	-0.37	0.025	10919389	25597
Cdk14	cyclin-dependent kinase 14	-1.29	-0.37	0.033	10853453	362316
Ash1l	ASH1 like histone lysine methyltransferase	-1.29	-0.37	0.046	10816649	310638
Zfp791	zinc finger protein 791	-1.29	-0.37	0.039	10806541	100363196
Hnrnpdl	heterogeneous nuclear ribonucleoprotein D-like	-1.29	-0.37	0.027	10771465	305178
Peg3	paternally expressed 3	-1.29	-0.36	0.042	10703838	103691034
Zfp704	zinc finger protein 704	-1.29	-0.36	0.028	10814310	310233
Nrip1	nuclear receptor interacting protein 1	-1.29	-0.36	0.003	10752754	304157
Syngap1	synaptic Ras GTPase activating protein 1	-1.29	-0.36	0.048	10828477	192117
Pom121	POM121 transmembrane nucleoporin	-1.29	-0.36	0.019	10757674	113975
Arid1b	AT-rich interaction domain 1B	-1.29	-0.36	0.042	10702794	282546
RGD1562080	similar to Hypothetical protein CBG10141	-1.29	-0.36	0.016	10803440	498827
Tjp1	tight junction protein 1	-1.29	-0.36	0.043	10722589	292994
Ino80d	INO80 complex subunit D	-1.29	-0.36	0.020	10928475	316440
Kmt2c	lysine methyltransferase 2C	-1.29	-0.36	0.049	10852742	502710
Aff4	AF4/FMR2 family, member 4	-1.29	-0.36	0.007	10733484	303132
Ndst2	N-deacetylase and N-sulfotransferase 2	-1.28	-0.36	0.037	10779054	114002
Ank2	ankyrin 2	-1.28	-0.36	0.043	10826616	362036
Greb1l	growth regulation by estrogen in breast cancer 1 like	-1.28	-0.36	0.037	10800126	498819
Phf12	PHD finger protein 12	-1.28	-0.36	0.019	10736143	303274
Tet3	tet methylcytosine dioxygenase 3	-1.28	-0.36	0.019	10863523	680576
Pcdh9	protocadherin 9	-1.28	-0.36	0.019	10785523	306091
Rasal2	RAS protein activator like 2	-1.28	-0.36	0.028	10769047	304893
Usp42	ubiquitin specific peptidase 42	-1.28	-0.36	0.016	10756704	288482
Arap3	ArfGAP with RhoGAP domain, ankyrin repeat and PH domain 3	-1.28	-0.36	0.015	10804086	361314
Ankrd52	ankyrin repeat domain 52	-1.28	-0.35	0.005	10893168	362811

Continuation Table S3.

Gene Symbol	Gene Description	Fold Change	Log Fold Change	p Value	Affymetrix ID	Entrez Gene ID
Shank3	SH3 and multiple ankyrin repeat domains 3	-1.28	-0.35	0.035	10898622	59312
Srgap1	SLIT-ROBO Rho GTPase activating protein 1	-1.28	-0.35	0.002	10902765	314903
Plekhg1	pleckstrin homology and RhoGEF domain containing G1	-1.28	-0.35	0.010	10702611	679812
Flna	filamin A	-1.28	-0.35	0.033	10936086	293860
Eqtn	equatorin	-1.28	-0.35	0.025	10878068	500502
Plekhm3	pleckstrin homology domain containing M3	-1.28	-0.35	0.004	10928535	316455
Usf3	upstream transcription factor family member 3	-1.28	-0.35	0.026	10754077	303946
Sned1	sushi, nidogen and EGF-like domains 1	-1.28	-0.35	0.013	10925526	316638
Klhl17	kelch-like family member 17	-1.28	-0.35	0.041	10882340	246757
Smad5	SMAD family member 5	-1.28	-0.35	0.002	10797127	59328
Nfrkb	nuclear factor related to kappa B binding protein	-1.28	-0.35	0.019	10908833	315523
Magi1	membrane associated guanylate kinase, WW and PDZ domain containing 1	-1.28	-0.35	0.020	10864243	500261
Taok2	TAO kinase 2	-1.28	-0.35	0.027	10725854	64666
Ssh2	slingshot protein phosphatase 2	-1.28	-0.35	0.039	10745424	303342
Sash1	SAM and SH3 domain containing 1	-1.27	-0.35	0.030	10716679	365037
Zfp646	zinc finger protein 646	-1.27	-0.35	0.027	10711231	309003
Etnk2	ethanolamine kinase 2	-1.27	-0.35	0.017	10764034	360843
Nfxl1	nuclear transcription factor, X-box binding-like 1	-1.27	-0.35	0.004	10772472	289595
Stxbp5	syntaxin binding protein 5	-1.27	-0.35	0.048	10716712	81022
Zbtb39	zinc finger and BTB domain containing 39	-1.27	-0.35	0.003	10895988	299510
Gba2	glucosidase beta 2	-1.27	-0.35	0.038	10876396	298399
Klf12	Kruppel-like factor 12	-1.27	-0.35	0.046	10785590	306110
RT1-Db2	RT1 class II, locus Db2	-1.27	-0.35	0.003	10831551	24981
Slc7a1	solute carrier family 7 member 1	-1.27	-0.35	0.029	10756393	25648
Tgfbr1	transforming growth factor, beta receptor 1	-1.27	-0.35	0.006	10868923	29591
Iws1	IWS1, SUPT6H interacting protein	-1.27	-0.35	0.009	10800709	291705
Myef2	myelin expression factor 2	-1.27	-0.35	0.039	10849307	679712
Trafd1	TRAF type zinc finger domain containing 1	-1.27	-0.34	0.036	10758711	114635
Sorbs1	sorbin and SH3 domain containing 1	-1.27	-0.34	0.024	10730065	686098
Mlxip	MLX interacting protein	-1.27	-0.34	0.025	10761897	304479
Asxl3	additional sex combs like 3, transcriptional regulator	-1.27	-0.34	0.046	10800456	307555
Tomm5	translocase of outer mitochondrial membrane 5	-1.27	-0.34	0.024	10876507	680080
Tnxb	tenascin XB	-1.27	-0.34	0.037	10831384	415089
Ubr5	ubiquitin protein ligase E3 component n-recognin 5	-1.27	-0.34	0.033	10903400	117060
Dlg5	discs large MAGUK scaffold protein 5	-1.27	-0.34	0.035	10778920	305645
Dennd1a	DENN domain containing 1A	-1.27	-0.34	0.044	10844827	311913
Btbd7	BTB domain containing 7	-1.27	-0.34	0.047	10891867	362772
Hils1	histone linker H1 domain, spermatid-specific 1	-1.27	-0.34	0.023	10737585	690026
Megf8	multiple EGF-like-domains 8	-1.27	-0.34	0.033	10705142	114029
Ahnak2	AHNAK nucleoprotein 2	-1.26	-0.34	0.016	10892330	314478
Wdr90	WD repeat domain 90	-1.26	-0.34	0.018	10741608	102555298
Plag1	PLAG1 zinc finger	-1.26	-0.34	0.038	10875282	297804
Dnm3	dynamin 3	-1.26	-0.34	0.035	10769271	171574
Asxl1	additional sex combs like 1, transcriptional regulator	-1.26	-0.34	0.004	10841037	311553
Zswim8	zinc finger, SWIM-type containing 8	-1.26	-0.34	0.010	10782284	361004
Mtfr2	mitochondrial fission regulator 2	-1.26	-0.34	0.023	10701990	100911069
Emilin2	elastin microfibril interfacer 2	-1.26	-0.34	0.015	10930428	316736

Continuation Table S3.

	Gene Symbol	Gene Description	Fold Change	Log Fold Change	p Value	Affymetrix ID	Entrez Gene ID
	Myo19	myosin XIX	-1.26	-0.33	0.047	10737021	497974
	Nin	ninein	-1.26	-0.33	0.044	10890402	299117
	Fabp6	fatty acid binding protein 6	-1.26	-0.33	0.024	10742200	25440
	Atg13	autophagy related 13	-1.26	-0.33	0.032	10847410	362164
	Hfm1	HFM1, ATP-dependent DNA helicase homolog	-1.26	-0.33	0.046	10771089	690161
	Son	Son DNA binding protein	-1.26	-0.33	0.023	10750219	304092
	Zfp451	zinc finger protein 451	-1.26	-0.33	0.028	10927233	316312
	Slc4a4	solute carrier family 4 member 4	-1.26	-0.33	0.049	10775997	84484
	Pbrm1	polybromo 1	-1.26	-0.33	0.035	10786590	306254
	Plekhg3	pleckstrin homology and RhoGEF domain containing G3	-1.26	-0.33	0.037	10885417	314249
	Slc23a2	solute carrier family 23 member 2	-1.26	-0.33	0.004	10850104	50622
	Rai1	retinoic acid induced 1	-1.26	-0.33	0.038	10734036	303188
	Dcx	doublecortin	-1.25	-0.33	0.001	10932746	84394
	Dock1	dedicator of cyto-kinesis 1	-1.25	-0.33	0.043	10711791	309081
	Pkd1l1	polycystin 1 like 1, transient receptor potential channel interacting	-1.25	-0.33	0.037	10774151	289796
	Adarb1	adenosine deaminase, RNA-specific, B1	-1.25	-0.33	0.040	10829367	25367
	Sgk494	uncharacterized serine/threonine-protein kinase SgK494	-1.25	-0.32	0.028	10745136	685208
	Cntf	ciliary neurotrophic factor	-1.25	-0.32	0.027	10729024	25707
	Supt6h	SPT6 homolog, histone chaperone	-1.25	-0.32	0.033	10736195	303281
	LOC102549548	uncharacterized LOC102549548	-1.25	-0.32	0.009	10860617	102549548
	Brd2	bromodomain containing 2	-1.25	-0.32	0.018	10828364	294276
	Tns3	tensin 3	-1.25	-0.32	0.029	10778404	360980
Upregulated	*Adck1*	aarF domain containing kinase 1	1.25	0.32	0.004	10886198	366698
	LOC310177	similar to RIKEN cDNA 0610040D20	1.25	0.32	0.038	10822078	310177
	Perm1	PPARGC1 and ESRR induced regulator, muscle 1	1.25	0.32	0.042	10874860	313776
	Comtd1	catechol-O-methyltransferase domain containing 1	1.25	0.32	0.040	10778997	305685
	Me1	malic enzyme 1	1.25	0.32	0.012	10919103	24552
	Galk2	galactokinase 2	1.25	0.32	0.013	10839423	296117
	Endog	endonuclease G	1.25	0.33	0.048	10835150	362100
	Ap2s1	adaptor-related protein complex 2, sigma 1 subunit	1.25	0.33	0.049	10704499	65046
	Lias	lipoic acid synthetase	1.25	0.33	0.020	10776811	305348
	Trappc2b	trafficking protein particle complex 2B	1.25	0.33	0.033	10742507	100910318
	Pdf	peptide deformylase (mitochondrial)	1.25	0.33	0.006	10810836	690214
	Echdc2	enoyl CoA hydratase domain containing 2	1.26	0.33	0.029	10870762	298381
	Trappc4	trafficking protein particle complex 4	1.26	0.33	0.020	10916785	367073
	Ubac1	UBA domain containing 1	1.26	0.33	0.028	10843634	362087
	Fuca1	fucosidase, alpha-L- 1, tissue	1.26	0.33	0.033	10872940	24375
	Pfdn6	prefoldin subunit 6	1.26	0.33	0.022	10828442	309629
	Gramd2	GRAM domain containing 2	1.26	0.33	0.018	10910562	300761
	Hck	HCK proto-oncogene, Src family tyrosine kinase	1.26	0.33	0.036	10840975	25734
	Fsip1	fibrous sheath interacting protein 1	1.26	0.33	0.046	10848416	296074
	Mal2	mal, T-cell differentiation protein 2	1.26	0.33	0.016	10896533	362911
	Shmt2	serine hydroxymethyltransferase 2	1.26	0.33	0.031	10903061	299857
	Klhl7	kelch-like family member 7	1.26	0.33	0.015	10860111	362303
	Fam96a	family with sequence similarity 96, member A	1.26	0.33	0.048	10911042	300797
	Mrpl15	mitochondrial ribosomal protein L15	1.26	0.34	0.044	10867398	297799
	LOC100359928	microtubule-associated protein 1 light chain 3 beta-like	1.26	0.34	0.041	10792602	100359928

Continuation Table S3.

Gene Symbol	Gene Description	Fold Change	Log Fold Change	p Value	Affymetrix ID	Entrez Gene ID
Trip10	thyroid hormone receptor interactor 10	1.26	0.34	0.038	10931182	116717
Klrc3	killer cell lectin-like receptor subfamily C, member 3	1.26	0.34	0.006	10866052	500338
Dtwd1	DTW domain containing 1	1.26	0.34	0.021	10839438	296119
Me3	malic enzyme 3	1.27	0.34	0.004	10708544	361602
Mrps27	mitochondrial ribosomal protein S27	1.27	0.34	0.044	10812722	361883
Rabggtb	Rab geranylgeranyltransferase, beta subunit	1.27	0.34	0.023	10827438	25533
Rexo4	REX4 homolog, 3'-5' exonuclease	1.27	0.34	0.002	10843909	311826
Gnpnat1	glucosamine-phosphate N-acetyltransferase 1	1.27	0.34	0.045	10782846	498486
LOC500028	hypothetical protein LOC500028	1.27	0.34	0.040	10861026	500028
Acy1	aminoacylase 1	1.27	0.34	0.048	10919897	300981
Polr2h	polymerase (RNA) II	1.27	0.34	0.029	10755309	498109
Mtrf1l	mitochondrial translational release factor 1-like	1.27	0.34	0.035	10717785	361473
Msra	methionine sulfoxide reductase A	1.27	0.35	0.015	10784517	29447
Slc39a8	solute carrier family 39 member 8	1.27	0.35	0.032	10819269	295455
Wfdc21	WAP four-disulfide core domain 21	1.27	0.35	0.036	10736875	360228
Rexo2	RNA exonuclease 2	1.27	0.35	0.044	10917095	300689
Klhl25	kelch-like family member 25	1.27	0.35	0.032	10707992	293023
Grhpr	glyoxylate and hydroxypyruvate reductase	1.28	0.35	0.033	10868673	680021
Acads	acyl-CoA dehydrogenase, C-2 to C-3 short chain	1.28	0.35	0.032	10759034	64304
Hibch	3-hydroxyisobutyryl-CoA hydrolase	1.28	0.35	0.040	10927809	301384
RGD1560961	similar to RIKEN cDNA 0610038L10 gene	1.28	0.35	0.046	10847718	311241
Mgst2	microsomal glutathione S-transferase 2	1.28	0.36	0.049	10815308	295037
Fech	ferrochelatase	1.28	0.36	0.031	10804875	361338
Them4	thioesterase superfamily member 4	1.28	0.36	0.028	10817208	361992
LOC681338	hypothetical protein LOC681338	1.28	0.36	0.040	10928032	---
Rars	arginyl-tRNA synthetase	1.28	0.36	0.012	10742033	287191
Eci3	enoyl-Coenzyme A delta isomerase 3	1.28	0.36	0.031	10794779	291076
Hexb	hexosaminidase subunit beta	1.28	0.36	0.011	10820693	294673
Ndufb8	NADH:ubiquinone oxidoreductase subunit B8	1.28	0.36	0.048	10730383	293991
Yae1d1	Yae1 domain containing 1	1.28	0.36	0.047	10795418	306994
Eef1b2	eukaryotic translation elongation factor 1 beta 2	1.28	0.36	0.039	10923857	363241
Nqo2	NAD (P)H quinone dehydrogenase 2	1.28	0.36	0.042	10798119	291084
RGD1559808	similar to 40S ribosomal protein S26	1.28	0.36	0.028	10921270	316158
Dcaf4	DDB1 and CUL4 associated factor 4	1.28	0.36	0.024	10885788	362762
RragB	Ras-related GTP binding B	1.28	0.36	0.036	10937479	117043
Iah1	isoamyl acetate-hydrolyzing esterase 1 homolog	1.28	0.36	0.035	10883858	298917
Phb-ps1	prohibitin, pseudogene 1	1.28	0.36	0.027	10745551	287559
Erp44	endoplasmic reticulum protein 44	1.28	0.36	0.038	10876675	298066
Ckb	creatine kinase B	1.28	0.36	0.014	10892265	24264
Kdelr3	KDEL endoplasmic reticulum protein retention receptor 3	1.28	0.36	0.031	10905453	315131
Mrps36	mitochondrial ribosomal protein S36	1.29	0.36	0.035	10821004	294696
Thnsl1	threonine synthase-like 1	1.29	0.36	0.019	10796696	498805
Bnip1	BCL2/adenovirus E1B interacting protein 1	1.29	0.36	0.041	10741826	140932
Gdf15	growth differentiation factor 15	1.29	0.36	0.016	10787517	29455
Pomp	proteasome maturation protein	1.29	0.37	0.026	10760041	288455
Lrrc57	leucine rich repeat containing 57	1.29	0.37	0.024	10848852	311346
Eif4e3	eukaryotic translation initiation factor 4E family member 3	1.29	0.37	0.037	10864425	297481

Continuation Table S3.

Gene Symbol	Gene Description	Fold Change	Log Fold Change	p Value	Affymetrix ID	Entrez Gene ID
Hmox2	heme oxygenase 2	1.29	0.37	0.042	10740681	79239
LOC690182	similar to Egl nine homolog 1 (Hypoxia-inducible factor prolyl hydroxylase 2)	1.29	0.37	0.039	10724578	690182
Nudt12	nudix hydrolase 12	1.29	0.37	0.011	10930190	367323
Mrpl45	mitochondrial ribosomal protein L45	1.29	0.37	0.043	10737815	287656
Ptcd2	pentatricopeptide repeat domain 2	1.29	0.37	0.010	10820824	310025
RGD1562079	RGD1562079	1.29	0.37	0.049	10720557	499125
Nudt5	nudix hydrolase 5	1.29	0.37	0.041	10799539	361274
Vamp4	vesicle-associated membrane protein 4	1.29	0.37	0.027	10765115	364033
LOC103692338	putative thymosin beta-4-like protein 6	1.29	0.37	0.005	10876291	103692338
Cnih4	cornichon family AMPA receptor auxiliary protein 4	1.29	0.37	0.047	10766289	289324
Tmem60	transmembrane protein 60	1.29	0.37	0.004	10860269	296761
Hmces	5-hydroxymethylcytosine (hmC) binding, ES cell-specific	1.29	0.37	0.048	10857107	500251
March2	membrane associated ring-CH-type finger 2	1.29	0.37	0.014	10901152	362849
Trim7	tripartite motif-containing 7	1.29	0.37	0.034	10733049	303089
Gstt2	glutathione S-transferase, theta 2	1.30	0.37	0.028	10829653	29487
Pter	phosphotriesterase related	1.30	0.37	0.001	10796440	63852
Spcs3	signal peptidase complex subunit 3	1.30	0.37	0.032	10788053	680782
Spryd7	SPRY domain containing 7	1.30	0.37	0.047	10784378	290303
Sirt5	sirtuin 5	1.30	0.37	0.010	10797827	306840
Atp6v1d	ATPase H+ transporting V1 subunit D	1.30	0.37	0.008	10890759	299159
Il33	interleukin 33	1.30	0.37	0.022	10714745	361749
Mcat	malonyl-CoA-acyl carrier protein transacylase	1.30	0.38	0.008	10898160	315173
Cisd3	CDGSH iron sulfur domain 3	1.30	0.38	0.050	10737866	287661
Ggct	gamma-glutamyl cyclotransferase	1.30	0.38	0.044	10855676	362368
Amacr	alpha-methylacyl-CoA racemase	1.30	0.38	0.038	10813678	25284
Ufsp2	UFM1-specific peptidase 2	1.31	0.39	0.026	10791719	361151
Klhdc1	kelch domain containing 1	1.31	0.39	0.045	10884934	314190
Casq1	calsequestrin 1	1.31	0.39	0.021	10769959	686019
Rab20	RAB20, member RAS oncogene family	1.31	0.39	0.041	10789591	689377
Dbi	diazepam binding inhibitor, acyl-CoA binding protein	1.31	0.39	0.035	10931154	25045
LOC688815	similar to prohibitin	1.31	0.39	0.043	10805784	688815
Sec61b	Sec61 translocon beta subunit	1.31	0.39	0.050	10868935	298068
Nudt7	nudix hydrolase 7	1.31	0.39	0.018	10808159	361413
Nmnat1	nicotinamide nucleotide adenylyltransferase 1	1.32	0.40	0.007	10881771	298653
Coq6	coenzyme Q6 monooxygenase	1.32	0.40	0.034	10885918	299195
Mrpl19	mitochondrial ribosomal protein L19	1.32	0.40	0.046	10863420	297372
Chfr	checkpoint with forkhead and ring finger domains	1.32	0.40	0.043	10763116	288734
Krt8	keratin 8	1.32	0.40	0.031	10751352	25626
Mgst3	microsomal glutathione S-transferase 3	1.32	0.40	0.029	10769629	289197
Hsd17b8	hydroxysteroid (17-beta) dehydrogenase 8	1.32	0.40	0.042	10828394	361802
RGD1306063	similar to HT021	1.32	0.40	0.027	10782599	289928
Bckdhb	branched chain keto acid dehydrogenase E1 subunit beta	1.32	0.40	0.031	10911976	29711
Cystm1	cysteine-rich transmembrane module containing 1	1.32	0.40	0.035	10800993	100363310
Fam220a	family with sequence similarity 220, member A	1.32	0.40	0.009	10760393	498145
Slc15a4	solute carrier family 15 member 4	1.32	0.41	0.020	10758094	246280
LOC685505	similar to coiled-coil-helix-coiled-coil-helix domain containing 4	1.32	0.41	0.029	10923617	685505
Hccs	holocytochrome c synthase	1.33	0.41	0.040	10933279	317444

Continuation Table S3.

Gene Symbol	Gene Description	Fold Change	Log Fold Change	p Value	Affymetrix ID	Entrez Gene ID
Sec11c	SEC11 homolog C, signal peptidase complex subunit	1.33	0.41	0.042	10802324	266758
Rtp4	receptor (chemosensory) transporter protein 4	1.33	0.41	0.038	10755088	360733
Plpp4	phospholipid phosphatase 4	1.33	0.41	0.043	10711454	309014
Mlx	MLX, MAX dimerization protein	1.33	0.41	0.049	10738284	360631
Mmachc	methylmalonic aciduria (cobalamin deficiency) cblC type, with homocystinuria	1.33	0.41	0.021	10878905	313520
Hikeshi/l7Rn6	Hikeshi, Heat Shock Protein Nuclear Import Factor	1.33	0.41	0.044	10723601	51501
LOC102554194	zinc finger protein 709-like	1.33	0.42	0.025	10926095	102554194
Mpi	mannose phosphate isomerase (mapped)	1.33	0.42	0.022	10917770	300741
Cmc1	C-x (9)-C motif containing 1	1.33	0.42	0.041	10920797	363162
Cstb	cystatin B	1.34	0.42	0.034	10832228	25308
Ift20	intraflagellar transport 20	1.34	0.42	0.041	10736300	287541
Ccdc159	coiled-coil domain containing 159	1.34	0.42	0.021	10908549	300442
Dus4l	dihydrouridine synthase 4-like	1.34	0.42	0.042	10889568	366593
Msln	mesothelin	1.34	0.42	0.040	10741486	60333
Clec2d2	C-type lectin domain family 2 member D2	1.34	0.42	0.022	10865956	362445
Rgs16	regulator of G-protein signaling 16	1.34	0.42	0.023	10764773	360857
Trmt1l	tRNA methyltransferase 1-like	1.34	0.42	0.043	10764643	304851
Cd53	Cd53 molecule	1.34	0.43	0.023	10825809	24251
Dus2	dihydrouridine synthase 2	1.34	0.43	0.046	10807435	291978
Ddit4l	DNA-damage-inducible transcript 4-like	1.34	0.43	0.034	10819318	100363484
Atp6v1g2	ATPase H+ transporting V1 subunit G2	1.34	0.43	0.013	10831152	368044
Tmem177	transmembrane protein 177	1.35	0.43	0.036	10767162	304735
Krt19	keratin 19	1.35	0.43	0.041	10747262	360626
Chchd4	coiled-coil-helix-coiled-coil-helix domain containing 4	1.35	0.43	0.032	10864100	312559
Rps27	ribosomal protein S27	1.35	0.43	0.021	10824596	94266
Dnajb9	DnaJ heat shock protein family (Hsp40) member B9	1.35	0.43	0.047	10889766	24908
Coq9	coenzyme Q9	1.35	0.43	0.046	10809251	498909
Gm8444	Uncharacterized protein; Predicted gene 8444 (118 aa)	1.35	0.44	0.028	10932893	---
Plin5	perilipin 5	1.35	0.44	0.027	10931038	501283
Atp6v0e2	ATPase, H+ transporting V0 subunit e2	1.36	0.44	0.023	10855356	436582
Lsm10	LSM10, U7 small nuclear RNA associated	1.36	0.44	0.037	10872000	366468
Upk1b	uroplakin 1B	1.36	0.44	0.004	10751239	303924
LOC100361898	coiled-coil-helix-coiled-coil-helix domain containing 4	1.36	0.44	0.029	10714780	100361898
Dnajc28	DnaJ heat shock protein family (Hsp40) member C28	1.36	0.44	0.015	10753143	360699
Tango2	transport and golgi organization 2 homolog	1.36	0.45	0.050	10752309	360738
Mkks	McKusick-Kaufman syndrome	1.36	0.45	0.045	10850221	311456
Bag1	Bcl2 associated athanogene 1	1.36	0.45	0.050	10876052	297994
LOC690384	similar to ribosomal protein L31	1.36	0.45	0.048	10779907	690384
Mrps16	mitochondrial ribosomal protein S16	1.36	0.45	0.020	10779159	688912
Snrpd2	small nuclear ribonucleoprotein D2 polypeptide	1.37	0.45	0.043	10704755	680309
Atp5s	ATP synthase, H+ transporting, mitochondrial Fo complex, subunit s (factor B)	1.37	0.45	0.033	10884964	362749
Nkg7	natural killer cell granule protein 7	1.37	0.45	0.045	10706326	171062
LOC103693118	60S ribosomal protein L37-like	1.37	0.45	0.035	10910722	103693118
Fam69b	family with sequence similarity 69, member B	1.37	0.45	0.019	10834462	362090
Smco1	single-pass membrane protein with coiled-coil domains 1	1.37	0.46	0.023	10754857	498096
LOC102548740	uncharacterized LOC102548740	1.37	0.46	0.020	10840318	102548740
Glcci1	glucocorticoid induced 1	1.37	0.46	0.040	10853707	296884

Continuation Table S3.

Gene Symbol	Gene Description	Fold Change	Log Fold Change	p Value	Affymetrix ID	Entrez Gene ID
Mrpl40	mitochondrial ribosomal protein L40	1.37	0.46	0.005	10755987	287962
Pla2g5	phospholipase A2, group V	1.37	0.46	0.020	10880872	29354
Coq10a	coenzyme Q10A	1.37	0.46	0.027	10899756	362810
Nme6	NME/NM23 nucleoside diphosphate kinase 6	1.38	0.46	0.019	10913545	58964
Dhrs11	dehydrogenase/reductase 11	1.38	0.46	0.027	10745757	360583
Enkur	enkurin, TRPC channel interacting protein	1.38	0.46	0.005	10799977	291354
LOC684557	similar to mitochondrial ribosomal protein L40	1.38	0.46	0.004	10781424	684557
Creld2	cysteine-rich with EGF-like domains 2	1.38	0.47	0.020	10898456	362978
Rgs18	regulator of G-protein signaling 18	1.38	0.47	0.042	10768357	289076
Ndufa2	NADH:ubiquinone oxidoreductase subunit A2	1.38	0.47	0.047	10803995	291660
Insig2	insulin induced gene 2	1.38	0.47	0.016	10767175	288985
Pde6d	phosphodiesterase 6D	1.39	0.47	0.014	10929600	363272
Ap1s2	adaptor-related protein complex 1, sigma 2 subunit	1.39	0.47	0.048	10937769	302671
Adpgk	ADP-dependent glucokinase	1.39	0.47	0.010	10910482	315722
Zfp605	zinc finger protein 605	1.39	0.47	0.023	10763137	100365779
Nmnat3	nicotinamide nucleotide adenylyltransferase 3	1.39	0.48	0.033	10912433	363118
Rbp7	retinol binding protein 7	1.39	0.48	0.032	10881766	362662
Gli4	GLI family zinc finger 4	1.39	0.48	0.022	10897070	500893
Nqo1	NAD (P)H quinone dehydrogenase 1	1.39	0.48	0.048	10810867	24314
Fbxo44	F-box protein 44	1.40	0.48	0.002	10881590	500587
Zcchc4	zinc finger CCHC-type containing 4	1.40	0.48	0.011	10777081	360946
Ruvbl2	RuvB-like AAA ATPase 2	1.40	0.48	0.033	10721812	292907
Mycbp	Myc binding protein	1.40	0.48	0.039	10871785	100361133
Abhd14a	abhydrolase domain containing 14A	1.40	0.48	0.037	10919912	300982
Hddc3	HD domain containing 3	1.40	0.49	0.018	10708230	308758
Unc119	unc-119 lipid binding chaperone	1.40	0.49	0.012	10745074	29402
Lilrb4	leukocyte immunoglobulin like receptor B4	1.41	0.49	0.048	10718954	292594
Cmbl	carboxymethylenebutenolidase homolog	1.41	0.49	0.023	10814105	310201
Mrps35	mitochondrial ribosomal protein S35	1.41	0.49	0.015	10859725	297727
Tmem160	transmembrane protein 160	1.41	0.50	0.029	10704482	292654
Cby1	chibby family member 1, beta catenin antagonist	1.41	0.50	0.046	10897698	246768
Asb18	ankyrin repeat and SOCS box-containing 18	1.41	0.50	0.011	10929732	316614
Acot13	acyl-CoA thioesterase 13	1.42	0.50	0.037	10795108	291135
Cd200r1	CD200 receptor 1	1.42	0.50	0.040	10754000	64357
L2hgdh	L-2-hydroxyglutarate dehydrogenase	1.42	0.51	0.006	10890342	314196
Arl2	ADP-ribosylation factor like GTPase 2	1.42	0.51	0.049	10728159	65142
Asb11	ankyrin repeat and SOCS box containing 11	1.42	0.51	0.033	10937709	302666
Fam96b	family with sequence similarity 96, member B	1.42	0.51	0.028	10805640	680987
Akr1e2	aldo-keto reductase family 1, member E2	1.43	0.52	0.026	10796027	307091
Bphl	biphenyl hydrolase like	1.43	0.52	0.032	10798100	361239
Tmem242	transmembrane protein 242	1.43	0.52	0.010	10717855	292228
Lrrc14b	leucine rich repeat containing 14B	1.44	0.53	0.029	10702450	502225
Mterf4	mitochondrial transcription termination factor 4	1.44	0.53	0.018	10929991	363289
RGD1561381	similar to microsomal glutathione S-transferase 3	1.44	0.53	0.029	10771881	498340
Asb15	ankyrin repeat and SOCS box containing 15	1.45	0.53	0.003	10853995	500050
Dguok	deoxyguanosine kinase	1.45	0.53	0.008	10863542	297389
Hpdl	4-hydroxyphenylpyruvate dioxygenase-like	1.46	0.55	0.039	10878920	313521

Continuation Table S3.

Gene Symbol	Gene Description	Fold Change	Log Fold Change	p Value	Affymetrix ID	Entrez Gene ID
Adm	adrenomedullin	1.46	0.55	0.021	10709875	25026
Yars2	tyrosyl-tRNA synthetase 2	1.46	0.55	0.042	10752592	287924
Tpmt	thiopurine S-methyltransferase	1.46	0.55	0.018	10794464	690050
Sdhaf3	succinate dehydrogenase complex assembly factor 3	1.47	0.56	0.015	10853676	362323
Cd74	CD74 molecule	1.47	0.56	0.037	10802013	25599
Gcat	glycine C-acetyltransferase	1.47	0.56	0.033	10897574	366959
Gadd45g	growth arrest and DNA-damage-inducible, gamma	1.47	0.56	0.011	10797527	291005
Dlx5	distal-less homeobox 5	1.47	0.56	0.023	10860947	25431
Cyb5d2	cytochrome b5 domain containing 2	1.48	0.56	0.038	10744751	303293
Ptges3l	prostaglandin E synthase 3 like	1.48	0.57	0.020	10747550	103693432
Eif3i-ps1	eukaryotic translation initiation factor 3, subunit I, pseudogene 1	1.50	0.58	0.026	10815500	499619
Nif3l1	NGG1 interacting factor 3 like 1	1.50	0.58	0.025	10923567	301431
Plpp7	phospholipid phosphatase 7	1.50	0.59	0.015	10835498	296635
Tmem206	transmembrane protein 206	1.50	0.59	0.012	10766726	305070
Myl1	myosin, light chain 1	1.51	0.59	0.016	10928614	56781
Dcun1d2	defective in cullin neddylation 1 domain containing 2	1.51	0.59	0.044	10789470	688913
Prdx5	peroxiredoxin 5	1.51	0.60	0.037	10728240	113898
Banp	Btg3 associated nuclear protein	1.52	0.60	0.015	10808461	292064
Nmu	neuromedin U	1.52	0.61	0.028	10772231	63887
Mrps18b	mitochondrial ribosomal protein S18B	1.53	0.62	0.040	10827870	294230
Gstk1	glutathione S-transferase kappa 1	1.54	0.62	0.048	10855008	297029
Mcee	methylmalonyl CoA epimerase	1.54	0.63	0.034	10707721	293829
Mcrip2	MAPK Regulated Corepressor Interacting Protein 2	1.55	0.63	0.046	10741629	84331
Cptp	ceramide-1-phosphate transfer protein	1.55	0.63	0.033	10882234	313771
Pam16	presequence translocase associated motor 16 homolog	1.56	0.64	0.027	10731783	679907
Lsmem2	leucine-rich single-pass membrane protein 2	1.56	0.64	0.023	10920084	689621
Isoc2b	isochorismatase domain containing 2b	1.56	0.64	0.039	10703930	361501
Prosc	proline synthetase co-transcribed homolog (bacterial)	1.56	0.65	0.037	10792187	306544
Trim50	tripartite motif-containing 50	1.58	0.66	0.037	10761201	288596
RGD1559955	similar to 40S ribosomal protein S17	1.58	0.66	0.025	10925757	367324
Mtfp1	mitochondrial fission process 1	1.60	0.67	0.048	10778038	289745
Rp1	retinitis pigmentosa 1	1.60	0.68	0.009	10867411	681377
Blvrb	biliverdin reductase B	1.62	0.70	0.019	10705358	292737
Stc1	stanniocalcin 1	1.65	0.72	0.016	10781273	81801
LOC298139	similar to RIKEN cDNA 2310003M01	1.69	0.76	0.028	10877669	298139
LOC500300	similar to hypothetical protein MGC6835	1.72	0.78	0.032	10858160	500300
LOC100910173	mitochondrial thiamine pyrophosphate carrier-like	1.77	0.82	0.020	10748999	100910173
LOC103692294	uncharacterized LOC103692294	1.77	0.83	0.005	10867423	103692294
Sctr	secretin receptor	1.78	0.84	0.024	10931678	81779
Edn1	endothelin 1	1.79	0.84	0.005	10797857	24323
Reg3g	regenerating family member 3 gamma	1.90	0.93	0.047	10863410	24620
Bdh1	3-hydroxybutyrate dehydrogenase, type 1	2.03	1.02	0.046	10754931	117099
Kcne1	potassium voltage-gated channel subfamily E regulatory subunit 1	2.15	1.11	0.042	10753211	25471

Table S4.
Differentially expressed genes in Diabetes exposed vs Control group.

	Gene Symbol	Gene Description	Fold Change	Log Fold Change	p Value	Affymetrix ID	Entrez Gene ID
Downregulated	*Drd4*	dopamine receptor D4	-1.37	-0.46	0.046	10712265	25432
	Magi3	membrane associated guanylate kinase, WW and PDZ domain containing 3	-1.33	-0.41	0.033	10825609	245903
	RGD1560775	similar to RIKEN cDNA 4930579C12 gene	-1.28	-0.36	0.007	10919231	501031
	LOC690235	similar to MAP/microtubule affinity-regulating kinase 4	-1.28	-0.35	0.021	10915165	690235
Upregulated	*Ahsp*	alpha hemoglobin stabilizing protein	2.00	1.00	0.027	10711396	293522
	Car1	carbonic anhydrase I	1.60	0.68	0.050	10814184	310218
	Frzb	frizzled-related protein	1.48	0.56	0.013	10846740	295691
	Kel	Kell blood group, metallo-endopeptidase	1.48	0.56	0.050	10862234	297025
	Fbln5	fibulin 5	1.46	0.54	0.025	10891780	29158
	Olr1566	olfactory receptor 1566	1.36	0.45	0.001	10752233	287973
	Vangl1	VANGL planar cell polarity protein 1	1.34	0.42	0.050	10825510	690366
	Rhag	Rh-associated glycoprotein	1.31	0.39	0.033	10926769	65207
	Acan	aggrecan	1.29	0.37	0.041	10708021	58968
	Olr1528	olfactory receptor 1528	1.29	0.36	0.020	10750606	405995
	Itga8	integrin subunit alpha 8	1.29	0.36	0.037	10799684	364786
	Polr3k	RNA polymerase III subunit K	1.28	0.36	0.003	10843132	366277
	Lrrtm3	leucine rich repeat transmembrane neuronal 3	1.27	0.35	0.023	10832917	294380
	Slc7a3	solute carrier family 7 member 3	1.27	0.35	0.015	10938635	29485
	Ntrk3	neurotrophic receptor tyrosine kinase 3	1.26	0.34	0.038	10722864	29613

Table S5.
Differentially expressed genes in High-Fat diet exposed vs Control group.

	Gene Symbol	Gene Description	Fold Change	Log Fold Change	p Value	Affymetrix ID	Entrez Gene ID
Downregulated	*Reg3g*	regenerating family member 3 gamma	-2.10	-1.07	0.001	10863410	24620
	Reg3b	regenerating family member 3 beta	-1.99	-0.99	0.033	10856474	24618
	Pik3ip1	phosphoinositide-3-kinase interacting protein 1	-1.51	-0.60	0.022	10773695	305472
	Ddit4l	DNA-damage-inducible transcript 4-like	-1.47	-0.55	0.006	10819318	100363484
	Lrrc14b	leucine rich repeat containing 14B	-1.47	-0.55	0.023	10702450	502225
	RT1-T24-2	RT1 class I, locus T24, gene 2	-1.44	-0.53	0.036	10827830	415057
	Klhl33	kelch-like family member 33	-1.42	-0.51	0.011	10783140	100362676
	LOC102553195	lipoxygenase homology domain-containing protein 1-like	-1.40	-0.49	0.022	10867423	102553195
	Rgs18	regulator of G-protein signaling 18	-1.40	-0.48	0.035	10768357	289076
	Klhl38	kelch-like family member 38	-1.39	-0.47	0.049	10903906	314996
	Sema7a	semaphorin 7A, GPI membrane anchor	-1.37	-0.45	0.012	10910406	315711
	Fbxo32	F-box protein 32	-1.36	-0.44	0.035	10903896	171043
	Adm	adrenomedullin	-1.36	-0.44	0.023	10709875	25026
	Plekhf1	pleckstrin homology and FYVE domain containing 1	-1.35	-0.43	0.011	10721188	308543
	LOC500300	similar to hypothetical protein MGC6835	-1.34	-0.43	0.036	10858160	500300
	Rgs2	regulator of G-protein signaling 2	-1.34	-0.42	0.013	10768332	84583
	Myom2	myomesin 2	-1.34	-0.42	0.047	10792621	306616
	Lilrb4	leukocyte immunoglobulin like receptor B4	-1.33	-0.41	0.047	10718954	292594
	Gpr183	G protein-coupled receptor 183	-1.31	-0.38	0.048	10786028	679975
	Rtp4	receptor (chemosensory) transporter protein 4	-1.30	-0.38	0.044	10755088	360733
	Nr1d1	nuclear receptor subfamily 1, group D, member 1	-1.30	-0.38	0.044	10746955	252917
	Gpnmb	glycoprotein nmb	-1.29	-0.37	0.034	10855449	113955
	Tmem267	transmembrane protein 267	-1.29	-0.36	0.031	10813206	64417
	Adgrv1	adhesion G protein-coupled receptor V1	-1.28	-0.36	0.005	10820145	100362255
	Fam227a	family with sequence similarity 227, member A	-1.27	-0.35	0.036	10897648	300074
	Fuz	fuzzy planar cell polarity protein	-1.27	-0.34	0.018	10706668	308577
	Gbp2	guanylate binding protein 2	-1.26	-0.34	0.023	10819523	171164
	Fsip1	fibrous sheath interacting protein 1	-1.26	-0.34	0.036	10848416	296074
	RGD1561916	similar to testes development-related NYD-SP22 isoform 1	-1.26	-0.33	0.034	10876149	500441
	Bco2	beta-carotene oxygenase 2	-1.26	-0.33	0.009	10917215	315644
Upregulated	*Hbe1*	hemoglobin subunit epsilon 1	1.82	0.86	0.034	10724331	293267
	Mir323	microRNA 323	1.69	0.76	0.025	10887056	100313969
	Rnd1	Rho family GTPase 1	1.65	0.72	0.013	10906926	362993
	Mir539	microRNA 539	1.52	0.60	0.022	10887086	100314167
	Mir377	microRNA 377	1.51	0.59	0.019	10887100	100314283
	Mir382	microRNA 382	1.51	0.59	0.042	10887090	100314085
	Fbln5	fibulin 5	1.49	0.57	0.040	10891780	29158
	Tnfrsf12a	TNF receptor superfamily member 12A	1.47	0.56	0.008	10740869	302965

Continuation Table S5.

Gene Symbol	Gene Description	Fold Change	Log Fold Change	p Value	Affymetrix ID	Entrez Gene ID
Hbegf	heparin-binding EGF-like growth factor	1.46	0.55	0.027	10803947	25433
Mir27b	microRNA 27b	1.44	0.52	0.045	10796987	100314005
Pcdh18	protocadherin 18	1.41	0.50	0.005	10823052	295027
Mirlet7f-1	microRNA let-7f-1	1.41	0.49	0.032	10794444	100314277
Vash2	vasohibin 2	1.39	0.47	0.014	10770702	498309
Iqgap3	IQ motif containing GTPase activating protein 3	1.37	0.46	0.003	10816433	310621
Mir15b	microRNA 15b	1.36	0.45	0.039	10815913	100314150
Acan	aggrecan	1.36	0.45	0.023	10708021	58968
Mir350	microRNA 350	1.35	0.43	0.005	10770195	100313985
RGD1562080	similar to Hypothetical protein CBG10141	1.34	0.43	0.009	10803440	498827
LOC103694255	uncharacterized LOC103694255	1.34	0.42	0.013	10805524	103694255
Dusp8	dual specificity phosphatase 8	1.33	0.41	0.026	10726929	361679
Crispld2	cysteine-rich secretory protein LCCL domain containing 2	1.31	0.39	0.026	10808377	171547
Lnc215	long non-coding RNA 215	1.31	0.39	0.043	10909377	104845260
Mllt11	myeloid/lymphoid or mixed-lineage leukemia; translocated to, 11	1.31	0.39	0.006	10824965	295264
Slc7a1	solute carrier family 7 member 1	1.30	0.38	0.001	10756393	25648
Stk38l	serine/threonine kinase 38 like	1.30	0.38	0.024	10859660	691337
Slfn13	schlafen family member 13	1.30	0.37	0.024	10745595	303378
Hivep1	human immunodeficiency virus type I enhancer binding protein 1	1.30	0.37	0.018	10797863	117140
Piezo2	piezo-type mechanosensitive ion channel component 2	1.29	0.36	0.044	10804821	307380
Il12rb2	interleukin 12 receptor subunit beta 2	1.28	0.36	0.016	10862876	171334
Etv6	ets variant 6	1.27	0.35	0.016	10859227	312777
Setbp1	SET binding protein 1	1.27	0.35	0.037	10805375	291423
Sacs	sacsin molecular chaperone	1.27	0.34	0.022	10780908	305940
Creb5	cAMP responsive element binding protein 5	1.27	0.34	0.023	10855601	500131
Kif4b	kinesin family member 4B	1.26	0.34	0.022	10886404	299255
Mir16	microRNA 16	1.26	0.34	0.029	10815915	100313997
Lrrtm3	leucine rich repeat transmembrane neuronal 3	1.26	0.33	0.005	10832917	294380
Rnd3	Rho family GTPase 3	1.26	0.33	0.039	10845124	295588
Ntrk2	neurotrophic receptor tyrosine kinase 2	1.26	0.33	0.041	10797089	25054
Tigd4	tigger transposable element derived 4	1.25	0.33	0.004	10816151	102550932
Chd7	chromodomain helicase DNA binding protein 7	1.25	0.33	0.046	10867539	312974
Fat4	FAT atypical cadherin 4	1.25	0.33	0.027	10815057	310341
Anln	anillin, actin binding protein	1.25	0.32	0.043	10799084	363031
Arhgap42	Rho GTPase activating protein 42	1.25	0.32	0.014	10914877	500943
Ltbp2	latent transforming growth factor beta binding protein 2	1.25	0.32	0.044	10891165	59106
Defb49	defensin beta 49	1.25	0.32	0.049	10926837	641653

Table S6.
Differentially expressed genes in Diabetes exposed vs High-Fat diet exposed group.

	Gene Symbol	Gene Description	Fold Change	Log Fold Change	p Value	Affymetrix ID	Entrez Gene ID
Downregulated	*Mcpt2*	mast cell protease 2	-1.70	-0.77	0.008	10780598	29266
	Pcdh18	protocadherin 18	-1.53	-0.62	0.002	10823052	295027
	Btn3a2	butyrophilin, subfamily 3, member A2	-1.47	-0.55	0.027	10828340	294268
	Snrpd1	small nuclear ribonucleoprotein D1	-1.43	-0.52	0.019	10800140	291794
	Sacs	sacsin molecular chaperone	-1.43	-0.52	0.002	10780908	305940
	Vcam1	vascular cell adhesion molecule 1	-1.41	-0.49	0.022	10826249	25361
	Spaca6/LOC688452	hypothetical protein, sperm acrosome associated 6	-1.37	-0.45	0.046	10703434	147650
	Crispld2	cysteine-rich secretory protein LCCL domain containing 2	-1.37	-0.45	0.015	10808377	171547
	Mllt11	myeloid/lymphoid or mixed-lineage leukemia; translocated to 11	-1.36	-0.44	0.001	10824965	295264
	Rcan1	regulator of calcineurin 1	-1.34	-0.42	0.005	10753214	266766
	Chd7	chromodomain helicase DNA binding protein 7	-1.33	-0.41	0.043	10867539	312974
	Tnfrsf12a	TNF receptor superfamily member 12A	-1.31	-0.39	0.049	10740869	302965
	Filip1	filamin A interacting protein 1	-1.31	-0.39	0.000	10918955	246776
	Fam212b	family with sequence similarity 212, member B	-1.29	-0.37	0.044	10818155	310764
	Zfp867	zinc finger protein 867	-1.29	-0.37	0.027	10743144	100125361
	Creb5	cAMP responsive element binding protein 5	-1.28	-0.35	0.046	10855601	500131
	Smim23	small integral membrane protein 23	-1.28	-0.35	0.013	10741902	360508
	Adprhl1	ADP-ribosylhydrolase like 1	-1.27	-0.34	0.018	10789500	290880
Upregulated	*Gypa*	glycophorin A	1.86	0.89	0.013	10810434	688972
	Ahsp	alpha hemoglobin stabilizing protein	1.80	0.84	0.011	10711396	293522
	Reg3g	regenerating family member 3 gamma	1.76	0.81	0.032	10863410	24620
	Car1	carbonic anhydrase I	1.72	0.78	0.020	10814184	310218
	Slc4a1	solute carrier family 4 (anion exchanger), member 1	1.63	0.70	0.048	10747813	24779
	Rhd	Rh blood group, D antigen	1.56	0.64	0.045	10872814	60414
	Pik3ip1	phosphoinositide-3-kinase interacting protein 1	1.54	0.63	0.011	10773695	305472
	Reg3b	regenerating family member 3 beta	1.49	0.58	0.018	10856474	24618
	RT1-T24-2	RT1 class I, locus T24, gene 2	1.47	0.56	0.027	10827830	415057
	Kel	Kell blood group, metallo-endopeptidase	1.44	0.53	0.035	10862234	297025
	Rhag	Rh-associated glycoprotein	1.42	0.51	0.012	10926769	65207
	Ddit4	DNA-damage-inducible transcript 4	1.41	0.50	0.050	10832920	140942
	Fbxo32	F-box protein 32	1.40	0.49	0.046	10903896	171043
	Lsmem1	leucine-rich single-pass membrane protein 1	1.39	0.47	0.049	10889753	680810
	Olr1528	olfactory receptor 1528	1.38	0.46	0.027	10750606	405995
	Plekhf1	pleckstrin homology and FYVE domain containing 1	1.30	0.38	0.008	10721188	308543
	Clec2d2	C-type lectin domain family 2 member D2	1.27	0.35	0.020	10865956	362445
	Fgf9	fibroblast growth factor 9	1.26	0.33	0.026	10780813	25444
	Pla2g7	phospholipase A2 group VII	1.26	0.33	0.030	10926683	301265
	Adgrv1	adhesion G protein-coupled receptor V1	1.25	0.33	0.008	10820145	100362255

Table S7.

Functional enrichment analysis in all up and down DEGs in Combination vs Control.

Annotation Cluster 1 Enrichment Score: 1.718

Category	Term	Count	PValue	Genes	Fold Enrichment	Bonferroni	Benjamini	FDR
REACTOME_PATHWAY	R-RNO-5389840:R-RNO-5389840: Mitochondrial translation elongation	6	0.003	MRPL40, MRPS27, MRPL19, MRPS10, MRPL38, DAP3	6.18	0.35	0.35	3.11
REACTOME_PATHWAY	R-RNO-5419276:R-RNO-5419276: Mitochondrial translation termination	6	0.003	MRPL40, MRPS27, MRPL19, MRPS10, MRPL38, DAP3	6.02	0.39	0.22	3.48
UP_KEYWORDS	Ribosomal protein	5	0.089	MRPL40, MRPS27, MRPL19, MRPS10, MRPL38	2.96	1.00	0.80	68.48
UP_KEYWORDS	Ribonucleoprotein	5	0.200	MRPL40, MRPS27, MRPL19, MRPS10, MRPL38	2.15	1.00	0.92	93.80

Annotation Cluster 2 Enrichment Score: 1.155

Category	Term	Count	PValue	Genes	Fold Enrichment	Bonferroni	Benjamini	FDR
UP_KEYWORDS	Myosin	3	0.064	MYO10, MYH3, MYL1	7.23	1.00	0.79	55.98
UP_KEYWORDS	Motor protein	4	0.069	MYO10, MYH3, DYNC2LI1, MYL1	4.21	1.00	0.78	58.84
GOTERM_CC_DIRECT	GO:0016459~myosin complex	3	0.078	MYO10, MYH3, MYL1	6.45	1.00	0.81	63.95

Annotation Cluster 3 Enrichment Score: 0.616

Category	Term	Count	PValue	Genes	Fold Enrichment	Bonferroni	Benjamini	FDR
UP_SEQ_FEATURE	domain:EGF-like 2	3	0.095	MEGF8, NOTCH2, HABP2	5.72	1.00	1.00	74.41
UP_SEQ_FEATURE	domain:EGF-like 1	3	0.149	MEGF8, NOTCH2, HABP2	4.34	1.00	1.00	89.05
SMART	SM00181:EGF	4	0.200	MEGF8, NOTCH2, CNTNAP3B, HABP2	2.57	1.00	1.00	91.92
UP_KEYWORDS	EGF-like domain	4	0.229	MEGF8, NOTCH2, C6, HABP2	2.41	1.00	0.92	96.04
SMART	SM00179:EGF_CA	3	0.293	MEGF8, NOTCH2, HABP2	2.75	1.00	1.00	98.01
INTERPRO	IPR001881:EGF-like calcium-binding	3	0.327	MEGF8, NOTCH2, HABP2	2.55	1.00	1.00	99.65
INTERPRO	IPR000742:Epidermal growth factor-like domain	4	0.351	MEGF8, NOTCH2, CNTNAP3B, HABP2	1.90	1.00	1.00	99.79
INTERPRO	IPR013032:EGF-like, conserved site	3	0.541	MEGF8, NOTCH2, HABP2	1.65	1.00	1.00	100.00

Annotation Cluster 4 Enrichment Score: 0.297

Category	Term	Count	PValue	Genes	Fold Enrichment	Bonferroni	Benjamini	FDR
SMART	SM00406:IGv	3	0.160	HAPLN1, MPZ, PAPLN	4.15	1.00	1.00	86.05
INTERPRO	IPR013106:Immunoglobulin V-set	3	0.480	HAPLN1, MPZ, PAPLN	1.85	1.00	1.00	99.99
SMART	SM00409:IG	4	0.633	FGFR2, HAPLN1, MPZ, PAPLN	1.22	1.00	1.00	100.00
INTERPRO	IPR003599:Immunoglobulin subtype	4	0.686	FGFR2, HAPLN1, MPZ, PAPLN	1.13	1.00	1.00	100.00
INTERPRO	IPR007110:Immunoglobulin-like domain	5	0.687	FGFR2, HAPLN1, MPZ, RT1-DB2, PAPLN	1.07	1.00	1.00	100.00
UP_KEYWORDS	Immunoglobulin domain	3	0.723	FGFR2, HAPLN1, MPZ	1.18	1.00	1.00	100.00

Annotation Cluster 5 Enrichment Score: 0.207

Category	Term	Count	PValue	Genes	Fold Enrichment	Bonferroni	Benjamini	FDR
SMART	SM00355:ZnF_C2H2	5	0.562	ZFP451, ZBTB34, ADNP, ZFP319, ZBTB1	1.25	1.00	1.00	99.99
INTERPRO	IPR015880:Zinc finger, C2H2-like	5	0.625	ZFP451, ZBTB34, ADNP, ZFP319, ZBTB1	1.16	1.00	1.00	100.00
INTERPRO	IPR007087:Zinc finger, C2H2	5	0.682	ZFP451, ZBTB34, ADNP, ZFP319, ZBTB1	1.08	1.00	1.00	100.00

Annotation Cluster 6 Enrichment Score: 0.139

Category	Term	Count	PValue	Genes	Fold Enrichment	Bonferroni	Benjamini	FDR
INTERPRO	IPR008271:Serine/threonine-protein kinase, active site	4	0.618	CDK1, TNIK, MYLK3, LATS1	1.25	1.00	1.00	100.00
SMART	SM00220:S_TKc	4	0.668	CDK1, TNIK, MYLK3, LATS1	1.16	1.00	1.00	100.00
GOTERM_BP_DIRECT	GO:0006468~protein phosphorylation	5	0.713	CDK1, TNIK, MYLK3, DGUOK, LATS1	1.03	1.00	1.00	100.00
INTERPRO	IPR000719:Protein kinase, catalytic domain	5	0.731	FGFR2, CDK1, TNIK, MYLK3, LATS1	1.01	1.00	1.00	100.00
INTERPRO	IPR017441:Protein kinase, ATP binding site	4	0.744	FGFR2, CDK1, TNIK, MYLK3	1.03	1.00	1.00	100.00
UP_KEYWORDS	Serine/threonine-protein kinase	3	0.764	CDK1, MYLK3, LATS1	1.09	1.00	1.00	100.00
INTERPRO	IPR011009:Protein kinase-like domain	5	0.787	FGFR2, CDK1, TNIK, MYLK3, LATS1	0.93	1.00	1.00	100.00
GOTERM_MF_DIRECT	GO:0004674~protein serine/threonine kinase activity	3	0.806	CDK1, TNIK, LATS1	0.99	1.00	1.00	100.00

Annotation Cluster 7 Enrichment Score: 0.088

Category	Term	Count	PValue	Genes	Fold Enrichment	Bonferroni	Benjamini	FDR
UP_KEYWORDS	Transcription	8	0.764	NOTCH2, EYA4, SAFB, ADNP, ESR1, TCEAL7, POLR2A, KMT5B	0.92	1.00	1.00	100.00
UP_KEYWORDS	Transcription regulation	7	0.830	NOTCH2, EYA4, SAFB, ADNP, ESR1, TCEAL7, KMT5B	0.85	1.00	1.00	100.00
GOTERM_BP_DIRECT	GO:0006351~transcription, DNA-templated	6	0.861	NOTCH2, SAFB, ADNP, ESR1, TCEAL7, KMT5B	0.82	1.00	1.00	100.00

Annotation Cluster 8 Enrichment Score: 0.003

Category	Term	Count	PValue	Genes	Fold Enrichment	Bonferroni	Benjamini	FDR
UP_KEYWORDS	Transmembrane helix	44	0.990	FGFR2, STEAP4, OSTC, MEGF8, STX8, SLC44A2, RT1-DB2, GABRB2, SLC22A15, DRD4, CYP2D1, CNTNAP3B, ABCA1, NFXL1, KCNMB1, SCTR, APLNR, GPR21, FAM171B, INSIG2, TECRL, MARVELD2, RAET1L, FAM162A, TRIP10, APOO, KDELR3, MPZ, VANGL1, LSMEM2, LIFR, SLCO2B1, PCDH17, ITPR3, SLC16A3, TMEM242, NOTCH2, CLEC12B, SPCS3, VAMP4, EQTN, SLC15A4, GRAMD1B, TM6SF1	0.77	1.00	1.00	100.00
UP_KEYWORDS	Transmembrane	44	0.991	FGFR2, STEAP4, OSTC, MEGF8, STX8, SLC44A2, RT1-DB2, GABRB2, SLC22A15, DRD4, CYP2D1, CNTNAP3B, ABCA1, NFXL1, KCNMB1, SCTR, APLNR, GPR21, FAM171B, INSIG2, TECRL, MARVELD2, RAET1L, FAM162A, TRIP10, APOO, KDELR3, MPZ, VANGL1, LSMEM2, LIFR, SLCO2B1, PCDH17, ITPR3, SLC16A3, TMEM242, NOTCH2, CLEC12B, SPCS3, VAMP4, EQTN, SLC15A4, GRAMD1B, TM6SF1	0.77	1.00	1.00	100.00
UP_KEYWORDS	Membrane	54	0.992	STEAP4, SLC44A2, GABRB2, RT1-DB2, SLC22A15, CYP2D1, NFXL1, SCTR, FAM171B, TECRL, INSIG2, RAET1L, APOO, KDELR3, MAGI3, VANGL1, LIFR, PDXP, ESR1, TMEM242, CLEC12B, PEBP1, VAMP4, EQTN, THEM4, CASQ1, TM6SF1, GRAMD1B, FGFR2, OSTC, MEGF8, STX8, DRD4, CNTNAP3B, ABCA1, KCNMB1, APLNR, GPR21, MARVELD2, FAM162A, CLVS1, TRIP10, BDH1, MPZ, LSMEM2, SLCO2B1, PCDH17, ITPR3, SLC16A3, NOTCH2, MYO10, PDE2A, SPCS3, SLC15A4	0.79	1.00	1.00	100.00
GOTERM_CC_DIRECT	GO:0016021~integral component of membrane	37	0.998	FGFR2, STEAP4, OSTC, MEGF8, STX8, SLC44A2, RT1-DB2, SLC22A15, CYP2D1, CNTNAP3B, NFXL1, KCNMB1, SCTR, APLNR, GPR21, FAM171B, TECRL, MARVELD2, RAET1L, FAM162A, TRIP10, KDELR3, VANGL1, LSMEM2, LIFR, SLCO2B1, PCDH17, ITPR3, TMEM242, L2HGDH, CLEC12B, SPCS3, VAMP4, EQTN, SLC15A4, GRAMD1B, TM6SF1	0.70	1.00	1.00	100.00

Annotation Cluster 9 Enrichment Score: 0.0000018

Category	Term	Count	PValue	Genes	Fold Enrichment	Bonferroni	Benjamini	FDR
UP_KEYWORDS	G-protein coupled receptor	4	1.000	APLNR, GPR21, DRD4, SCTR	0.25	1.00	1.00	100.00
UP_KEYWORDS	Transducer	4	1.000	APLNR, GPR21, DRD4, SCTR	0.24	1.00	1.00	100.00
INTERPRO	IPR000276:G protein-coupled receptor, rhodopsin-like	3	1.000	APLNR, GPR21, DRD4	0.20	1.00	1.00	100.00
INTERPRO	IPR017452:GPCR, rhodopsin-like, 7TM	3	1.000	APLNR, GPR21, DRD4	0.19	1.00	1.00	100.00

Table S8

Functional pathway enrichment analysis (Reactome) of up and downregulated DEGs in Combiunation vs Control.

Pathway identifier	Pathway name	#Entities found	Submitted entities found	#Entities total	Entities ratio	Entities pValue	Entities FDR
R-HSA-1839126	FGFR2 mutant receptor activation	8	Fgf7;Polr2a;Fgfr2	49	0.003	0.00000	0.002
R-HSA-8851708	Signaling by FGFR2 IIIa TM	6	Polr2a;Fgfr2	24	0.002	0.00001	0.002
R-HSA-5655253	Signaling by FGFR2 in disease	8	Fgf7;Polr2a;Fgfr2	64	0.004	0.00002	0.004
R-HSA-5654221	Phospholipase C-mediated cascade; FGFR2	5	Fgf7;Fgfr2	25	0.002	0.00009	0.014
R-HSA-190241	FGFR2 ligand binding and activation	5	Fgf7;Fgfr2	26	0.002	0.00011	0.014
R-HSA-5654695	PI-3K cascade:FGFR2	5	Fgf7;Fgfr2	31	0.002	0.00024	0.025
R-HSA-1226099	Signaling by FGFR in disease	8	Fgf7;Polr2a;Fgfr2	94	0.006	0.00029	0.025
R-HSA-5654699	SHC-mediated cascade:FGFR2	5	Fgf7;Fgfr2	33	0.002	0.00032	0.025
R-HSA-5654700	FRS-mediated FGFR2 signaling	5	Fgf7;Fgfr2	34	0.002	0.00037	0.026
R-HSA-2219530	Constitutive Signaling by Aberrant PI3K in Cancer	8	Fgf7;Esr1;Fgfr2	103	0.007	0.00053	0.030
R-HSA-109704	PI3K Cascade	6	Fgf7;Them4;Fgfr2	58	0.004	0.00062	0.030
R-HSA-199418	Negative regulation of the PI3K/AKT network	9	Fgf7;Them4;Esr1;Fgfr2	134	0.009	0.00068	0.030
R-HSA-5654727	Negative regulation of FGFR2 signaling	5	Fgf7;Fgfr2	41	0.003	0.00085	0.035
R-HSA-5654696	Downstream signaling of activated FGFR2	5	Fgf7;Fgfr2	42	0.003	0.00095	0.037
R-HSA-112399	IRS-mediated signalling	6	Fgf7;Them4;Fgfr2	65	0.004	0.00111	0.038
R-HSA-2428928	IRS-related events triggered by IGF1R	6	Fgf7;Them4;Fgfr2	69	0.005	0.00150	0.049
R-HSA-74752	Signaling by Insulin receptor	7	Atp6v1g2;Fgf7;Them4;Fgfr2	97	0.007	0.00178	0.049
R-HSA-2033519	Activated point mutants of FGFR2	4	Fgf7;Fgfr2	29	0.002	0.00182	0.049
R-HSA-2428924	IGF1R signaling cascade	6	Fgf7;Them4;Fgfr2	72	0.005	0.00185	0.049
R-HSA-74751	Insulin receptor signalling cascade	6	Fgf7;Them4;Fgfr2	72	0.005	0.00185	0.049
R-HSA-6811558	PI5P, PP2A and IER3 Regulate PI3K/AKT Signaling	8	Fgf7;Esr1;Fgfr2	126	0.009	0.00190	0.049
R-HSA-2404192	Signaling by Type 1 Insulin-like Growth Factor 1 Receptor (IGF1R)	6	Fgf7;Them4;Fgfr2	73	0.005	0.00198	0.049
R-HSA-190377	FGFR2b ligand binding and activation	3	Fgf7;Fgfr2	14	0.001	0.00203	0.049
R-HSA-2219528	PI3K/AKT Signaling in Cancer	8	Fgf7;Esr1;Fgfr2	134	0.009	0.00277	0.064
R-HSA-5654738	Signaling by FGFR2	6	Fgf7;Polr2a;Fgfr2	88	0.006	0.00491	0.108
R-HSA-9029569	NR1H3 & NR1H2 regulate gene expression linked to cholesterol transport and efflux	5	Abca1;Tnrc6c;Ep300	66	0.004	0.00651	0.131
R-HSA-5419276	Mitochondrial translation termination	6	Dap3;Mrpl38;Mrps10;Mrpl19;Mrps27;Mrpl40	94	0.006	0.00669	0.131
R-HSA-5389840	Mitochondrial translation elongation	6	Dap3;Mrpl38;Mrps10;Mrpl19;Mrps27;Mrpl40	94	0.006	0.00669	0.131
R-HSA-8939247	RUNX1 regulates transcription of genes involved in interleukin signaling	2	Lifr	7	0.000	0.00691	0.131
R-HSA-5368286	Mitochondrial translation initiation	6	Dap3;Mrpl38;Mrps10;Mrpl19;Mrps27;Mrpl40	96	0.007	0.00738	0.140
R-HSA-5368287	Mitochondrial translation	6	Dap3;Mrpl38;Mrps10;Mrpl19;Mrps27;Mrpl40	102	0.007	0.00975	0.175
R-HSA-9013695	NOTCH4 Intracellular Domain Regulates Transcription	3	Notch2;Ep300;Actg2	26	0.002	0.01114	0.179
R-HSA-2023837	Signaling by FGFR2 amplification mutants	2	Fgfr2	9	0.001	0.01116	0.179
R-HSA-427975	Proton/oligopeptide cotransporters	2	Slc15a4	9	0.001	0.01116	0.179
R-HSA-190236	Signaling by FGFR	6	Fgf7;Polr2a;Fgfr2	107	0.007	0.01210	0.194
R-HSA-164940	Nef mediated downregulation of MHC class I complex cell surface expression	2	Ap1s2	11	0.001	0.01629	0.261
R-HSA-9024446	NR1H2 and NR1H3-mediated signaling	5	Abca1;Tnrc6c;Ep300	85	0.006	0.01772	0.266
R-HSA-1912408	Pre-NOTCH Transcription and Translation	5	Tnrc6c;Notch2;Ep300	89	0.006	0.02110	0.316
R-HSA-1257604	PIP3 activates AKT signaling	11	Tnrc6c;Fgf7;RragB;Them4;Esr1;Fgfr2	316	0.022	0.02485	0.348
R-HSA-9006925	Intracellular signaling by second messengers	12	Tnrc6c;Fgf7;RragB;Them4;Esr1;Itpr3;Fgfr2	363	0.025	0.02771	0.388
R-HSA-2197563	NOTCH2 intracellular domain regulates transcription	2	Notch2;Ep300	16	0.001	0.03256	0.442
R-HSA-390522	Striated Muscle Contraction	3	Myh3;Myl1	40	0.003	0.03400	0.442
R-HSA-4043916	Defective MPI causes MPI-CDG (CDG-1b)	1	Mpi	2	0.000	0.03443	0.448
R-HSA-190375	FGFR2c ligand binding and activation	2	Fgfr2	17	0.001	0.03635	0.473
R-HSA-918233	TRAF3-dependent IRF activation pathway	2	Sike1;Ep300	17	0.001	0.03635	0.473
R-HSA-418457	cGMP effects	2	Kcnmb1;Pde2a	18	0.001	0.04029	0.484
R-HSA-189445	Metabolism of porphyrins	4	Hmbs;Blvrb;Uros;Slco2b1	73	0.005	0.04057	0.487
R-HSA-432720	Lysosome Vesicle Biogenesis	3	Ap1s2;Clvs1	43	0.003	0.04068	0.488

Table S9.
Mitochondrial associated genes changing in combination exposed newborn rat hearts vs controls.

Gene Symbol	Gene Description	Fold Change	p Value	Affymetrix ID	Entrez Gene ID
Bdh1	3-hydroxybutyrate dehydrogenase, type 1	2.29	0.0227	10754931	117099
Mtfp1	mitochondrial fission process 1	1.61	0.0455	10778038	289745
Tstd3	thiosulfate sulfurtransferase (rhodanese)-like domain containing 3	1.56	0.041	10875751	500420
Mrpl19	mitochondrial ribosomal protein L19	1.47	0.0168	10863420	297372
Rtn4ip1	reticulon 4 interacting protein 1	1.42	0.0334	10830630	309912
Bphl	biphenyl hydrolase like	1.39	0.0383	10798100	361239
Nif3l1	NGG1 interacting factor 3 like 1	1.38	0.0443	10923567	301431
Chchd4	coiled-coil-helix-coiled-coil-helix domain containing 4	1.37	0.025	10864100	312559
Gstk1	glutathione S-transferase kappa 1	1.37	0.0358	10855008	297029
Dus2	dihydrouridine synthase 2	1.36	0.0482	10807435	291978
Mrps27	mitochondrial ribosomal protein S27	1.36	0.027	10812722	361883
Bckdhb	branched chain keto acid dehydrogenase E1 subunit beta	1.35	0.0231	10911976	29711
Hccs	holocytochrome c synthase	1.35	0.0449	10933279	317444
Mrps10	mitochondrial ribosomal protein S10	1.34	0.0458	10926342	363187
Dguok	deoxyguanosine kinase	1.33	0.043	10863542	297389
Mmachc	methylmalonic aciduria (cobalamin deficiency) cblC type, with homocystinuria	1.33	0.036	10878905	313520
Mpst	mercaptopyruvate sulfurtransferase	1.33	0.0411	10897446	192172
Fam162a	family with sequence similarity 162, member A	1.32	0.0458	10751450	360721
Gcdh	glutaryl-CoA dehydrogenase	1.31	0.03	10806628	364975
Nudt5	nudix hydrolase 5	1.31	0.049	10799539	361274
Rexo2	RNA exonuclease 2	1.31	0.0443	10917095	300689
Mcat	malonyl-CoA-acyl carrier protein transacylase	1.3	0.0318	10898160	315173
Mrpl38	mitochondrial ribosomal protein L38	1.3	0.0453	10749132	303685
Dap3	death associated protein 3	1.29	0.0205	10824373	295238
Hmbs	hydroxymethylbilane synthase	1.29	0.0352	10916753	25709
Ndufc1	NADH:ubiquinone oxidoreductase subunit C1	1.29	0.0117	10823098	689938
Sirt5	sirtuin 5	1.28	0.0465	10797827	306840
Mrpl40	mitochondrial ribosomal protein L40	1.27	0.0288	10755987	287962
Ak2	adenylate kinase 2	1.26	0.0228	10872260	24184
Apoo	apolipoprotein O	1.26	0.0199	10934024	363474
L2hgdh	L-2-hydroxyglutarate dehydrogenase	1.26	0.0351	10890342	314196
Them4	thioesterase superfamily member 4	1.25	0.0443	10817208	361992
